# Type 2 cytokines act on enteric sensory neurons to regulate neuropeptide-driven host defense

**DOI:** 10.1126/science.adn9850

**Published:** 2025-05-22

**Authors:** Rocky M. Barilla, Clara Berard, Linyu Sun, Sumiti Sandhu, Sarah Zaghouani, Krishna S. Iyer, Gizem Altun, Chien-Wen Su, Jacques Deguine, Vasundhara Singh, Yu Hou, Kanupriya Kusumakar, Michael L. Rutlin, Meenakshi Rao, Habib Zaghouani, Hai Ning Shi, Ramnik J. Xavier, Vijay K. Kuchroo

**Affiliations:** 1 Gene Lay Institute of Immunology and Inflammation, Brigham and Women’s Hospital, Massachusetts General Hospital and Harvard Medical School, Boston, MA, USA.; 2 Division of Medical Sciences, Harvard Medical School, Boston, MA, USA.; 3 University of Minnesota Medical School, Minneapolis, MN, USA.; 4 Department of Experimental Neuroimmunology, Technical University of Munich School of Medicine, Munich, Germany.; 5 German Cancer Research Center (DKFZ), Heidelberg, Germany.; 6 Mucosal Immunology and Biology Research Center, Massachusetts General Hospital, Harvard Medical School, Charlestown, MA, USA.; 7 Broad Institute of MIT and Harvard, Cambridge, MA, USA.; 8 Liangzhu Laboratory, Zhejiang University, Hangzhou, China.; 9 Department of Pediatrics, Boston Children’s Hospital, Harvard Medical School, Boston, MA, USA.; 10 University of Missouri School of Medicine, Columbia, MO, USA.; 11 Department of Molecular Biology, Massachusetts General Hospital, Harvard Medical School, Boston, MA, USA.

## Abstract

Enteric nervous system (ENS)–derived neuropeptides modulate immune cell function, yet our understanding of how inflammatory cues directly influence enteric neuron responses during infection is considerably lacking. Here, we characterized a primary enteric sensory neuron (PSN) subset producing the neuropeptides neuromedin U (NMU) and calcitonin gene–related peptide β (CGRPβ) and coexpressing receptors for the type 2 cytokines interleukin-4 (IL-4) and IL-13. Type 2 cytokines amplified NMU and CGRPβ expression in PSNs both in vitro and in vivo, and this was abrogated by PSN-specific *Il13ra1* deletion. Deletion of *Il13ra1* in PSNs impaired host defense to the gastrointestinal helminth *Heligmosomoides polygyrus* and blunted muscularis immune responses. Co-administration of NMU23 and CGRPβ rescued helminth clearance deficits and restored anti-helminth immunity, highlighting the essential bidirectional neuroimmune cross-talk regulating intestinal type 2 inflammation.

Although the enteric nervous system (ENS) is best known for coordinating essential physiologic functions such as digestion and gut motility, recent evidence suggests that it is also a critical mediator in gut neuroimmune communication. We and others have demonstrated that ENS neuropeptides, such as neuromedin U (NMU), calcitonin gene –related peptide (CGRP), and vasoactive intestinal peptide (VIP), profoundly influence barrier immune responses and host defense against pathogens (1–4). For instance, NMU acts on type 2 innate lymphoid cells (ILC2s) that express NMUR1, amplifying their production of the type 2 cytokines interleukin-13 (IL-13) and IL-5 and promoting allergic inflammation in the lung and gut (1–3). Likewise, CGRP can have both pro- and anti-inflammatory effects depending on the tissue, cell type, and context (5–8). Regardless, studies investigating the role of neuroimmune communication in barrier defense have primarily focused on neuron-derived factors influencing immune cell function, leaving a considerable gap in our understanding of how enteric neurons respond to inflammatory stimuli, especially cytokines from local immune responses. Furthermore, enteric neurons embedded within the gut wall are uniquely susceptible to microbial exposure, inflammatory damage, and physical perturbations. This is illustrated in the context of infection with tissue-invading helminths such as *Heligmosomoides polygyrus*, which invade the gut wall, promoting local inflammation (9). Here, we found that enteric sensory neurons directly respond to type 2 cytokines during a tissue-invasive gastrointestinal helminth infection, consequently shaping the anti-helminth immune response.

## Primary enteric sensory neurons directly sense type 2 cytokines to amplify gut NMU and CGRPβ gene expression

**Fig. 1.**
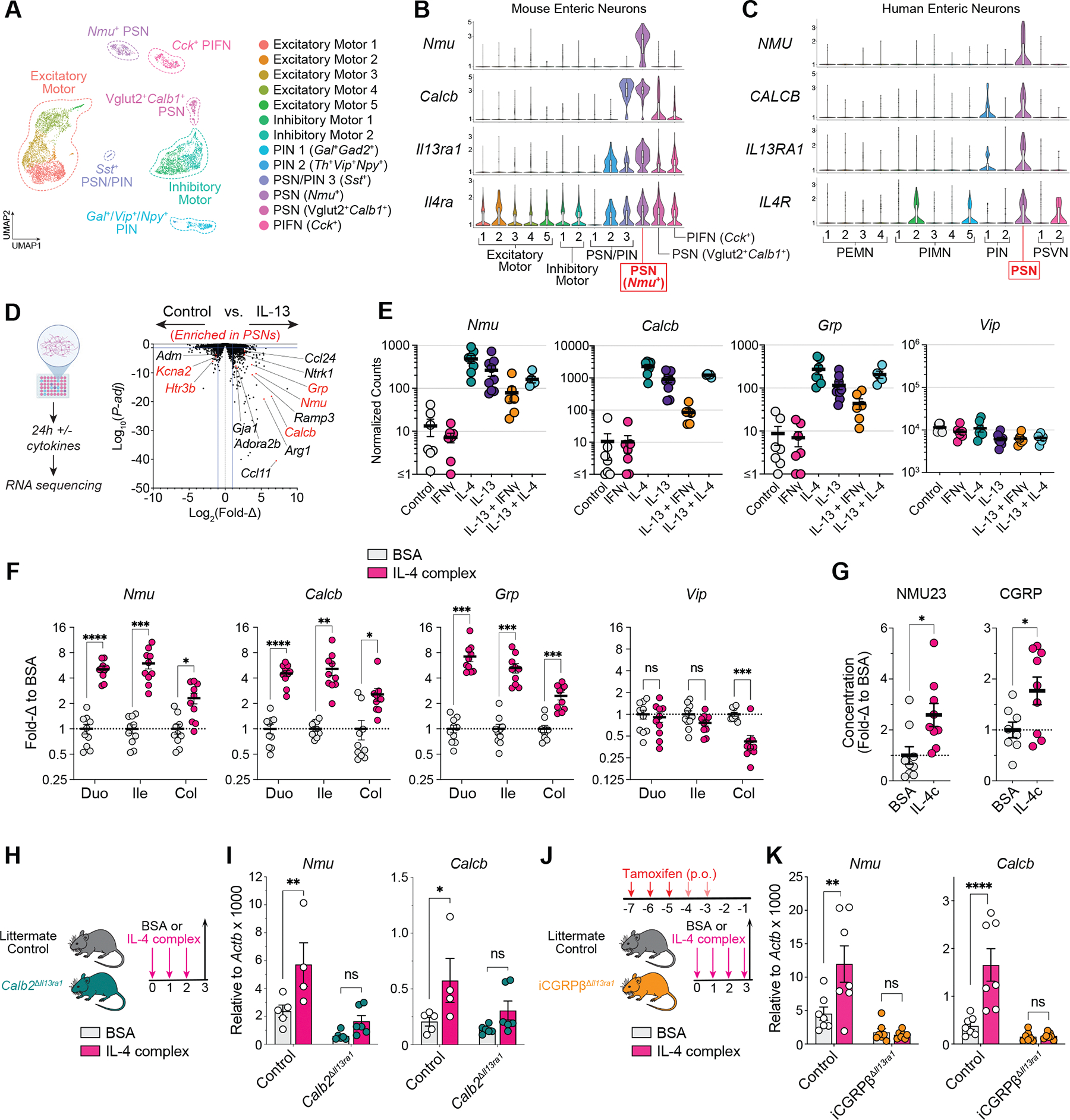
Type 2 cytokines directly amplify PSN neuropeptide expression. (**A** and **B**) scRNA-seq reanalysis of mouse SI enteric neurons integrated from three independent studies (10–12). (A) Uniform manifold approximation and projection (UMAP) and (B) violin plots showing indicated gene expression. (**C**) scRNA-seq of human colon enteric neurons from Drokhlyansky *et al*. (10). (**D** and **E**) Bulk RNA-seq analysis of in vitro enteric neuroglial cultures treated for 24 hours with indicated cytokines (50 ng ml^−1^ per cytokine). (D) Schematic and volcano plot. (E) DESeq2-normalized gene counts; lower cutoff was set to 1 for log-scale visualization (*n* = 5 to 9 mice). (**F** and **G**) C57Bl/6J mice were administered IL-4 complex intraperitoneally for 3 days (F) or 4 (G) days. (F) Duodenal (Duo), ileal (Ile), and colonic (Col) qPCR gene expression (*n* = 10 mice). (G) NMU23 and CGRP enzyme immunoassay (EIA) concentrations in ileal lysates. Shown is the fold change to bovine serum albumin (BSA) average per experiment (*n* = 8 to 9). (**H** and **I**) *Calb2*^Δ*Il13ra1*^ cKO mice and littermate controls administered IL-4 complex. (H) Schematic. (I) Duodenal qPCR (*n* = 4 to 6 mice). (**J** and **K**) iCGRPβ^Δ*Il13ra1*^ cKO mice and littermate controls gavaged with three or five consecutive doses of tamoxifen and administered IL-4 complex for 4 days. (J) Schematic. (K) Duodenal qPCR (*n* = 7 mice). Data were pooled from two independent experiments [(F), (G), (I), and (K)] or are representative of three independent experiments (E). Data points represent single mice [(F), (G), (I), and (K)] or distinct in vitro cultures derived from single neonatal mice [(D) and (E)]. qPCR gene expression is relative to *Actb*, represented as 2^–ΔCt^ × 1000 [(I) and (K)] or fold change to the BSA group per experiment (F). Each graph indicates the mean ± SEM of replicates. ns, not significant; *P < 0.05; **P < 0.01; ***P < 0.001; ****P < 0.0001, two-way ANOVA with Sidak’s correction [(F), (I), and (K)] or two-tailed Welch’s *t* test (G). PIN, putative interneuron; PIFN, putative intestinofugal neuron; PEMN, putative excitatory motor neuron; PIMN, putative inhibitory motor neuron; PSVN, putative secretomotor/vasodilator neuron.

To identify cytokine receptors expressed by enteric neurons, we reanalyzed three publicly available single-cell RNA sequencing (scRNA-seq) datasets sampling mouse small intestine (SI) enteric neurons (10–12) and one dataset sampling human colon enteric neurons (10) (fig. S1, A and B). After integrating the mouse SI datasets, we observed 13 distinct clusters resembling the 12 to 13 annotated clusters from each individual dataset (Fig. 1A and fig. S1C). *Nmu* and *Calcb*, encoding the immunomodulatory neuropeptides NMU and CGRPβ, respectively, were exclusively coexpressed by a single cluster of putative primary enteric sensory neurons (PSNs), also known as intrinsic primary afferent neurons (IPANs) (Fig. 1B and fig. S1, C and D). Moreover, we found that *Nmu*^+^ PSNs were enriched in immune-associated genes such as *Il13ra1*, *Il4ra*, *Jak1*, and *Stat6*, encoding the IL-4/IL-13 heterodimeric receptor and its essential signaling components, and *Il7*, encoding a critical ILC2 and memory T cell survival factor (Fig. 1B and fig. S1, D to F). Consistently, human colonic *NMU*^+^*CALCB*^+^ enteric sensory neurons also showed enrichment of *IL13RA1* and *IL4R* (Fig. 1C). In immune cells, IL-4 signals through a distinct heterodimeric receptor composed of IL-4 receptor α (IL-4Rα) and the IL-2 common γ-chain (γ_c_). However, we did not observe substantial expression of *Il2rg* in enteric neurons (fig. S1F).

To assess the functional responses of PSN cytokine receptor activation, we first used neural crest progenitor–derived enteric neuron cultures, which display IL-13Rα1 expression localized to enteric neuron cell bodies (Fig. 1D and fig. S2A). Both IL-4 and IL-13, but not interferon-γ (IFNγ), amplified the expression of the PSN-associated neuropeptide genes *Nmu, Calcb,* and *Grp* by an average of ~20- to 200-fold compared with controls (Fig. 1, D and E, and fig. S2B). This up-regulation was STAT6 dependent and was not comparably observed in dorsal root ganglia (DRG) neuron cultures (fig. S2, C to I). Consistent with our in vitro data, administering three daily doses of recombinant IL-4:anti-IL-4 complex to wild-type mice sufficiently augmented gastrointestinal NMU and CGRPβ gene and peptide expression in vivo (Fig. 1, F and G, and fig. S3, A and B). IL-13 complex treatment similarly amplified SI *Nmu* and *Calcb* expression (fig. S3, C and D), although the *Calcb* induction was lower than that observed in IL-4 complex–treated mice (fig. S3D). Type 2 cytokine –induced neuropeptide expression was not observed in other digestive, mucosal, or nervous tissues sampled, including liver, lung, DRG, and brainstem (fig. S3, C and D). Moreover, type 2 cytokine neuropeptide induction was not directly caused by enteric neuron proliferation (fig. S3, E to J).

To determine whether this effect was caused by direct enteric neuron cytokine sensing, we used *Calb2*-cre mice to target neurons expressing calretinin/*Calb2*, including *Nmu*^+^ and *Sst*^+^ PSNs and several subsets of excitatory motor neurons (figs. S1C and S4, A and B). We also developed tamoxifen-inducible *Calcb-*ERT-cre (iCGRPβ-ERT-cre) mice to more specifically target PSNs and to circumvent possible effects on ENS development. To validate the specificity of these models, we crossed nuclear green fluorescent protein (GFP) reporter mice (*R26-LSL-Sun1-sfGFP*) to both the *Calb2-*cre and the iCGRPβ-ERT-cre models, observing selective and robust nuclear GFP expression in enteric neurons from the duodenal, ileal, and colonic myenteric and submucosal plexi of both cre lines, with little to no GFP expression in the gut epithelia and lamina propria (LP) (fig. S4, C to M). Morphological analysis showed that iCGRPβ-ERT-cre –labeled GFP^+^ neurons had larger cell bodies than GFP^−^ neurons (fig. S4, I and J), in agreement with previous reports showing that CGRP^+^ enteric sensory neurons have larger cell bodies (13). We next generated PSN-targeting *Il13ra1* conditional knockout (cKO) mice by crossing *Il13ra1*^*flox/flox*^ mice into either *Calb2*-cre (*Calb2*^Δ*Il13ra1*^ cKO) or iCGRPβ-ERT-cre (iCGRPβ^Δ*Il13ra1*^ cKO) mice. The administration of IL-4 complex failed to induce signature *Nmu* and *Calcb* expression in the duodena of either *Calb2*^ΔIl13ra1^ cKO or iCGRPβ^Δ*Il13ra1*^ cKO mice (Fig. 1, H and I), indicating that enteric neurons recognize IL-4 directly and specifically through the IL-4Rα/IL-13Rα1 heterodimeric receptor. Furthermore, we found no changes to duodenal CGRPα/*Calca* and *Vip* expression or intestinal size after IL-4 complex treatment in either control or iCGRPβ^Δ*Il13ra1*^ cKO mice (fig. S5, A and B), and no differences in general IL-4 complex–induced inflammatory phenotypes such as splenomegaly, immune cell *Chil3* up-regulation, and epithelial cell *Retnlb* up-regulation between IL-4 complex–treated *Calb2*^Δ*Il13ra1*^ cKO, iCGRPβ^Δ*Il13ra1*^ cKO, and control mice (fig. S5, F to H).

## PSN *Il13ra1* ablation impairs host defense to gastrointestinal helminths

**Fig. 2.**
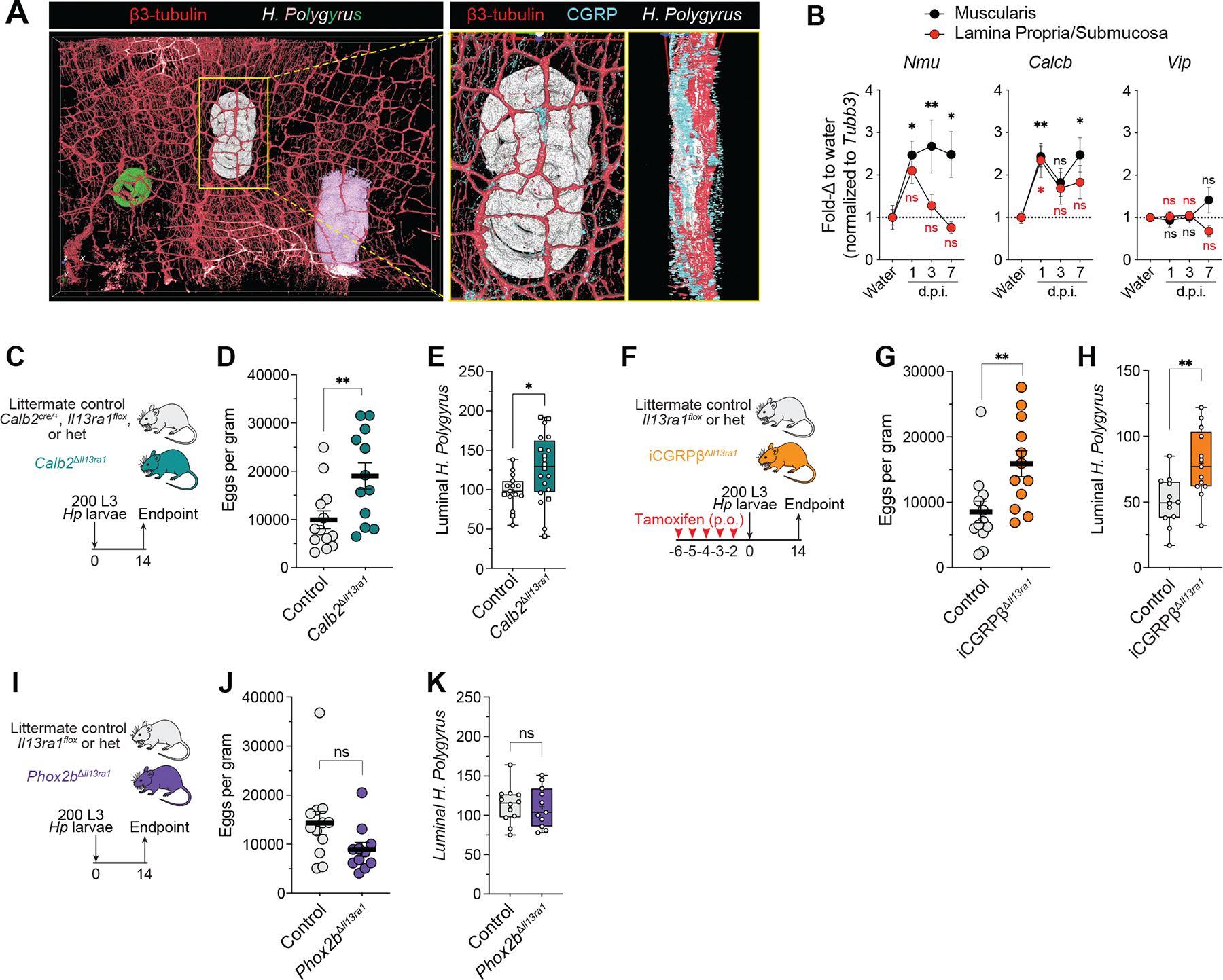
PSN-specific *Il13ra1* ablation impairs host defense to *H. polygyrus*. (**A**) Confocal imaging and three-dimensional rendering (Aivia) of Ce3D-cleared duodenal muscularis at 6 d.p.i. Autofluorescent *H. polygyrus* larvae are pseudocolored green, white, and pink; enteric neurons/β3-tubulin are shown in red; and CGRP is shown in cyan. Insets (yellow boxes) show magnified white larvae from the top (left inset) and the side (right inset). (**B**) Duodenal qPCR of muscularis (black) and lamina propria/submucosa (red) at the indicated d.p.i. Gene expression is relative to *Tubb3*, represented as fold change to the water group per organ. Statistics compare indicated d.p.i. against the water group per organ. Data were pooled from four independent time points (*n* = 3 to 4 mice per time point). (**C** to **E**) *H. polygyrus*–infected *Calb2*^Δ*Il13ra1*^ cKO and littermate control (*Calb2*^cre/+^, *Il13ra1*^*flox*^, or heterozygous) mice at 14 d.p.i. (C) Schematic. (D) Fecundity (*n* = 12 to 13 mice). (E) Worm count (*n* = 15 to 20 mice). Squares indicate mice that received daily PBS. (**F** to **H**) *H. polygyrus*–infected tamoxifen-induced iCGRPβ^Δ*Il13ra1*^ cKO and littermate control mice. (F) Schematic. (G) Fecundity (*n* = 12 mice). (H) Worm count (*n* = 12 to 13 mice). (**I** to **K**) *H. polygyrus*-infected *Phox2b*^Δ*Il13ra1*^ cKO and littermate control mice at 14 d.p.i. (I) Schematic. (J) Fecundity (*n* = 11 to 12 mice). (K) Worm count (*n* = 11 to 12 mice). Data were pooled from two [(D), (H), (J), and (K)], three (G), or four (E) independent experiments. Each data point represents a single mouse [(C) to (K)], and each bar and scatter plot indicate the mean ± SEM of replicates. ns, not significant; *P < 0.05; **P < 0.01, two-tailed Welch’s *t* test [(E), (H), and (K)], two-tailed Mann-Whitney test [(D), (G), and (J)], or two-way ANOVA with Dunnett’s correction (B).

To investigate the roles of PSN type 2 cytokine receptor signaling during intestinal inflammation, we used the *H. polygyrus* gastrointestinal helminth infection model, which elicits strong type 2 immune responses in the gut. We observed *IL-13Rα1* immunoreactivity in the soma of *Calb2*-cre –labeled enteric neurons from the myenteric plexi of *H. polygyrus*–infected mice (fig. S6, A and B). *H. polygyrus* displays a complex, multistep life cycle within the host, including a tissue-dwelling phase at ~0 to 7 days postinfection (d.p.i.), when larvae burrow into the muscularis to grow and molt, and a luminal phase (8 to 14+ d.p.i.), when mature worms re-emerge in the lumen to mate and lay eggs. During the tissue-dwelling phase, muscularis-implanted *H. polygyrus* larvae were situated near myenteric plexus neurons, including CGRP^+^ neuronal fibers (Fig. 2A and movie S1), eliciting increased *Nmu* and *Calcb*, but not *Vip*, expression in the duodenal muscularis as early as 1 d.p.i. (Fig. 2B and fig. S6C). Furthermore, at 7 d.p.i., individually implanted larvae in the duodenal wall of *Calb2*^Δ*Il13ra1*^ cKO mice occupied ~20% greater areas than those of littermate controls, with no difference in total number (fig. S6, D to F). During the luminal phase of infection, both *Calb2*^Δ*Il13ra1*^ cKO and iCGRPβ^Δ*Il13ra1*^ cKO mice failed to effectively control the helminth infection, displaying greater *H. polygyrus* fecundity and luminal worm burdens compared with their respective littermate controls (Fig. 2, C to H, and fig. S6, G and H). Moreover, these differences in worm burden and fecundity could not be explained by differences in gastrointestinal motility (fig. S6, I to K).

To exclude the possibility of extrinsic neuronal involvement in our observed phenotypes, we examined cre expression in gut-extrinsic ganglia by crossing *R26-LSL-Sun1-sfGFP* mice to *Calb2-*cre, iCGRPβ-ERT-cre, and *Phox2b*-cre BAC transgenic mice (hereafter, *Phox2b-*cre), which have been reported to express cre primarily in the hindbrain and nodose ganglia but not in the ENS and peripheral ganglia (14). Whereas *Calb2*-cre and *Phox2b*-cre reporter–crossed mice showed virtually no sensory neuron GFP labeling in DRG of the thoracic splanchnic nerve, iCGRPβ-ERT-cre reporter mice showed substantial GFP labeling in DRG neurons, in addition to enteric sensory neurons (fig. S7, A to C). Conversely, *Phox2b*-cre mice displayed extensive cre-mediated recombination in the autonomic nervous system, having more GFP^+^ neurons in the vagal sensory nodose-petrosal-jugular ganglia (N-JG) and the sympathetic celiac ganglia–superior mesenteric ganglia (CG-SMG) than both *Calb2*-cre and iCGRPβ-ERT-cre reporter–crossed mice (fig. S7, D to F). Contrary to previous reports (14), we detected sparse (<10%) *Phox2b*-cre recombination in the myenteric plexus (fig. S7, G and H). However, the labeled neurons were calretinin^−^ and were not larger than GFP^−^ neurons (fig. S7, I to K), suggesting that *Phox2b*-cre mice do not target PSNs. When *Phox2b*-cre mice were crossed to *Il13ra1* floxed mice (*Phox2b*^Δ*Il13ra1*^ cKO) and infected with *H. polygyrus*, we observed no differences in luminal worm burden or helminth fecundity at 14 d.p.i. (Fig. 2, I to K), suggesting that *Il13ra1* ablation in enteric neurons, but not in gut-extrinsic autonomic neurons, impairs host control of *H. polygyrus* infection.

## PSN neuropeptides rescue impaired *H. polygyrus* clearance of PSN *Il13ra1* cKO mice

**Fig. 3.**
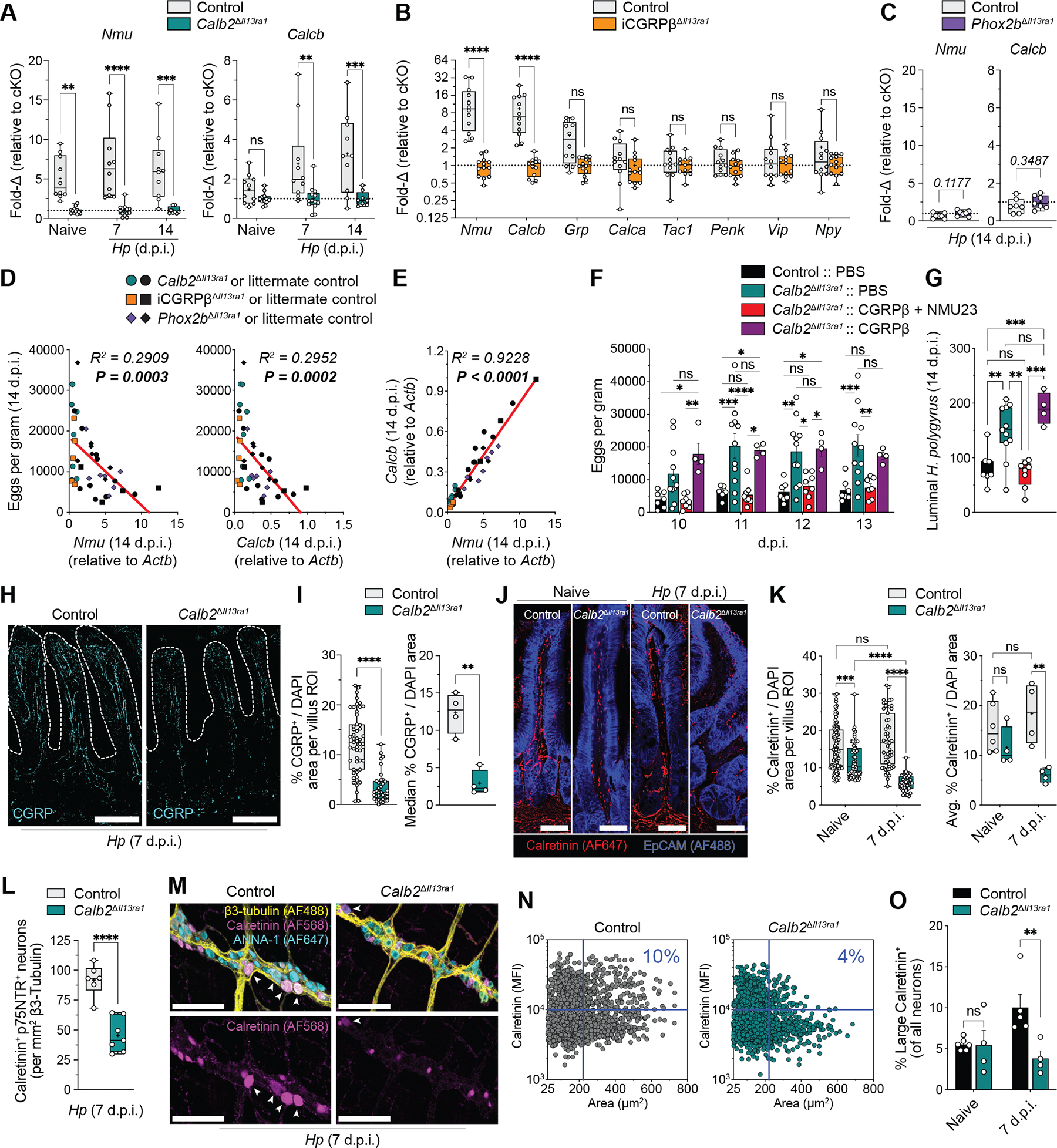
Type 2 cytokines maintain PSN population and neuropeptide expression. (**A** to **C**) Duodenal qPCR data from the indicated *Calb2*^Δ*Il13ra1*^ cKO mice (*n* = 8 to 14 mice) (A), 14 d.p.i. tamoxifen-induced iCGRPβ^Δ*Il13ra1*^ cKO mice (*n* = 12 to 13 mice) (B), and 14 d.p.i. *Phox2b*^Δ*Il13ra1*^ cKO mice (*n* = 7 mice) (C) and respective littermate controls. (**D** and **E**) Scatter plots correlating 14 d.p.i. duodenal qPCR gene expression (*x* axes) and fecundity (*y* axes) (D) or duodenal qPCR expression of indicated genes from matched experiments (E) (*n* = 41 mice). (**F** and **G**) *Calb2*^Δ*Il13ra1*^ cKO mice administered the indicated neuropeptides intraperitoneally (2 nmoles each per injection) or PBS daily. (F) Fecundity on indicated d.p.i. (G) 14 d.p.i. worm count (*n* = 4 to 11 mice). (**H** to **K**) Confocal microscopy of duodenal cross-sections. (H) Representative villi at 7 d.p.i. (I) Percentage CGRP^+^ area of total 4ʹ,6-diamidino-2-phenylindole (DAPI) area per villus (left) and per mouse (right) (*n* = 4 mice). (J) Representative villi. (K) Percentage calretinin^+^ area of total DAPI area per villus (left) and per mouse (right) (*n* = 4 to 6 mice). (**L** to **O**) Whole-mount confocal imaging of 7 d.p.i. duodenal myenteric plexi. (L) Calretinin^+^p75NTR^+^ neurons per square millimeter of β3-tubulin^+^ area (*n =* 6 to 7 mice). (M) Representative 7 d.p.i. myenteric plexi immunostained for β3-tubulin (yellow), calretinin (magenta), and ANNA-1 (neuronal nuclei/cell body marker; cyan). Arrows indicate large calretinin^+^ PSNs. (N) Scatter plot of pooled segmented neurons at 7 d.p.i. from littermate control (*n* = 5) and *Calb2*^Δ*Il13ra1*^ cKO (*n* = 4) mice. (O) Percentage of large calretinin^+^ neurons (*n* = 4 to 6 mice). Data were pooled from two [(B), (K), (O)], three [(D) to (G)], or six (A) independent experiments or are representative of two or more independent experiments [(C), (I), and (L)]. Data points represent single mice or individual villi [(I), left; (K), left]. Scale bars, 100 μm [(H), (J), and (M)]. qPCR gene expression is relative to *Actb*, represented as 2^–ΔCt^ × 1000 [(D) and (E)] or fold change to the cKO group per experiment [(A) to (C)]. Each bar graph indicates the mean ± SEM of replicates. ns, not significant; *P < 0.05; **P < 0.01; ***P < 0.001; ****P < 0.0001, two-way ANOVA with Sidak’s correction [(A), (B), and (O)] or Tukey’s correction (K), mixed-effects analysis with Tukey’s correction (F), one-way ANOVA with Tukey’s correction (G), two-tailed *t* test [(C), (I), and (L)], or simple linear regression [(D) and (E)].

We next investigated how PSN *Il13ra1* ablation influences gut neuropeptide expression. Although steady-state *Nmu* expression was diminished specifically in the duodena of *Calb2*^Δ*Il13ra1*^ cKO mice and iCGRPβ^Δ*Il13ra1*^ cKO mice (Fig. 3A and fig. S8, A and B), there were no differences in baseline duodenal *Calcb* expression among *Calb2*^Δ*Il13ra1*^ cKO mice, iCGRPβ^Δ*Il13ra1*^ cKO mice, and littermate controls (Fig. 3A and fig. S8, A and B). However, in the helminth-infected gut, expression of *Nmu* and *Calcb*, but not of other typical ENS neuropeptides, was diminished in all sampled SI regions of *Calb2*^Δ*Il13ra1*^ and iCGRPβ^Δ*Il13ra1*^ cKO mice (Fig. 3, A and B, and fig. S8, C to H) but was not altered in *Phox2b*^Δ*Il13ra1*^ cKO mice (Fig. 3C).

We noticed a strong negative correlation between *H. polygyrus* fecundity and both duodenal *Nmu* and *Calcb* expression at 14 d.p.i. across mice from all linked experimental litters (Fig. 3D) as well as across the littermate control mice only (fig. S8I), suggesting that natural duodenal *Nmu* and *Calcb* variation may influence *H. polygyrus* fecundity in wild-type mice. This PSN neuropeptide anticorrelation was also observed for implanted larval size at 7 d.p.i. (fig. S8J). Furthermore, duodenal *Nmu* and *Calcb* expression exhibited a robust positive correlation with each other (*R*^2^ = 0.89 to 0.92, *P* < 0.0001) at steady state and during helminth infection (Fig. 3E and fig. S8K), suggesting that their expression is tightly co-regulated. Therefore, we tested whether PSN neuropeptide co-administration could rescue the helminth clearance deficits of *Calb2*^Δ*Il13ra1*^ cKO mice, finding that daily co-administration of equimolar NMU23 and CGRPβ, but not administration of CGRPβ alone, reduced both *H. polygyrus* fecundity and worm burden to values similar to those of phosphate-buffered saline (PBS)–administered littermate control mice (Fig. 3, F and G, and fig. S8L). However, NMU23 and CGRPβ co-administration did not restore the impaired duodenal *Nmu* and *Calcb* expression of *Calb2*^Δ*Il13ra1*^ cKO mice (fig. S8M).

We next investigated whether enteric neuron *Il13ra1* ablation influences PSN villus innervation and abundance. *H. polygyrus*–infected *Calb2*^Δ*Il13ra1*^ cKO mice displayed lower CGRP^+^ and calretinin^+^ fiber densities per villus and per mouse compared with controls at 7 d.p.i. (Fig. 3, H to K). However, there were no differences in calretinin^+^ fiber density per mouse in the absence of infection (Fig. 3K). Furthermore, *Calb2*^Δ*Il13ra1*^ cKO mice displayed fewer calretinin^+^p75NTR^+^ neurons than did littermate controls at 7 d.p.i. (Fig. 3L, and fig. S9, A to E), suggesting a loss of *Nmu*^+^ PSNs coexpressing calretinin and p75NTR/*Ngfr* after infection (fig. S4, A and B).

Because iCGRPβ-ERT-cre reporter–labeled GFP^+^ neurons were larger than GFP^−^ neurons (fig. S4I) and because large (above the third quartile in soma size per experiment) calretinin^+^ neurons displayed greater p75NTR expression than small calretinin^+^ and calretinin^−^ enteric neurons (fig. S9F), we used large calretinin^+^ neurons to discriminate PSNs. Consistently, *Calb2*^Δ*Il13ra1*^ cKO mice had lower percentages and total numbers of PSNs in the myenteric plexus compared with littermate controls at 7 d.p.i., but there were no differences in uninfected mice (Fig. 3, M to O, and fig. S9, G to I), suggesting that enteric neuron *Il13ra1* ablation selectively depletes PSNs in the helminth-infected myenteric plexus, leading to diminished NMU and CGRPβ gene expression and loss of intrinsic sensory neuron fibers to the mucosal villi.

## PSN neuropeptide expression influences muscularis-specific type 2 immune responses

**Fig. 4.**
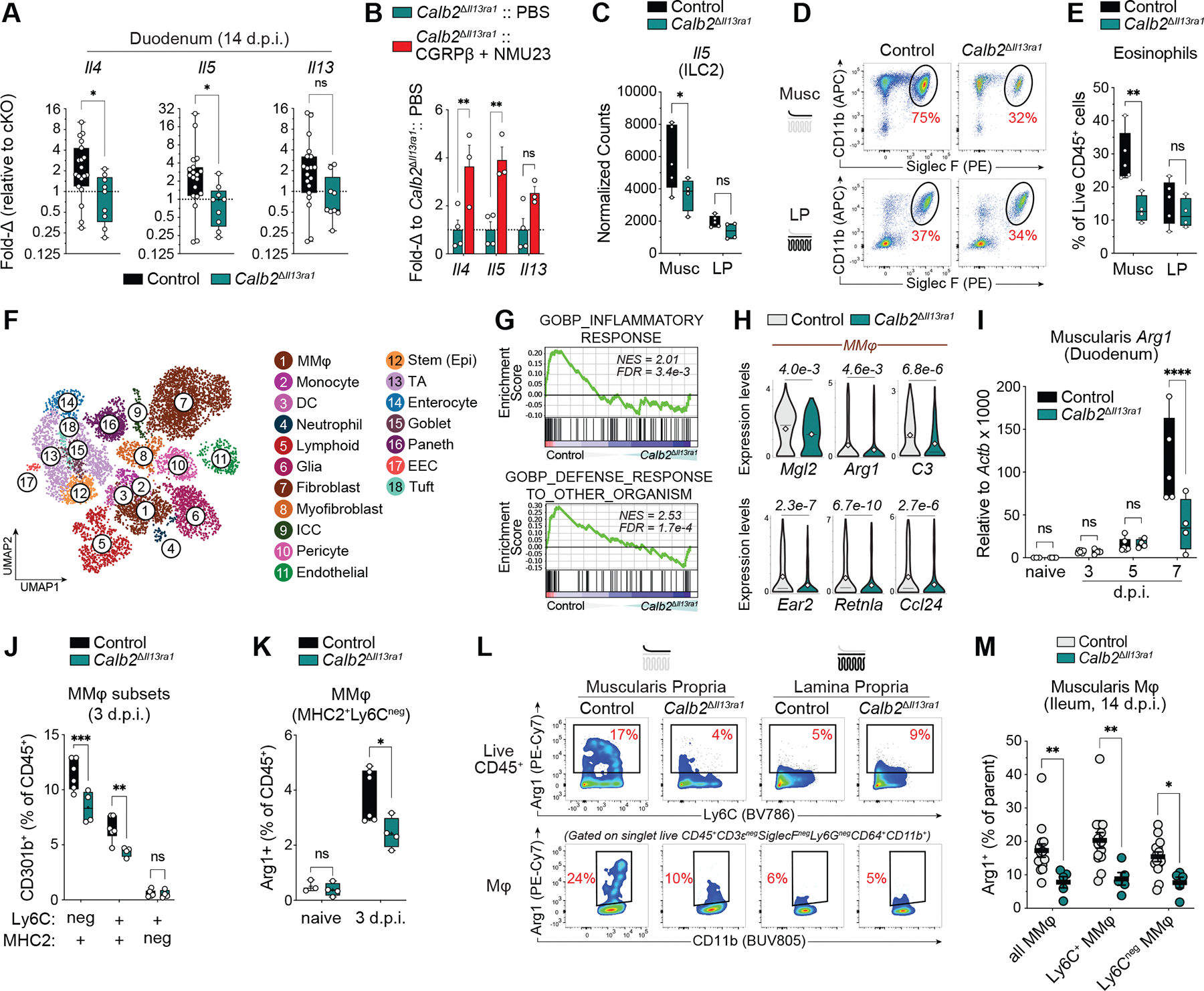
PSN IL-4/IL-13 sensing promotes muscularis-specific type 2 immune responses. (**A**) Duodenal qPCR gene expression at 14 d.p.i. (*n* = 9 to 19 mice). (**B**) Duodenal qPCR gene expression in mice with daily CGRPβ and NMU23 co-administration as in Fig. 3, F and G (*n* = 3 to 4 mice). (**C**) DESeq2-normalized bulk RNA-seq counts of ileal muscularis and LP ILC2s at 7 d.p.i. as gated in fig. S11A (*n* = 4 to 5 mice). (**D** and **E**) Flow cytometric analysis of ileal muscularis and LP immune cells at 7 d.p.i. (D) Representative flow plots gated on single live CD45^+^ CD90.2^–^Lineage(Ter-119, NK1.1, CD19, B220)^−^ cells. (E) Percentage of muscularis and LP eosinophils (as gated in fig. S11A) of all CD45^+^ cells (*n* = 4 to 5 mice). (**F** to **H**) scRNA-seq of ileal muscularis (input 50% enriched for stroma or glia as in fig. S13A) and duodenal epithelium at 7 d.p.i. (F) UMAP embedding of 8270 individual cells pooled from two mice per group using general-level cluster annotation. (G) Gene set enrichment analysis (GSEA) plots of general MMφ cluster, depicting Gene Ontology gene sets from MSigDB. (H) Violin plots of general MMφ clusters. (**I**) Duodenal muscularis qPCR gene expression at indicated d.p.i. (*n* = 3 to 6 mice per time point). (**J** and **K**) Flow cytometry of duodenal MMφ (as gated in fig. S11C) at 3 d.p.i. (J) Percentage CD301b^+^ of CD45^+^ cells in indicated MMφ subsets at 3 d.p.i. (*n* = 4 to 6 mice). (K) Percentage Arg1^+^ MMφ (MHC2^+^Ly6C^–^) of CD45^+^ immune cells (*n* = 3 to 6 mice per time point). (**L** and **M**) Flow cytometry of ileal muscularis and LP immune cells at 14 d.p.i. (L) Representative flow plots; shown are total immune cells (top) and macrophages (bottom). (M) Frequency of Arg1^+^ cells in indicated MMφ subsets (*n* = 5 to 14 mice). Data were pooled from two [(A), (K), and (M)] or four (I) independent experiments or are representative of two or more independent experiments [(B) and (E)]. Each data point represents a single mouse. qPCR gene expression is relative to *Actb*, represented as 2^–ΔCt^ × 1000 (I) or fold change to the cKO group (A) or PBS-treated cKO group (B) per experiment. Each bar graph indicates the mean ± SEM of replicates. ns, not significant; *P < 0.05; **P < 0.01; ***P < 0.001; ****P < 0.0001, two-tailed *t* test (A), two-way ANOVA with Sidak’s correction [(B), (C), (E), (I) to (K), and (M)], or Wilcoxon rank-sum test adjusted *P* value (H).

Assessing the effects of PSN *Il13ra1* ablation on anti-helminthic immunity, we found that *Calb2*^Δ*Il13ra1*^ cKO mice displayed blunted duodenal *Il4* and *Il5* expression at 14 d.p.i. (Fig. 4A), which was independent of T cell–priming differences in the mesenteric lymph nodes (fig. S10, A to F). These impairments could be rescued with co-administration of NMU23 and CGRPβ (Fig. 4B). Conversely, *Phox2b*^Δ*Il13ra1*^ cKO mice displayed enhanced duodenal *Il4* and *Il13* expression compared with controls at 14 d.p.i. (fig. S10G), suggesting that IL-13Rα1 signaling in intrinsic and extrinsic neurons has distinct effects on gastrointestinal type 2 immune responses.

Because ILC2s are a predominant source of IL-5 in the helminth-infected gut (15) and respond to NMU and CGRP (1–3, 5, 16, 17), we performed bulk RNA-seq on purified ILC2s from the muscularis and LP of *Calb2*^ΔIl13ra1^ cKO and littermate control mice at 7 d.p.i. (fig. S11A). Comparing control ILC2s from each compartment, muscularis ILC2s were enriched in *Calca* and CGRP receptor genes (*Calcrl*, *Ramp1*, and *Ramp3*) (fig. S12A), whereas LP ILC2s showed greater NMU and VIP receptor gene expression (fig. S12A). Accordingly, muscularis ILC2s expressed genes up-regulated by or downstream of CGRP receptor signaling and genes associated with ST2^+^ “natural” nILC2s (5), whereas LP ILC2s were enriched in genes associated with IL-25R^+^ “inflammatory” iILC2s (5, 17, 18) (fig. S12B). Furthermore, muscularis *Calb2*^ΔIl13ra1^ cKO ILC2s displayed lower expression of *Calcrl*, whereas LP *Calb2*^ΔIl13ra1^ cKO ILC2s showed lower expression of *Nmur1* compared with their respective control ILC2s (fig. S12C), suggesting that *Calb2*^ΔIl13ra1^ cKO neuropeptide deficits may influence immunity in a site-specific manner. Confirming this, *Calb2*^ΔIl13ra1^ cKO ILC2s from the muscularis, but not the LP, displayed greater cell cycle gene expression compared with ILC2s from littermate controls at 7 d.p.i. (fig. S12D). These results are consistent with CGRP’s function as a potent inhibitor of ILC2 proliferation (5, 16, 17) and are in agreement with the CGRPβ gene expression deficits shown in *Calb2*^ΔIl13ra1^ cKO mice. Nevertheless, we saw no differences in duodenal muscularis ILC2 abundance at steady state or at 5 d.p.i. (figs. S11B and S12, E and F).

*Il5* was the most highly expressed ILC2 cytokine in muscularis ILC2s, and its expression was reduced in muscularis, but not LP, *Calb2*^Δ*Il13ra1*^ cKO ILC2s compared with control ILC2s (Fig. 4C and fig. S12, G and H). IL-5 is a well-studied differentiation and maturation factor for eosinophils, which are a major source of IL-4 during intestinal helminth infection (19) and play a role in killing entrapped *H. polygyrus* larvae (20). Eosinophils were most heavily recruited to the duodenal muscularis during the late tissue-dwelling phase and expressed greater *Il4* and *Prg2* than those recruited to the LP (figs. S11C and S12, I to K). Furthermore, the abundance and proportion of eosinophils recruited to the muscularis, but not the LP, were reduced in the proximal and distal SI of *Calb2*^Δ*Il13ra1*^ cKO mice compared with littermate controls at 7 d.p.i. (Fig. 4, D and E, and fig. S12L).

## CGRPβ synergizes with IL-4 to induce muscularis macrophage arginase-1 expression

To more extensively characterize the helminth-infected muscularis, we performed scRNA-seq on *Calb2*^Δ*Il13ra1*^ cKO and littermate control mice at 7 d.p.i. (Fig. 4F and fig. S13, A to F), sampling muscularis cell populations as well as epithelial cell populations, which also displayed impaired type 2 effector responses (fig. S14, A to D). Muscularis macrophages (MMφ) exhibited the most differentially expressed genes, with control MMφ enriched in gene sets involved in host defense (Fig. 4G and fig. S14, G to K), including genes encoding critical type 2 mediators and alternatively activated macrophage (AAMφ) markers with roles in anti-helminth immunity and tissue repair (*Arg1*, *Mgl2*, *Ear2*, and *Retnla*) (21, 22), eosinophil chemotaxis (*Ccl24*), and macrophage adhesion to *H. polygyrus* larvae (*C3*) (23, 24) (Fig. 4H and fig. S14K). *Arg1* gene expression was reduced at 7 d.p.i. in the duodenal muscularis of *Calb2*^Δ*Il13ra1*^ cKO mice (Fig. 4I), which also had lower percentages of CD301b/*Mgl2*^+^ and arginase-1–positive (Arg1^+^) MMφ compared with controls at 3 d.p.i. (Fig. 4, J and K, and fig. S15A). Additionally, *Calb2*^Δ*Il13ra1*^ cKO mice displayed reduced duodenal *Arg1* during the luminal phase of infection, with lower percentages of Arg1–expressing MMφ and total immune cells in the ileal muscularis, but not LP, compared with controls (Fig. 4, L and M, and fig. S15, B to D).

**Fig. 5.**
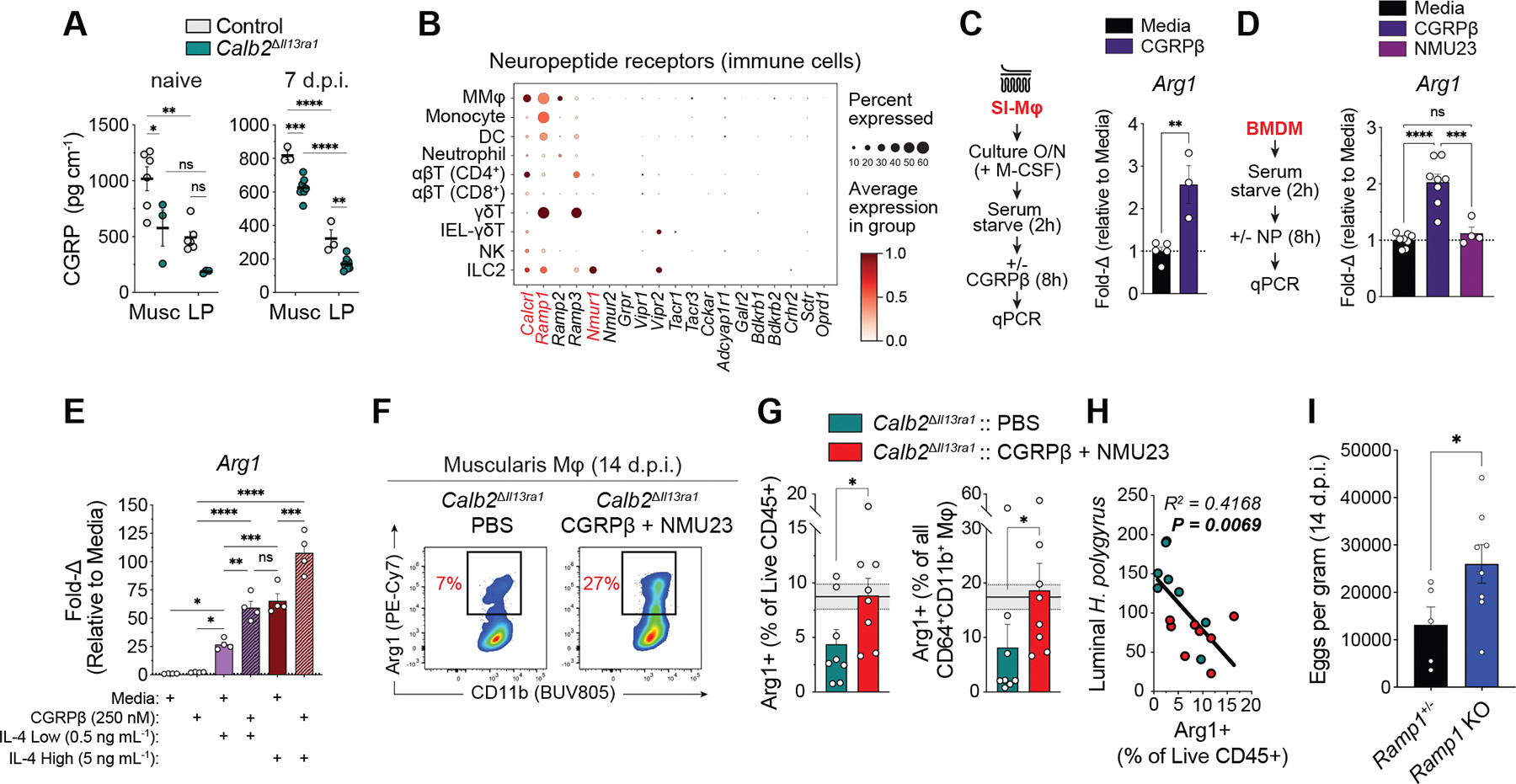
CGRPβ synergizes with type 2 cytokines to drive MMφ Arg1. (**A**) CGRP EIA peptide concentration (*n* = 3 to 8 mice). (**B**) scRNA-seq dot plot across general immune cell clusters (including lymphoid cell subclusters) from the dataset shown in Fig. 4F. (**C** to **E**) qPCR gene expression in duodenal SI-Mφ cultures (*n* = 3 to 5 wells) (C) or BMDM cultures (*n* = 4 to 8 wells) (D and E) treated for 8 hours with or without indicated neuropeptides (250 nM each) or IL-4 concentrations. (**F** to **H**) Flow cytometry of 14 d.p.i. ileal muscularis immune cells. (F) Representative flow plots depicting percentage Arg1^+^ MMφ (single live CD45^+^ CD3ε^–^Ly6G^–^Siglec-F^–^CD64^+^ CD11b^+^). (G) Percentage Arg1^+^ muscularis immune cells (left) and MMφ (right) (*n* = 8 mice). Solid and dotted lines with grayed area represent the mean ± SEM of control mice at 14 d.p.i. pooled from four independent experiments (*n* = 16 mice). (H) Scatter plot correlating 14 d.p.i percentage Arg1^+^ muscularis immune cells (*x* axis) with worm count (*y* axis) (*n* = 16 mice). (**I**) The 14 d.p.i. fecundity of *Ramp1* KO and littermate control (*Ramp1*^+/−^) mice (*n* = 5 to 8 mice). Data were pooled from two independent experiments [(G) to (I)] or are representative of two or more independent experiments [(A), (C), (D), and (E)]. Data points represent individual mice [(A) and (G) to (I)] or culture wells pooled from two to five mice per experiment [(C) to (E)]. qPCR gene expression is relative to *Actb*, represented as fold change to media control group per experiment [(C) to (E)]. Each graph indicates the mean ± SEM of replicates. ns, not significant; *P < 0.05; **P < 0.01; ***P < 0.001; ****P < 0.0001 using two-way ANOVA with Tukey’s correction (A), one-way ANOVA with Tukey’s correction [(D) and (E)], two-tailed unpaired *t* test (C), two-tailed Mann-Whitney test (G), two-tailed Welch’s *t* test (I), or simple linear regression (H).

Although *Calb2*^Δ*Il13ra1*^ cKO CGRP peptide concentrations were diminished in both gut compartments at steady state and at 7 d.p.i., CGRP concentrations were 2- to 2.5-fold greater in the muscularis compared with the LP (Fig. 5A). Furthermore, MMφ had the most *Calcrl* and *Ramp1* gene expression across immune cell clusters, with control MMφ sharing gene signatures with recently reported CGRP-treated macrophages (8), and *Calb2*^Δ*Il13ra1*^ cKO MMφ sharing signatures with gut macrophages from ILC2-deficient mice (25) (Fig. 5B and fig. S15, E to G). This suggests that CGRP may influence MMφ AAMφ programs. Indeed, both CGRPβ and activation of its downstream receptor signaling pathway were sufficient to induce *Arg1* expression in primary cultured SI macrophages (SI-Mφ) and bone marrow–derived macrophages (BMDMs) (Fig. 5, C and D, and fig. S15H). Moreover, CGRPβ synergized with lower doses of IL-4, amplifying BMDM *Arg1* expression to levels equivalent to those observed after treatment with 10× the dose of IL-4 alone (Fig. 5E and fig. S15I).

Consistently, daily CGRPβ and NMU23 in vivo co-administration completely restored *Calb2*^Δ*Il13ra1*^ cKO MMφ Arg1 expression deficits at 14 d.p.i. (Fig. 5, F and G; fig. S15J), and the percentage of muscularis Arg1^+^ immune cells was negatively correlated with *H. polygyrus* fecundity and burden (Fig. 5H; fig. S15K). Lastly, we infected mice globally deficient in *Ramp1* (*Ramp1* KO), finding that *Ramp1* KO mice had greater fecal egg burdens than heterozygous littermate controls at 14 d.p.i. (Fig. 5I), which suggests that CGRP receptor signaling is necessary for efficient *H. polygyrus* expulsion and works in synergy with IL-4 to promote AAMφ gene expression.

## Discussion

In this work, we uncovered the functional role of type 2 cytokine receptor signaling in PSNs during infection with the gastrointestinal helminth *H. polygyrus*. We demonstrated that direct PSN sensing of IL-4 and IL-13 is sufficient to induce robust expression of the immunomodulatory neuropeptides NMU and CGRPβ in vitro and in vivo, thereby completing a previously described neuropeptide-ILC2 neuroimmune feedback loop (1–3) (fig. S16, A to C). PSN-dependent helminth control is due in part to the action of NMU on ILC2s, because ILC2 NMUR1 expression is required for effective helminth control (2). Additionally, ILC2 CGRP receptor signaling limits proliferation while promoting IL-5 production (5, 16, 17) and muscularis IL-4^+^ eosinophil recruitment. Therefore, the highly correlated NMU and CGRPβ expression in PSNs may serve to promote appropriate muscularis-specific anti-helminth immunity while buffering the tissue-damaging effects of unrestrained type 2 inflammation in the muscularis. Furthermore, we showed that CGRPβ acts in synergy with IL-4 to induce macrophage Arg1 expression in vitro, and that PSN neuropeptide co-administration promotes Arg1 expression in MMφ, which are essential to *H. polygyrus* host defense and tissue protection (21, 23, 26). This work demonstrates how PSN cytokine sensing dictates this delicate neuropeptide balance and influences site-specific immunity.

## Supplementary Material

S1

S2_highres

S3_n2_no error bars copy_highres

S4_highres

S5_n2_no error bar

S6_highres

S7_n2-no-error-bar_highres

S8

S9_highres

S10

S11_high res

S12

S13_corrected 20250426

S14

S15

S16_highres

science.adn9850_data_s1

science.adn9850_data_s2

science.adn9850_data_s3

science.adn9850_data_s4

science.adn9850_data_s5

science.adn9850_mdar_reproducibility_checklist

science.adn9850_movie_s1

Supplementary_Materials_Methods_science.adn9850_sm

1

## Data Availability

All data needed to evaluate the conclusions in the paper are available in the main text or supplementary materials. Sequencing datasets are publicly available on Gene Expression Omnibus under accession numbers GSE292613 (scRNA-seq), GSE292633 (bulk RNA-seq, sorted ILC2), and GSE292634 (bulk RNA-seq, ENS cultures). Supporting data generated after publication will be available on Figshare (56).

## References

[1] Klose CSN , Nature 549, 282–286 (2017). 28869965 10.1038/nature23676 PMC6066372

[2] Cardoso V , Nature 549, 277–281 (2017). 28869974 10.1038/nature23469 PMC5714273

[3] Wallrapp A , Nature 549, 351–356 (2017). 28902842 10.1038/nature24029 PMC5746044

[4] Talbot J , Nature 579, 575–580 (2020). 32050257 10.1038/s41586-020-2039-9 PMC7135938

[5] Wallrapp A , Immunity 51, 709–723.e6 (2019). 31604686 10.1016/j.immuni.2019.09.005 PMC7076585

[6] Hanč P , Science 379, eabm5658 (2023). 36996219 10.1126/science.abm5658 PMC10642951

[7] Balood M , Nature 611, 405–412 (2022). 36323780 10.1038/s41586-022-05374-w PMC9646485

[8] Pinho-Ribeiro FA , Nature 615, 472–481 (2023). 36859544 10.1038/s41586-023-05753-x PMC10593113

[9] Maizels RM , Exp. Parasitol. 132, 76–89 (2012). 21875581 10.1016/j.exppara.2011.08.011 PMC6485391

[10] Drokhlyansky E , Cell 182, 1606–1622.e23 (2020). 32888429 10.1016/j.cell.2020.08.003 PMC8358727

[11] Morarach K , Nat. Neurosci. 24, 34–46 (2021). 33288908 10.1038/s41593-020-00736-x PMC7610403

[12] Zeisel A , Cell 174, 999–1014.e22 (2018). 30096314 10.1016/j.cell.2018.06.021 PMC6086934

[13] Hibberd TJ , J. Comp. Neurol. 530, 3209–3225 (2022). 36043843 10.1002/cne.25403

[14] Scott MM, Williams KW, Rossi J, Lee CE, Elmquist JK, J. Clin. Invest. 121, 2413–2421 (2011). 21606595 10.1172/JCI43703 PMC3104740

[15] Nussbaum JC , Nature 502, 245–248 (2013). 24037376 10.1038/nature12526 PMC3795960

[16] Xu H , Immunity 51, 696–708.e9 (2019). 31618654 10.1016/j.immuni.2019.09.004 PMC6991097

[17] Nagashima H , Immunity 51, 682–695.e6 (2019). 31353223 10.1016/j.immuni.2019.06.009 PMC6801073

[18] Huang Y , Science 359, 114–119 (2018). 29302015 10.1126/science.aam5809 PMC6956613

[19] Ahrends T , Cell 184, 5715–5727.e12 (2021). 34717799 10.1016/j.cell.2021.10.004 PMC8595755

[20] Hewitson JP , PLOS Pathog. 11, e1004676 (2015). 25816012 10.1371/journal.ppat.1004676 PMC4376884

[21] Esser-von Bieren J , PLOS Pathog. 9, e1003771 (2013). 24244174 10.1371/journal.ppat.1003771 PMC3828184

[22] Chen F , Cell Rep. 38, 110215 (2022). 35021079 10.1016/j.celrep.2021.110215 PMC9403845

[23] Esser-von Bieren J , J. Immunol. 194, 1154–1163 (2015). 25548226 10.4049/jimmunol.1401645 PMC4298127

[24] Coakley G, Harris NL, Parasite Immunol. 42, e12717 (2020). 32249432 10.1111/pim.12717

[25] Jarick KJ , Nature 611, 794–800 (2022). 36323785 10.1038/s41586-022-05395-5 PMC7614745

[26] Anthony RM , Nat. Med. 12, 955–960 (2006). 16892038 10.1038/nm1451 PMC1955764

[27] Copeland NG, Jenkins NA, Court DL, Nat. Rev. Genet. 2, 769–779 (2001). 11584293 10.1038/35093556

[28] Warming S, Costantino N, Court DL, Jenkins NA, Copeland NG, Nucleic Acids Res. 33, e36 (2005). 15731329 10.1093/nar/gni035 PMC549575

[29] Perner C, Sokol CL, STAR Protoc. 2, 100333 (2021). 33615276 10.1016/j.xpro.2021.100333 PMC7876630

[30] Toda G, Yamauchi T, Kadowaki T, Ueki K, STAR Protoc. 2, 100246 (2020). 33458708 10.1016/j.xpro.2020.100246 PMC7797923

[31] Johnston CJC , J. Vis. Exp. 98, e52412 (2015). 10.3791/52412 PMC4401400 25867600

[32] Finkelman FD , J. Immunol. 151, 1235–1244 (1993). 8393043

[33] Zhu X , Proc. Natl. Acad. Sci. U.S.A. 116, 11480–11489 (2019). 31101714 10.1073/pnas.1819583116 PMC6561250

[34] Li W, Germain RN, Gerner MY, Nat. Protoc. 14, 1708–1733 (2019). 31028373 10.1038/s41596-019-0156-4 PMC8690297

[35] Häring M, Fatt M, Kupari J, STAR Protoc. 1, 100030 (2020). 33111081 10.1016/j.xpro.2020.100030 PMC7580114

[36] Esterházy D , Nature 569, 126–130 (2019). 30988509 10.1038/s41586-019-1125-3 PMC6587593

[37] Chen C-C, Louie S, McCormick B, Walker WA, Shi HN, Infect. Immun. 73, 5468–5481 (2005). 16113263 10.1128/IAI.73.9.5468-5481.2005 PMC1231118

[38] Picelli S , Nat. Protoc. 9, 171–181 (2014). 24385147 10.1038/nprot.2014.006

[39] Li B , Nat. Methods 17, 793–798 (2020). 32719530 10.1038/s41592-020-0905-x PMC7437817

[40] Dobin A , Bioinformatics 29, 15–21 (2013). 23104886 10.1093/bioinformatics/bts635 PMC3530905

[41] Li B, Dewey CN, BMC Bioinformatics 12, 323 (2011). 21816040 10.1186/1471-2105-12-323 PMC3163565

[42] Love MI, Huber W, Anders S, Genome Biol. 15, 550 (2014). 25516281 10.1186/s13059-014-0550-8 PMC4302049

[43] Wolf FA, Angerer P, Theis FJ, Genome Biol. 19, 15 (2018). 29409532 10.1186/s13059-017-1382-0 PMC5802054

[44] Wolock SL, Lopez R, Klein AM, Cell Syst. 8, 281–291.e9 (2019). 30954476 10.1016/j.cels.2018.11.005 PMC6625319

[45] Germain P-L, Lun A, Garcia Meixide C, Macnair W, Robinson MD, F1000 Res. 10, 979 (2021). 10.12688/f1000research.73600.1 PMC9204188 35814628

[46] McInnes L, Healy J, Melville J, UMAP: Uniform Manifold Approximation and Projection for Dimension Reduction. arXiv:1802.03426 [stat.ML] (2018).

[47] Traag VA, Waltman L, van Eck NJ, Sci. Rep. 9, 5233 (2019). 30914743 10.1038/s41598-019-41695-z PMC6435756

[48] Domínguez Conde C , Science 376, eabl5197 (2022). 35549406 10.1126/science.abl5197 PMC7612735

[49] Xu C , Cell 186, 5876–5891.e20 (2023). 38134877 10.1016/j.cell.2023.11.026

[50] Tirosh I , Science 352, 189–196 (2016). 27124452 10.1126/science.aad0501 PMC4944528

[51] Hao Y , Nat. Biotechnol. 42, 293–304 (2024). 37231261 10.1038/s41587-023-01767-y PMC10928517

[52] Korotkevich G , Fast gene set enrichment analysis, bioRxiv 060012 [Preprint] (2016); 10.1101/060012.

[53] Hafemeister C, Satija R, Genome Biol. 20, 296 (2019). 31870423 10.1186/s13059-019-1874-1 PMC6927181

[54] Ahlmann-Eltze C, Huber W, Bioinformatics 36, 5701–5702 (2021). 33295604 10.1093/bioinformatics/btaa1009 PMC8023675

[55] Stuart T , Cell 177, 1888–1902.e21 (2019). 31178118 10.1016/j.cell.2019.05.031 PMC6687398

[56] Data for: Barilla R , Type 2 cytokines act on enteric sensory neurons to regulate neuropeptide-driven host defense, Figshare (2025); 10.6084/m9.figshare.c.7716548. PMC12632183 40403128

