# Supplementary figures and images for: Type 2 cytokines act on enteric sensory neurons to regulate neuropeptide-driven host defense
Source: Science. Author manuscript; Available in PMC 2025 Nov 20. (PMC12632183; doi:10.1126/science.adn9850)

Supplemental fig. S2

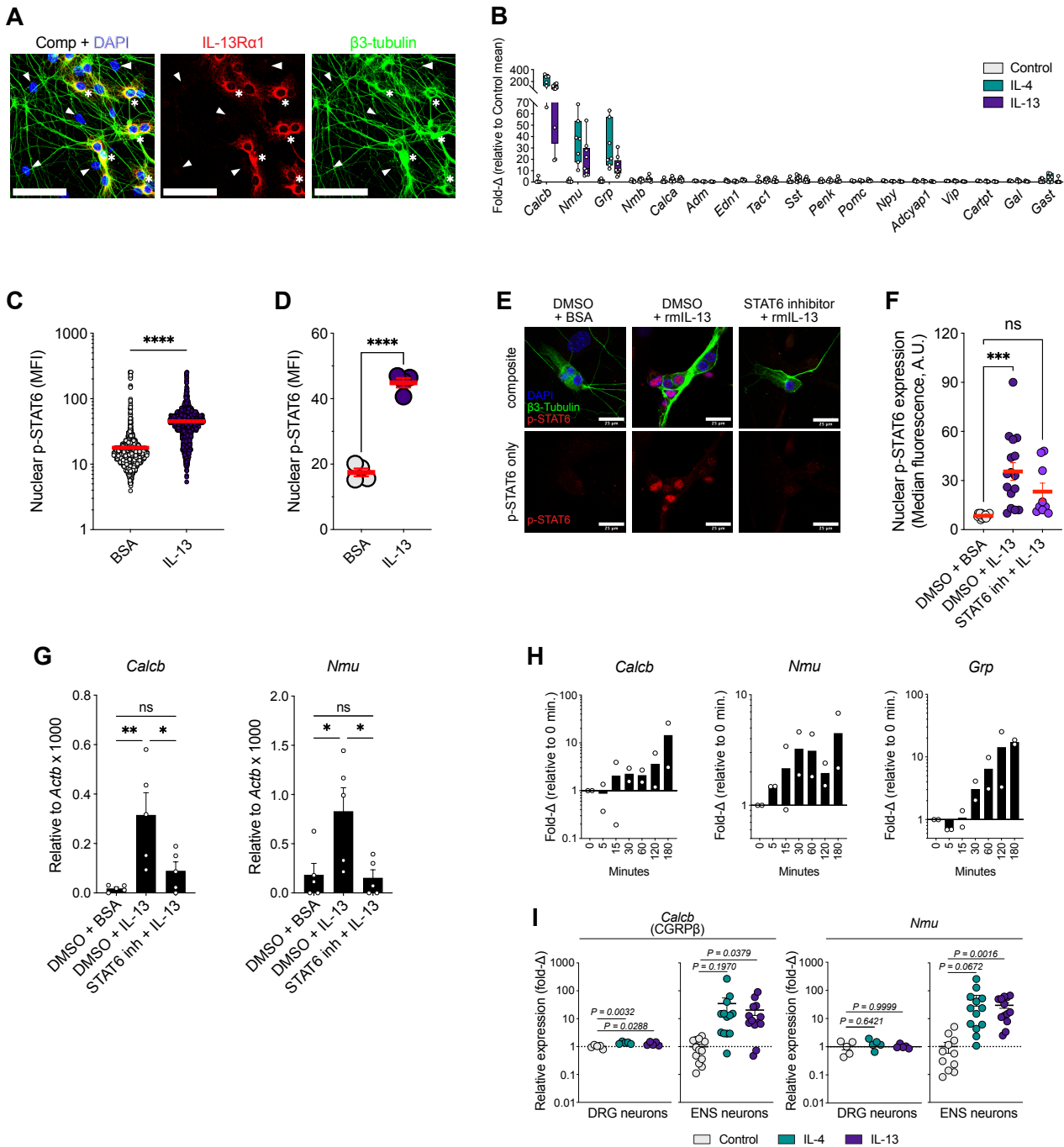

Supplement: S2_highres [file NIHMS2112150-supplement-S2_highres.pdf]

Supplemental fig. S3

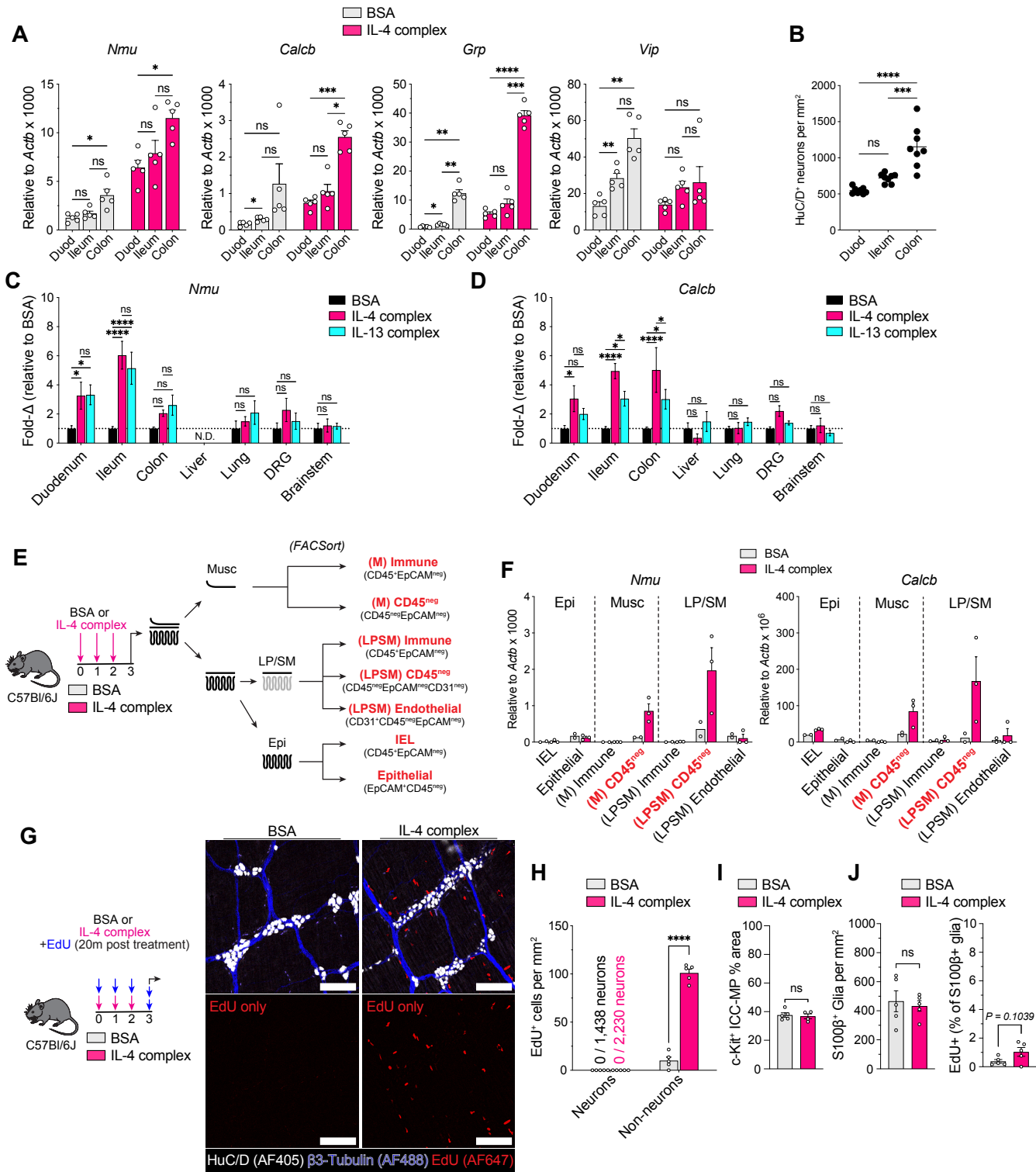

Supplement: S3_n2_no error bars copy_highres [file NIHMS2112150-supplement-S3_n2_no_error_bars_copy_highres.pdf]

Supplemental fig. S4

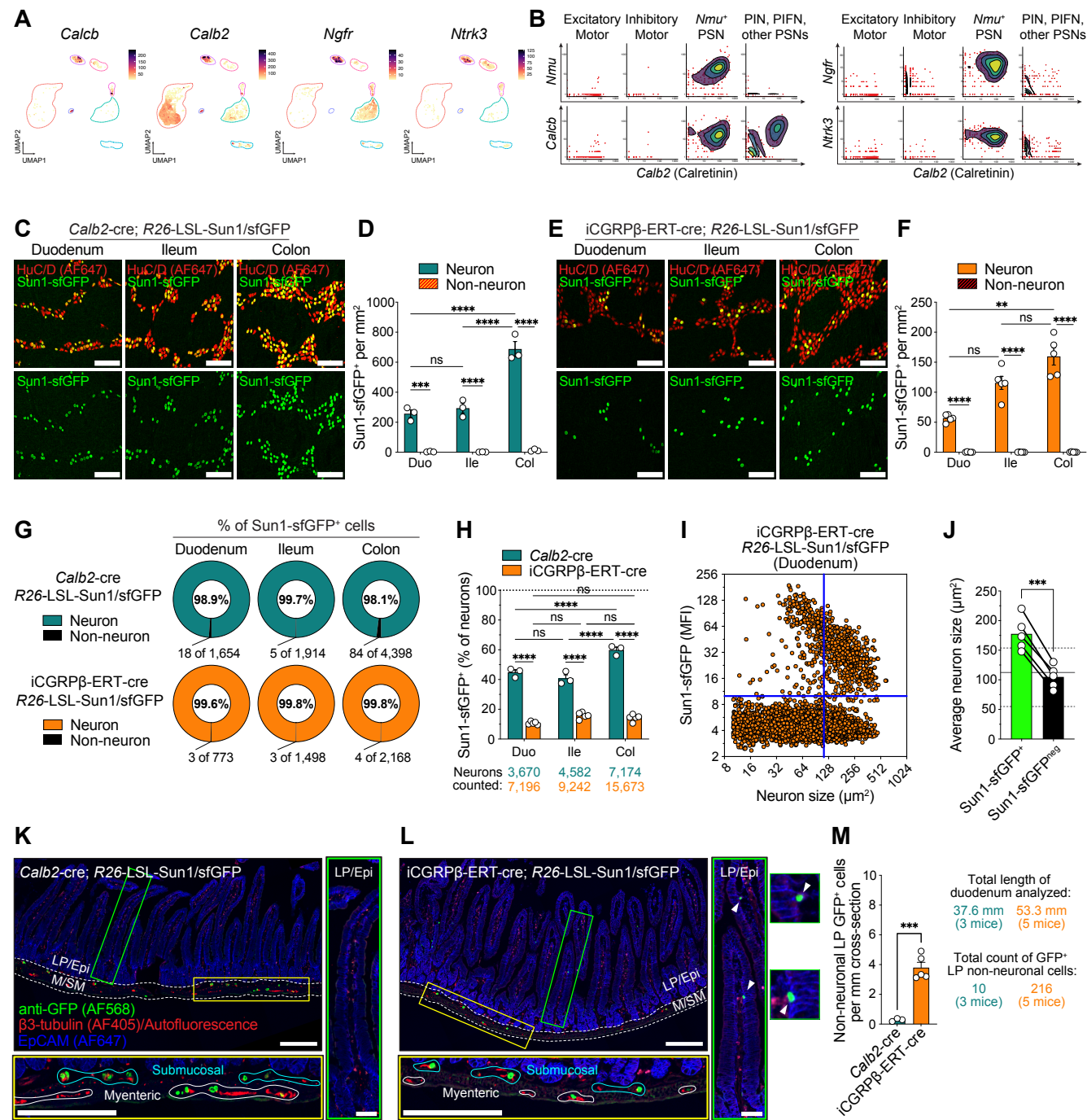

Supplement: S4_highres [file NIHMS2112150-supplement-S4_highres.pdf]

# Supplemental fig. S5

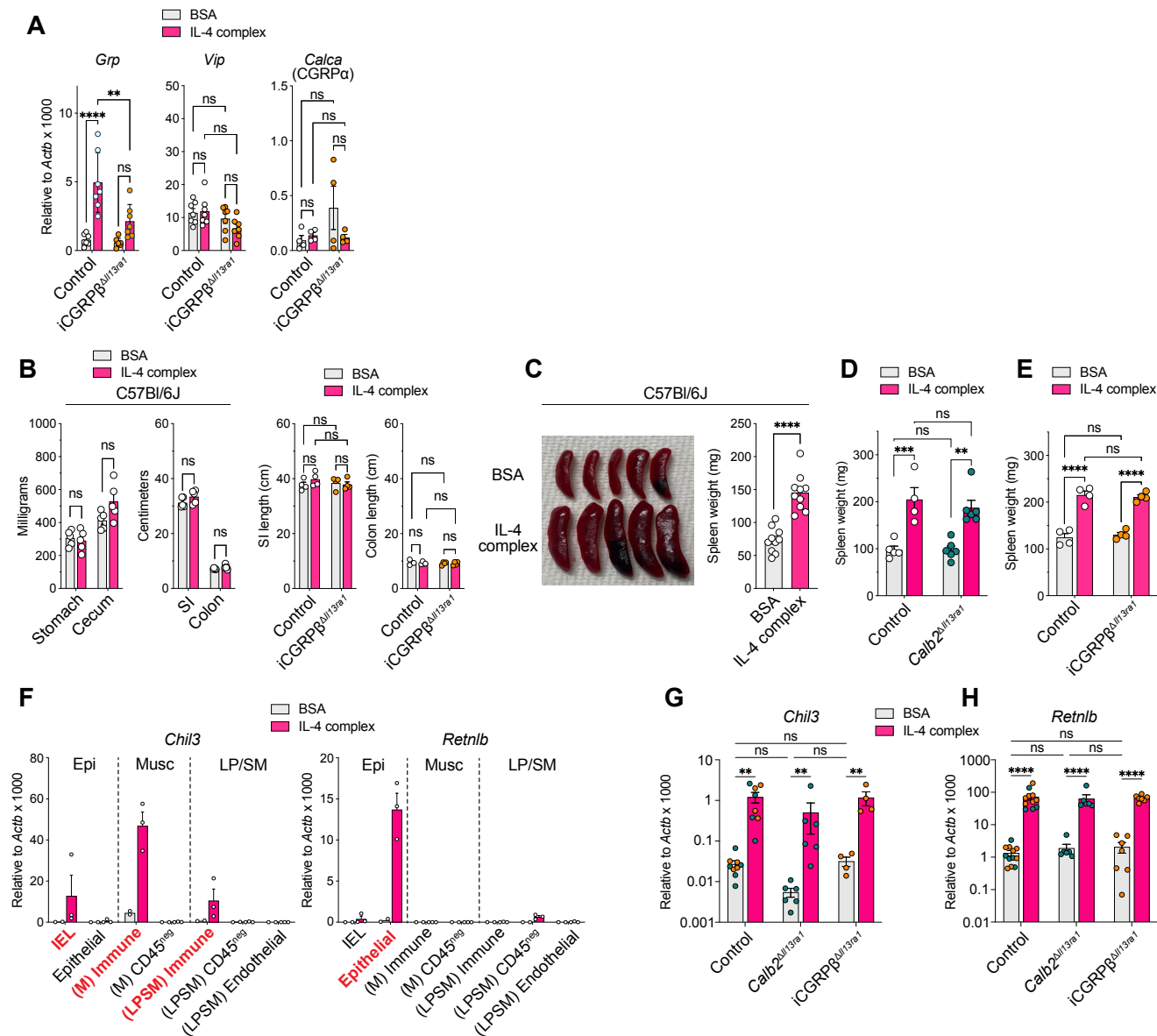

Supplement: S5_n2_no error bar [file NIHMS2112150-supplement-S5_n2_no_error_bar.pdf]

Supplemental fig. S6

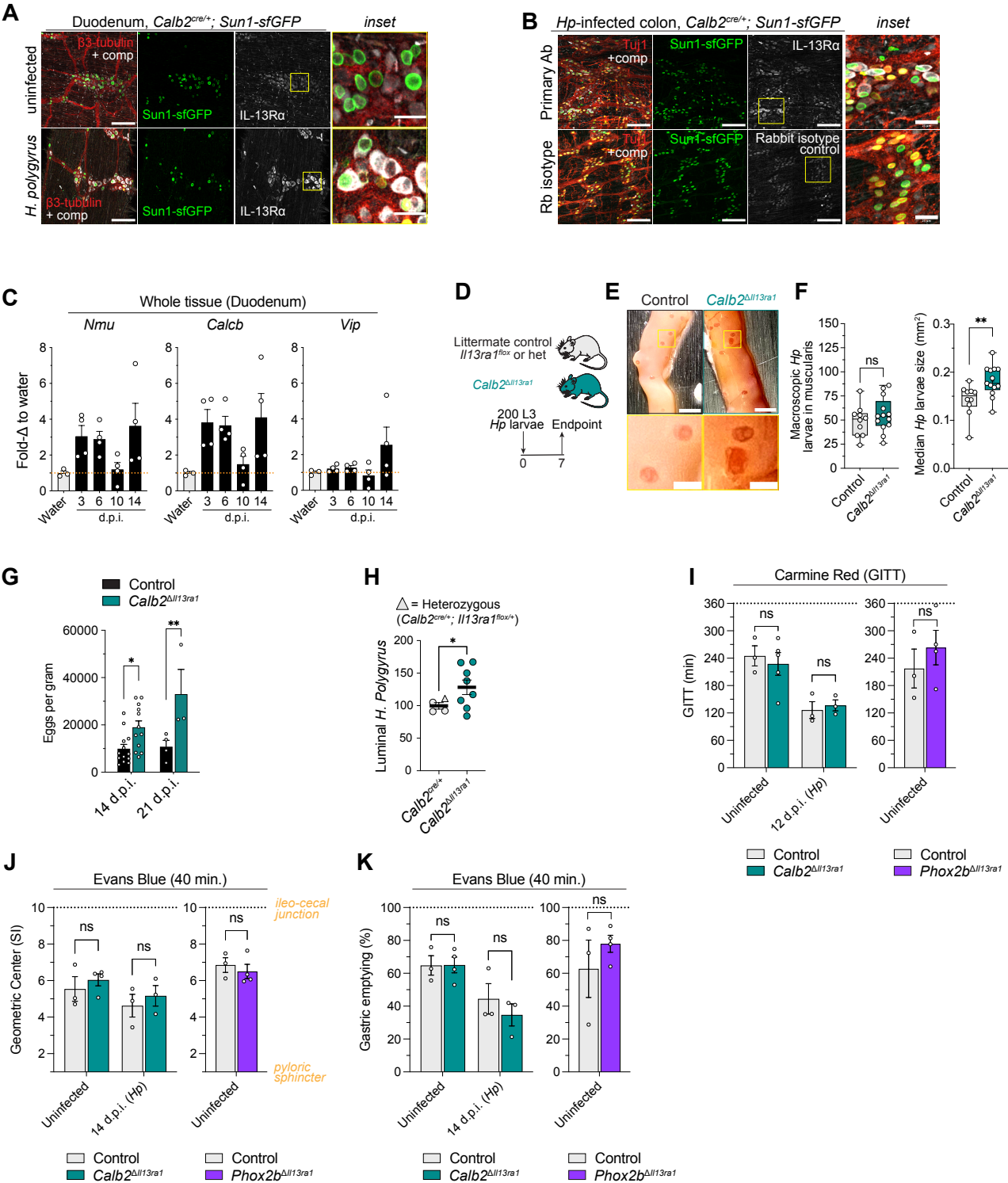

Supplement: S6_highres [file NIHMS2112150-supplement-S6_highres.pdf]

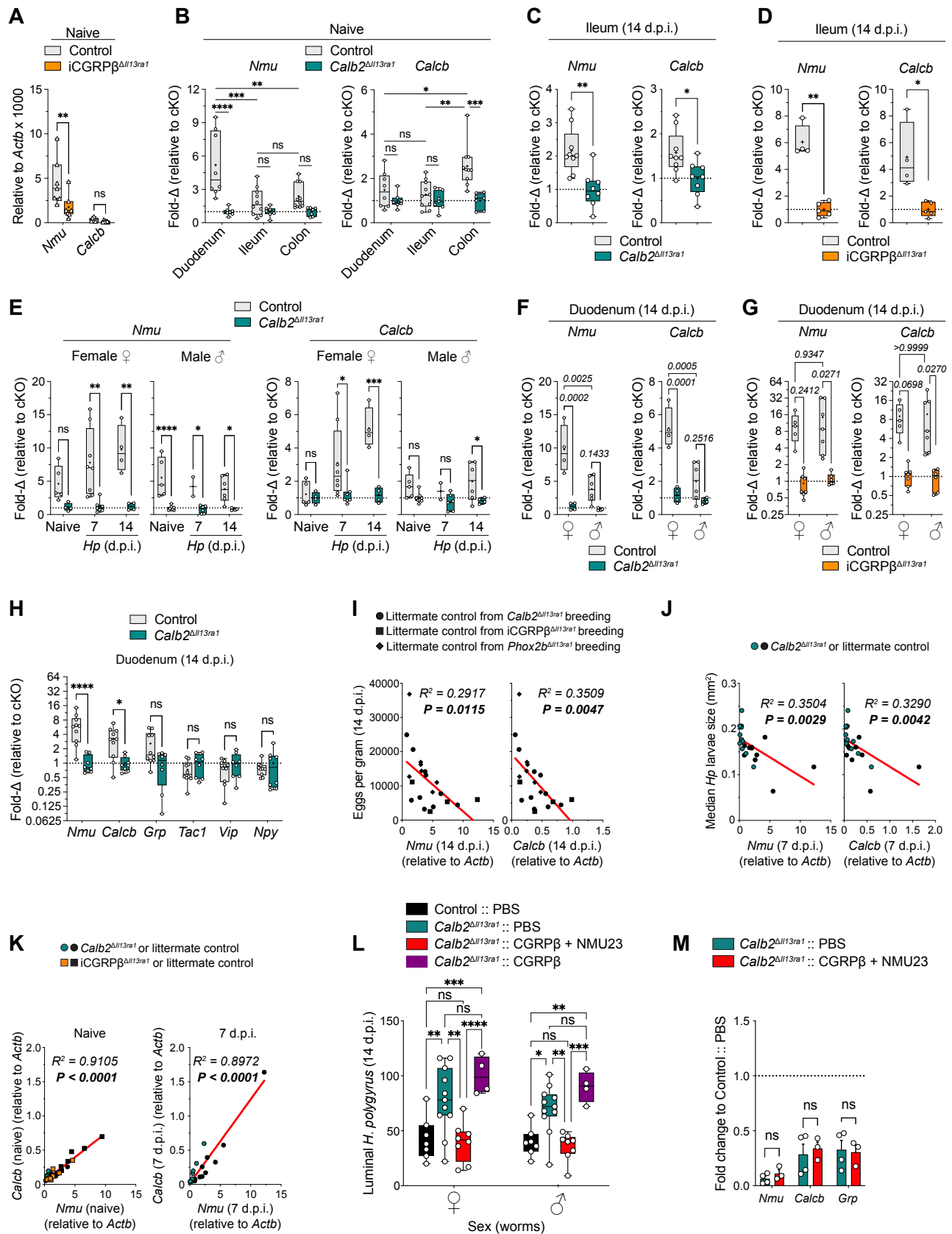

Supplement: S8 [file NIHMS2112150-supplement-S8.pdf]

Supplemental fig. S9

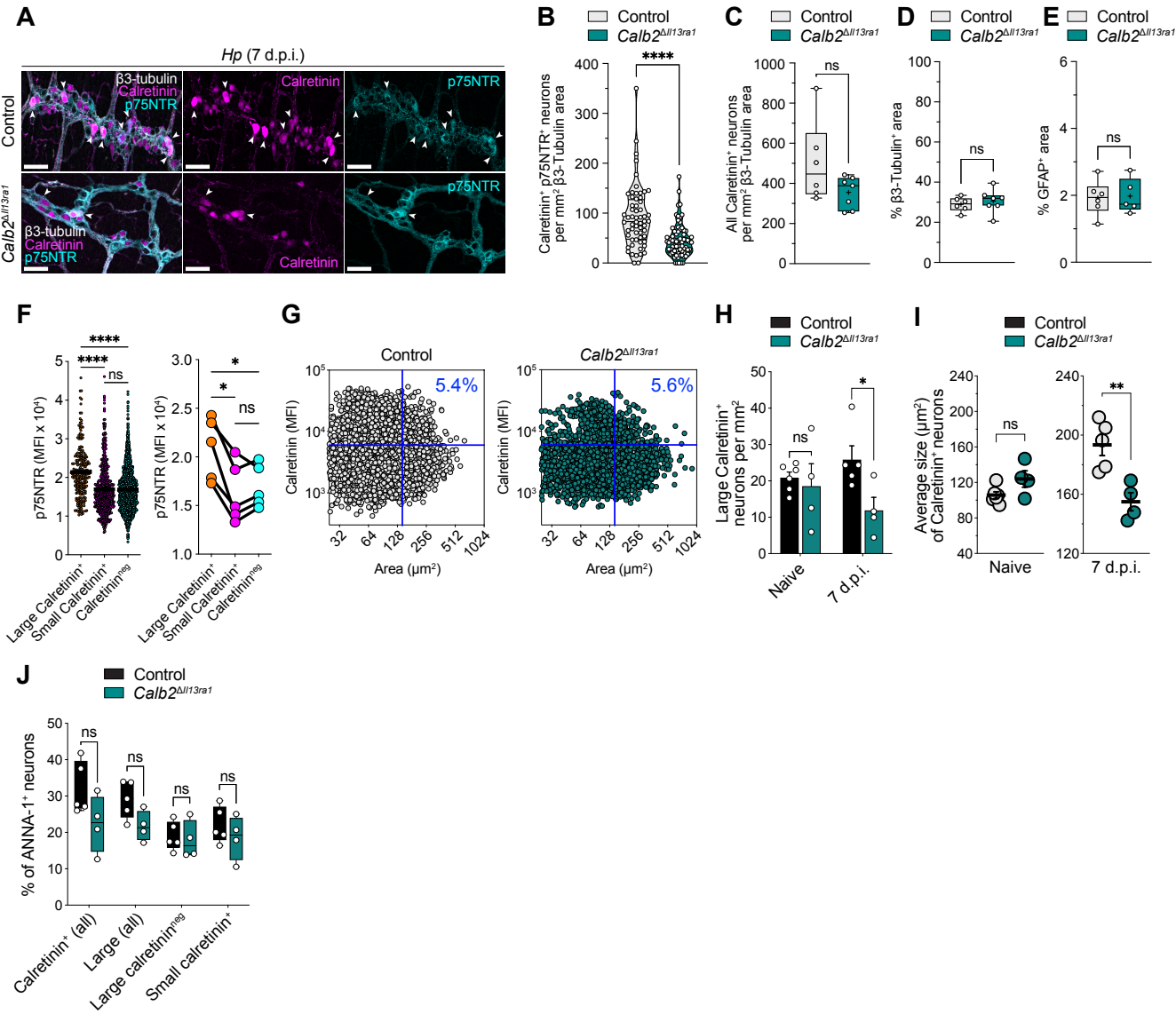

Supplement: S9_highres [file NIHMS2112150-supplement-S9_highres.pdf]

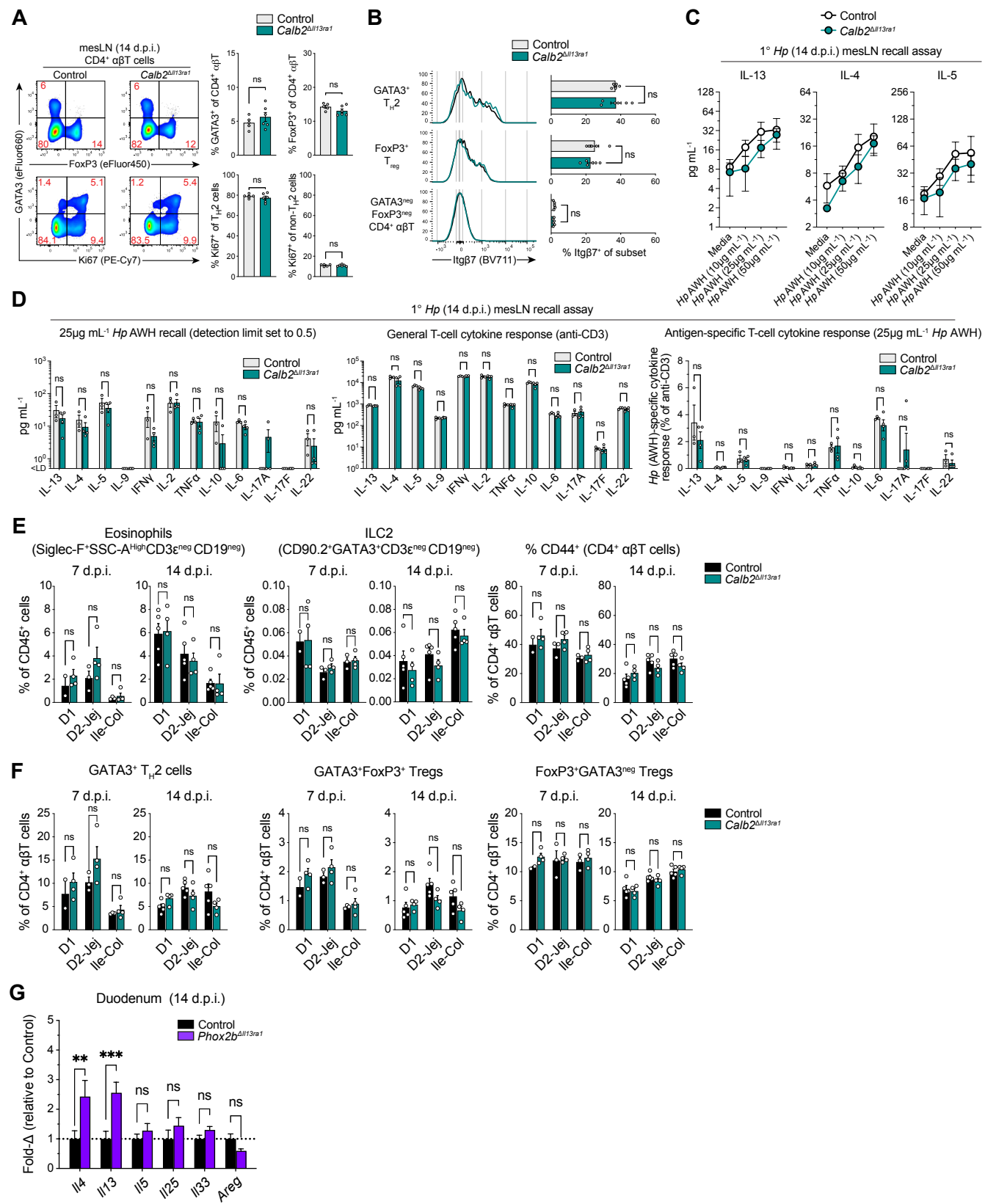

Supplement: S10 [file NIHMS2112150-supplement-S10.pdf]

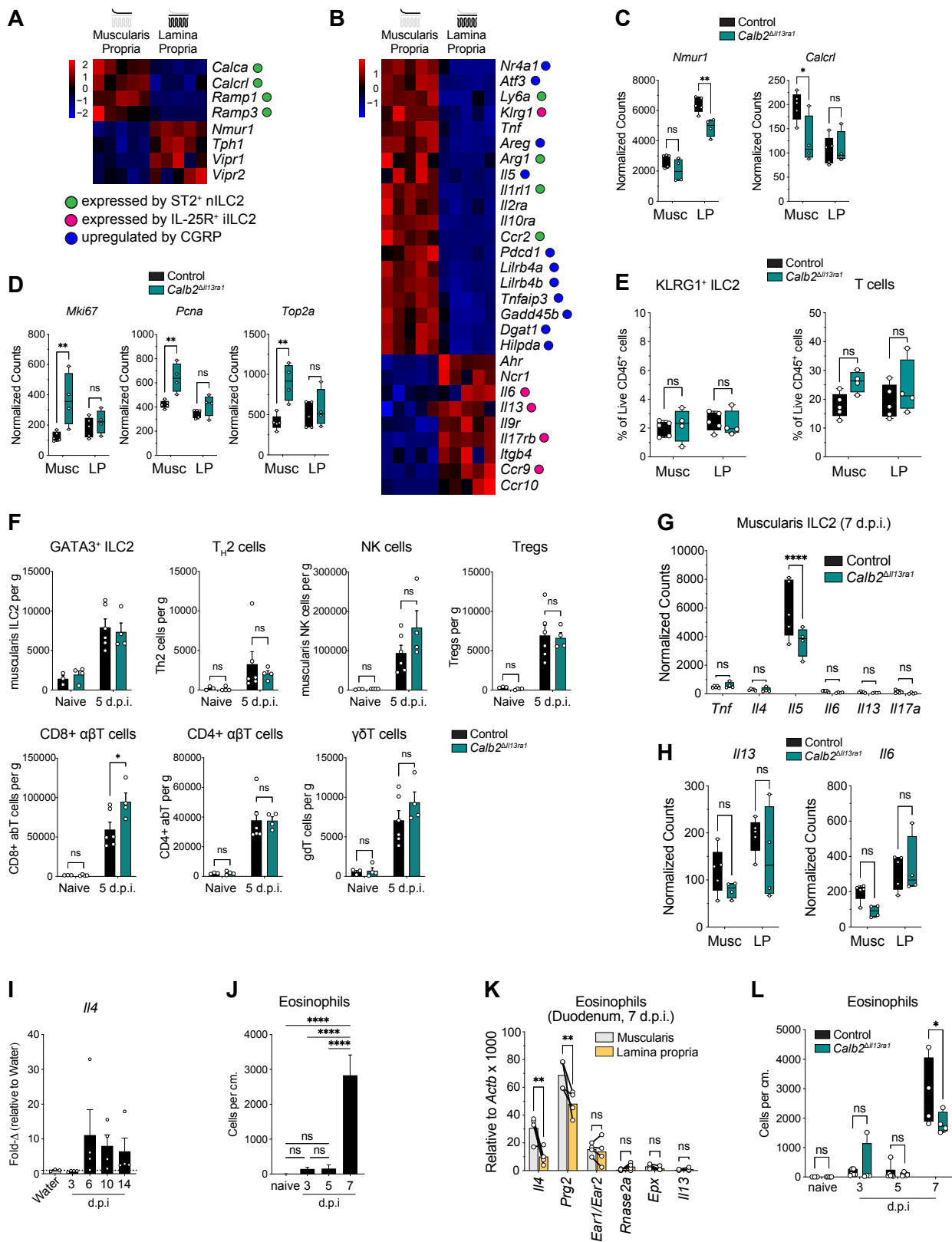

Supplement: S12 [file NIHMS2112150-supplement-S12.pdf]

# Supplemental fig. S13

**A**

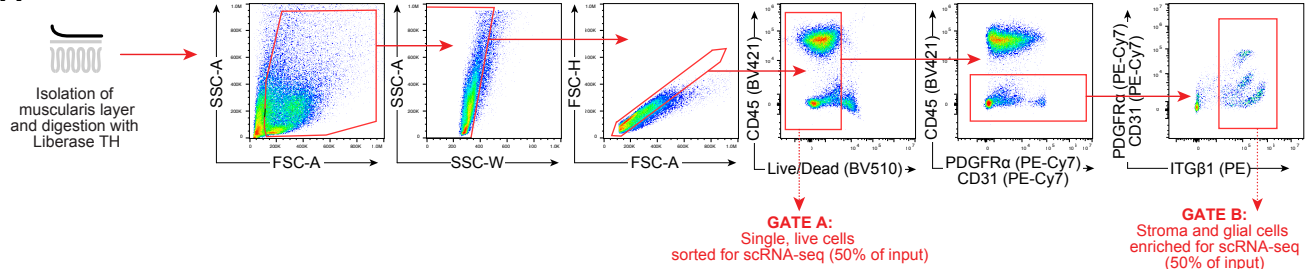

**B**

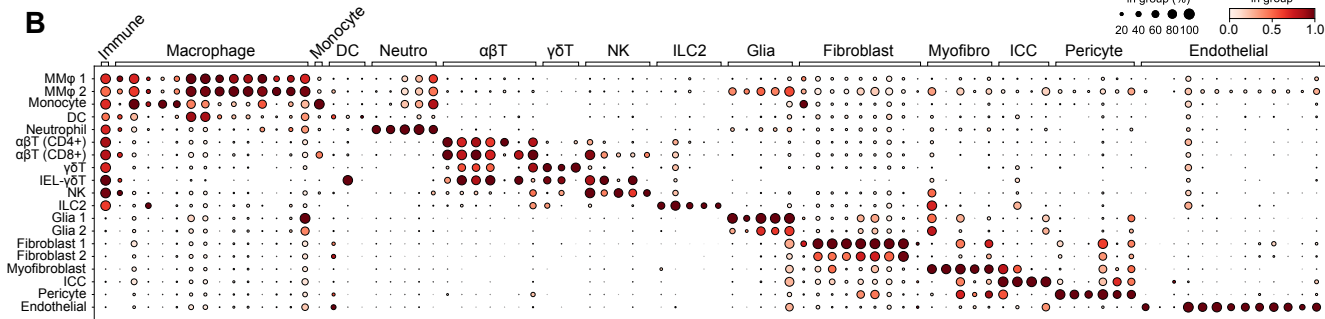

**C**

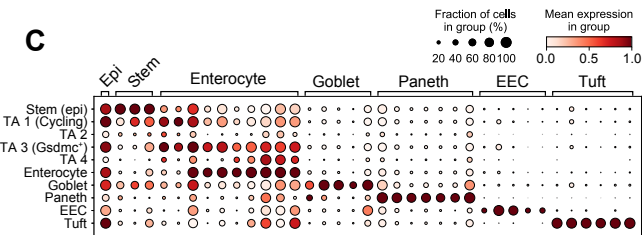

**D**

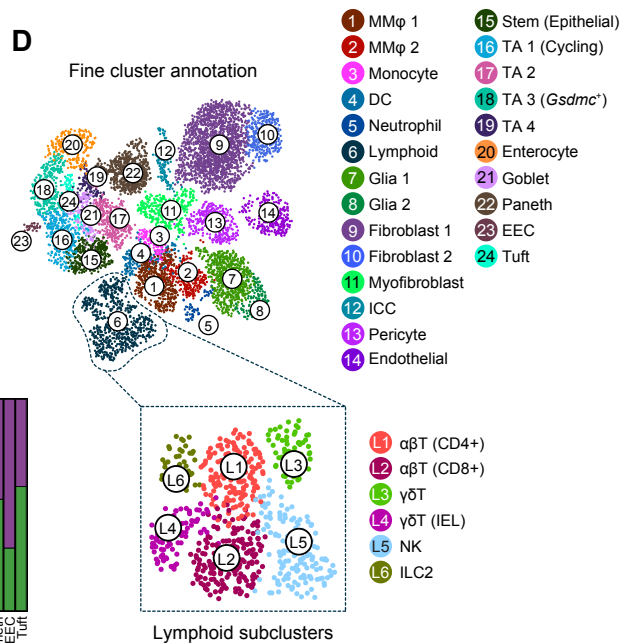

**E**

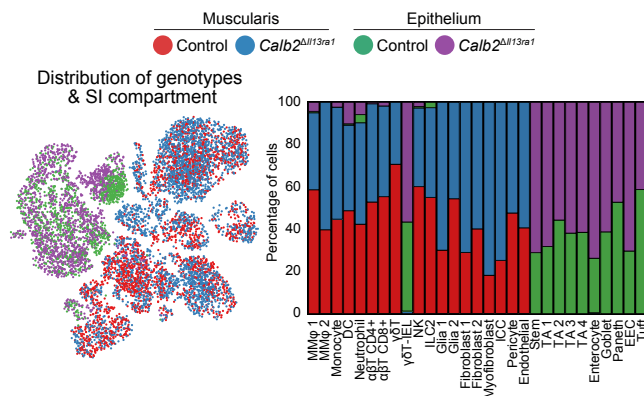

**F**

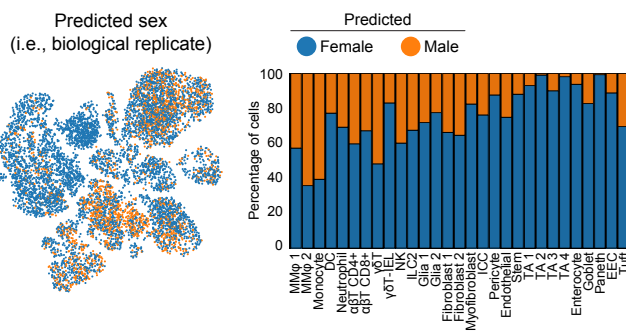

Supplement: S13_corrected 20250426 [file NIHMS2112150-supplement-S13_corrected_20250426.pdf]

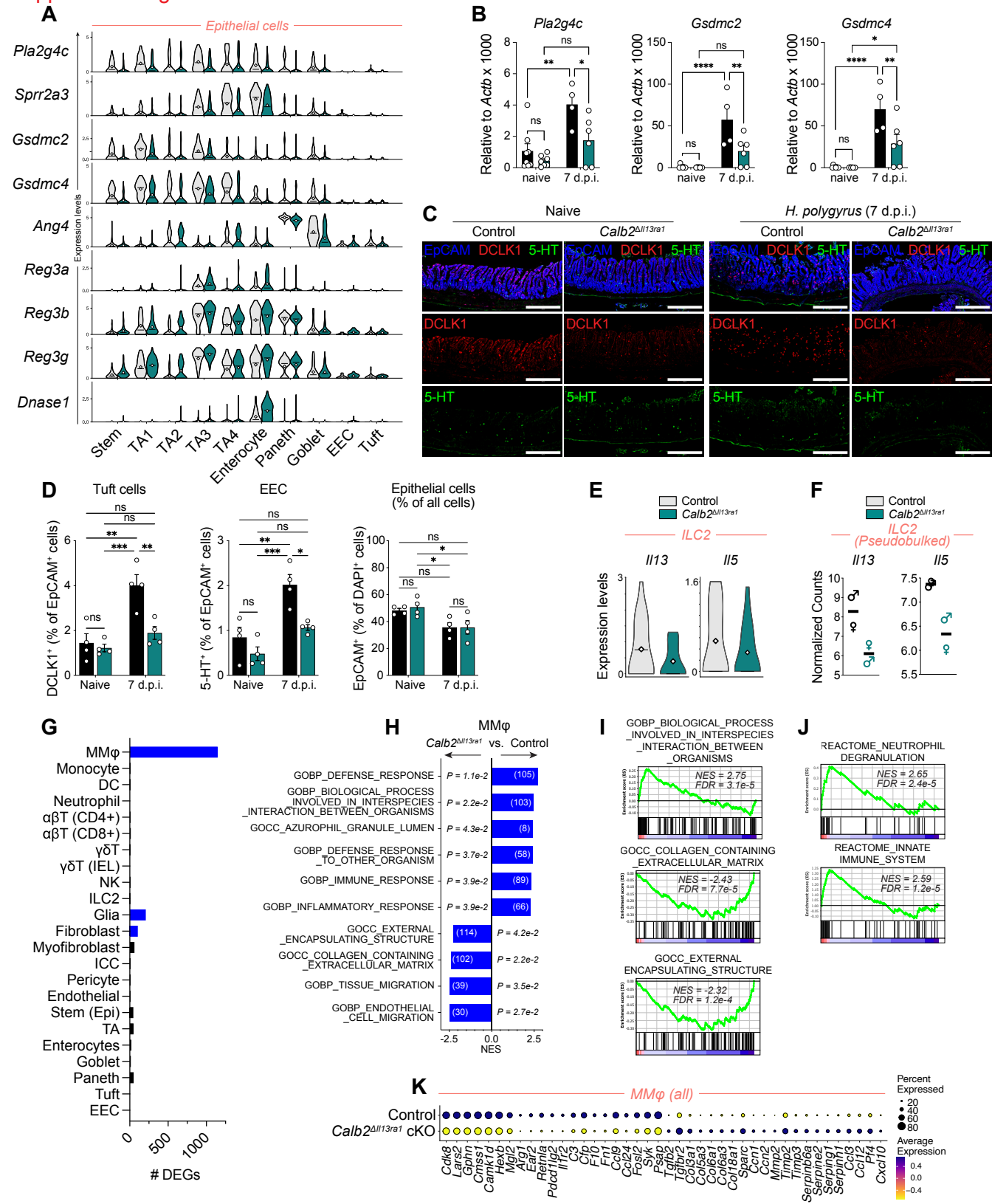

Supplement: S14 [file NIHMS2112150-supplement-S14.pdf]

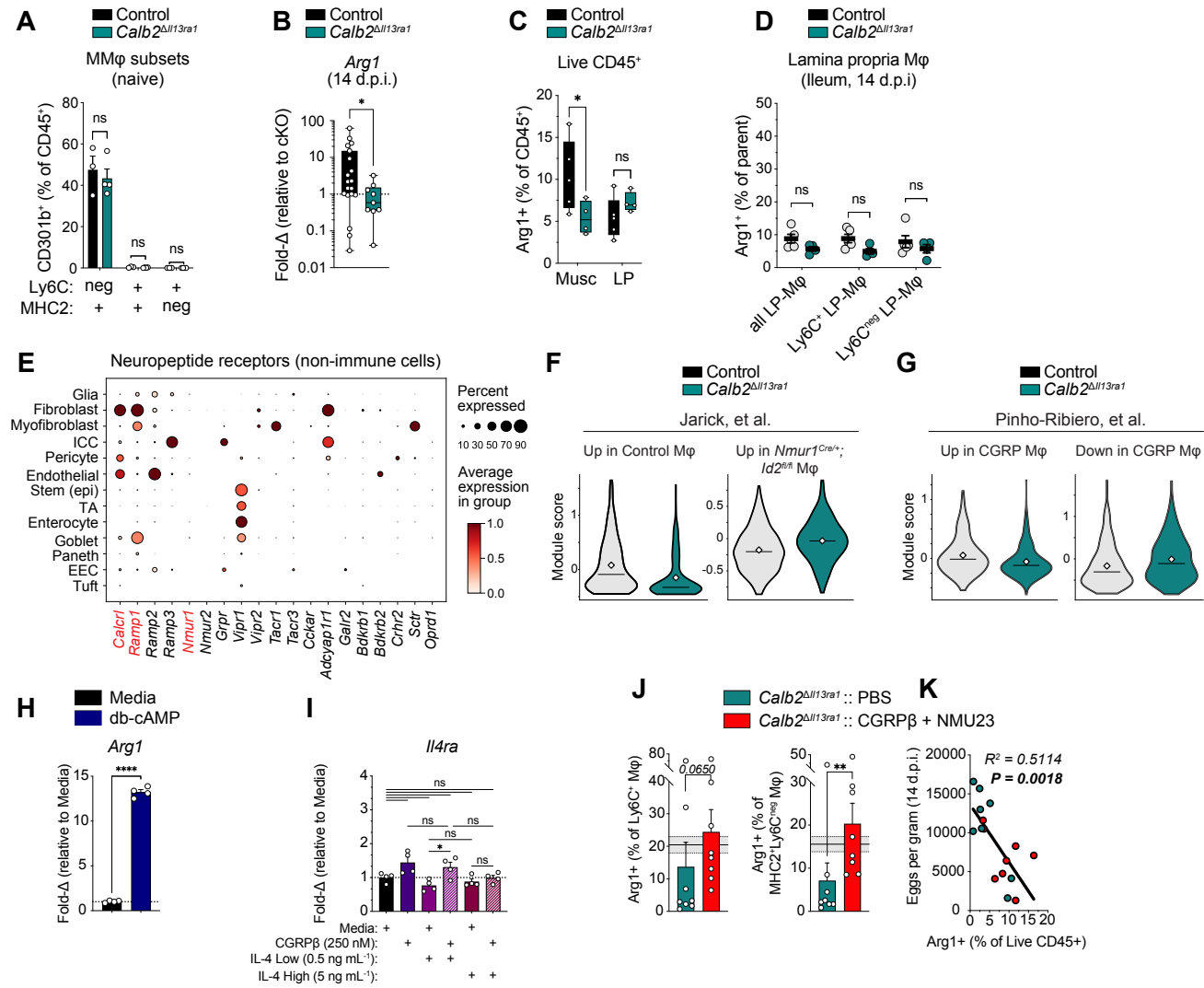

Supplement: S15 [file NIHMS2112150-supplement-S15.pdf]

Supplemental fig. S16

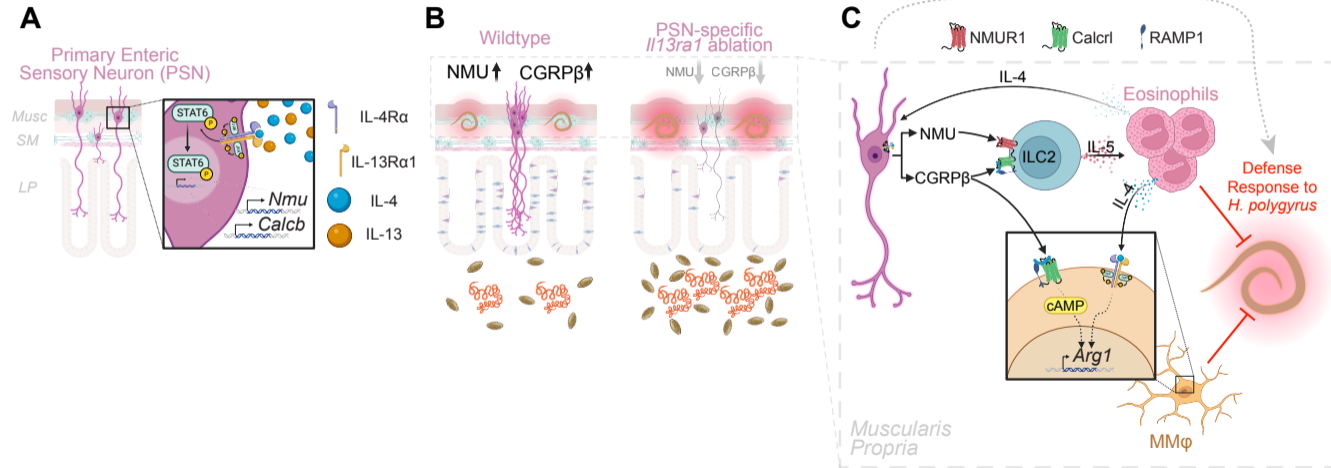

Supplement: S16_highres [file NIHMS2112150-supplement-S16_highres.pdf]
