# Supplementary material for: Type 2 cytokines act on enteric sensory neurons to regulate neuropeptide-driven host defense
Source: Science. Author manuscript; Available in PMC 2025 Nov 20. (PMC12632183; doi:10.1126/science.adn9850)

Supplemental fig. S11

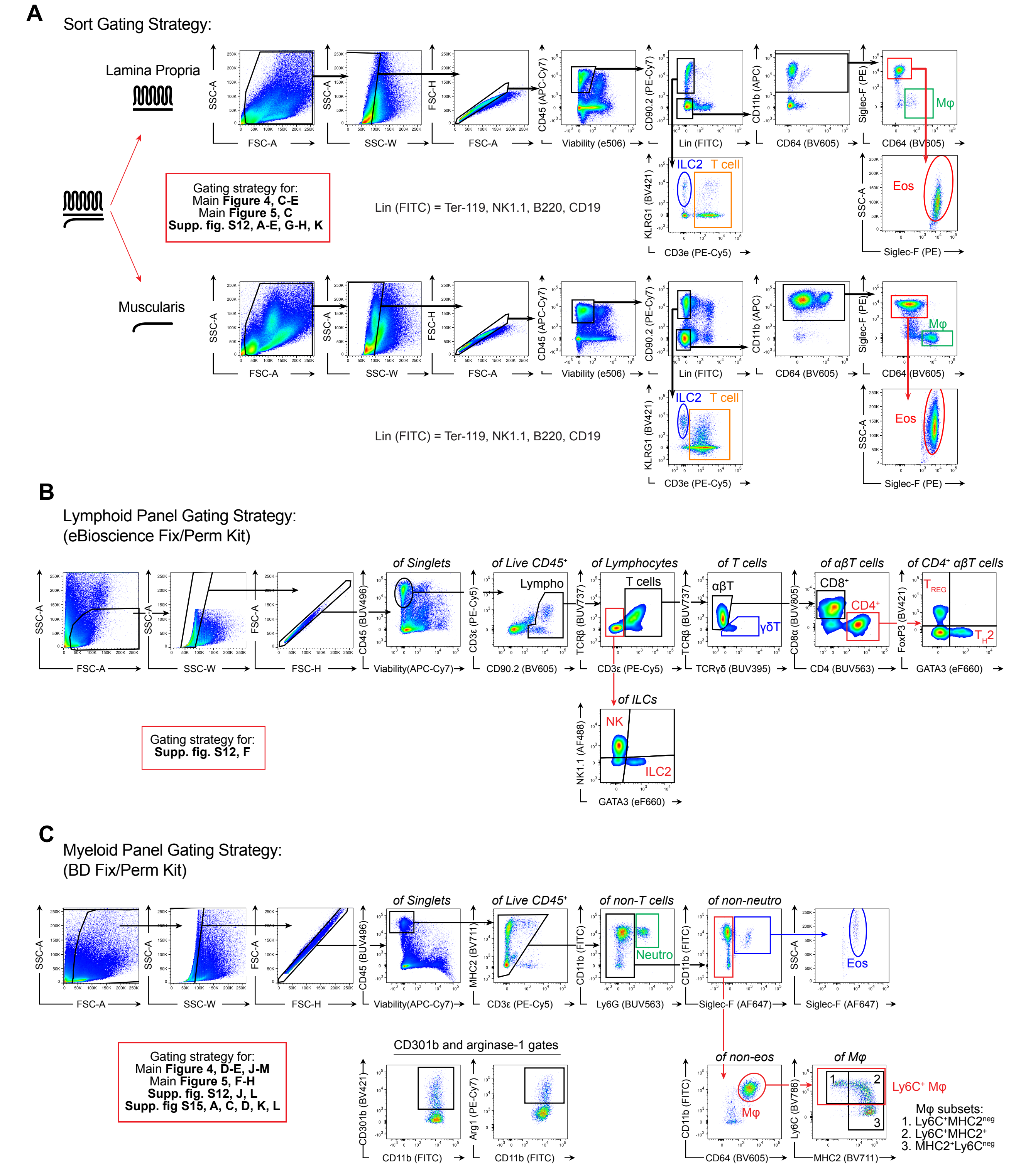

Supplement: S11_high res [file NIHMS2112150-supplement-S11_high_res.pdf]
