# Supplementary material for: Type 2 cytokines act on enteric sensory neurons to regulate neuropeptide-driven host defense
Source: Science. Author manuscript; Available in PMC 2025 Nov 20. (PMC12632183; doi:10.1126/science.adn9850)
Supplement: Supplementary_Materials_Methods_science.adn9850_sm [file NIHMS2112150-supplement-Supplementary_Materials_Methods_science_adn9850_sm.pdf]

Science
AAAS

Supplementary Materials for

Type 2 cytokines act on enteric sensory neurons to regulate neuropeptide-driven host defense

Rocky M. Barilla *et al*.

Corresponding authors: Ramnik J. Xavier, xavier@molbio.mgh.harvard.edu; Vijay K. Kuchroo, vkuchroo@ rics.bwh.harvard.edu

Science **389**, 260 (2025)
DOI: 10.1126/science.adn9850

The PDF file includes:

Materials and Methods
Figs. S1 to S16
References

Other Supplementary Material for this manuscript includes the following:

MDAR Reproducibility Checklist
Table S1
Data S1 to S5
Movie S1

Materials and Methods:

Mice

All experiments involving mice were approved by the Institutional Animal Care and Use Committee (IACUC) at Brigham and Women's Hospital (2016N000444). Mice were maintained in the animal facility at Brigham and Women's Hospital under specific pathogen-free conditions with food and water *ad libitum* and a 12 h dark/light cycle. Experiments were performed on mice between 2-6 months old and were either co-housed and littermate-controlled (e.g., *H. polygyrus* experiments) or sex-matched, age-matched, and randomized (e.g., IL-4 complex experiments). C57BL/6J (stock #000664), BALB/cJ (stock #000651), *Calb2*-cre (B6(Cg)-*Calb2*$^{tm1(cre)Zjh}$/J, stock #010774), *Phox2b*-cre (B6(Cg)-Tg(*Phox2b-cre*)3Jke/J, stock #016223), *Sun1-sfGFP* ([B6;129-*Gt(ROSA)26Sor*$^{tm5(CAG\text{-}Sun1/sfGFP)Nat}$/J, stock #021039] and [B6.129-*Gt(ROSA)26Sor*$^{tm5.1(CAG\text{-}Sun1/sfGFP)Nat}$/MmbeJ, stock #030952]) mice were purchased from The Jackson Laboratories (JAX) and bred in-house. *Calb2*$^{\Delta Il13ra1}$, iCGRPβ$^{\Delta Il13ra1}$, *Phox2b*$^{\Delta Il13ra1}$, *Calb2-cre; Sun1-sfGFP*, *Phox2b-cre; Sun1-sfGFP,* and littermate control mice were crossed in-house and bred and maintained on the mixed backgrounds of the parental mouse strains initially purchased or gifted. *Ramp1*$^{-/-}$ mice (Tsujikawa et al., 2007) were provided by Wade Kingery (Veterans Affairs Palo Alto Health Care System) with permission by Kazutake Tsujikawa (Osaka University, Osaka).

On rare occasion, *Calb2*-cre mice crossed to *Il13ra1*-floxed mice yielded germline mutations in the pups. However, mice with these mutations were detected upon genotyping and excluded from analyses.

Generation of Il13ra1-floxed mice—*Il13ra1*^{flox} mice were generated and kindly provided by Habib Zaghouani (University of Missouri) at the MU Animal Modeling Core. All related procedures were approved by the IACUC at the University of Missouri. Briefly, chimeric mice were generated by injecting targeted *Il13ra1*$^{tm1a(EUCOMM)Hmgu}$ embryonic stem (ES) cells (purchased from European Mouse Mutant Cell Repository (EuMMCR), MGI: 4455834) into B6(Cg)-*Tyr*$^{c\text{-}2J}$/J blastocysts. Embryos were surgically transferred to pseudo pregnant surrogate CD-1 females the same day as blastocyst injections, and chimeric offspring were genotyped and subsequently bred to B6(Cg)-*Tyr*$^{c\text{-}2J}$/J females to assess germline transmission. Male offspring carrying the *Il13ra1* mutation were crossed into Flp-deleter mice to remove the *frt*-LacZ-Neomycin cassette, thus

creating conditional-ready floxed animals with *loxP* sites flanking exon 4 of the mouse *Il13ra1* gene. B6(Cg)-*Tyr*$^{c-2J}$/J embryo donor females (3 weeks of age), stud males (10 weeks of age) and breeder females used to assess germline transmission (8 weeks of age) were purchased from Jackson Laboratory. CD-1 surrogate females (8 weeks of age) were purchased from Charles River.

<u>Generation of iCGRPβ-ERT-cre mice</u>—iCGRPβ-ERT-cre (*Calcb*$^{ERT\text{-}cre}$ or *Calcb*^{creER}) mice were generated and kindly provided by Meenakshi Rao (Boston Children's Hospital) and Michael L. Rutlin (Boston Children's Hospital). Briefly, a targeting vector was made using a 2-step recombineering protocol (*27*, *28*). The genomic sequence of mouse *Calcb* (strain C57BL/6J) was obtained from the Ensembl project, and a 178kb 129/SvJ BAC clone (bMQ-213M15) containing exon 2 of the *Calcb* gene was obtained from Source Bioscience. A 9.5kb region (2kb-pre and 7.5kb-post first coding ATG of exon 2) from bMQ-213M15 was retrieved into a pBluescript-diptheria toxin A (PBS-DTA) plasmid via a first recombineering step. A Cre recombinase-estrogen receptor T2 (CreERT2) fusion-Frt-Neomycin-Frt-loxP cassette was targeted into the first coding ATG of exon 2 of the *Calcb* gene via a second recombineering step. The first 89bp of exon 2 (encoding the first 29 amino acids) were replaced. The targeting construct was linearized with AscI restriction enzyme (New England BioLabs). Homologous recombination was performed in mouse embryonic stem (ES) cells following standard procedures at Texas A&M Institute for Genomic Medicine (TIGM). Correctly targeted ES cell clones were identified by long-range PCR, sequencing (performed by TIGM) and karyotyping (UT MD Anderson Cancer Center). Targeted ES cells were microinjected into BL/6 blastocysts, and chimeras were mated to a germline-active FLPo line (JAX strain #012930) to remove the neomycin resistance cassette. *Calcb*$^{CreER/+}$ mice negative for the neomycin selection cassette were identified by PCR and then backcrossed and maintained as heterozygotes on a C57/BL6 background.

In vitro **culture models**

<u>Enteric neuro-glial cultures</u>—Newborn P0 mouse pups were euthanized and the small intestine with mesentery was isolated, cleaned, and placed in holding buffer—i.e., HBSS without Ca^{2+} and Mg^{2+} (Gibco), 5% FCS, 10mM HEPES (Gibco), 100 units mL^{-1} Pen-strep (Gibco), 100 µg mL^{-1} Gentamycin (Lonza)—until all tissues were collected. Small intestines were then minced with scissors and digested for 30 min at 37°C with magnetic stirring at 550 rpm in digestion buffer—

i.e., HBSS with Ca^{2+} and Mg^{2+} (Gibco), 5% FCS, 10mM HEPES (Gibco), 100 µg mL^{-1} DNase I (Roche), 200 µg mL^{-1} Liberase TH (Roche). After digestion, samples were gently triturated with 2-3 passes through an 18G needle, filtered with a 40µm strainer, and quenched by washing 3-times with ice-cold sterile DMEM/F-12 media (Gibco). Cells from each mouse were then plated in distinct wells of a 12-well low-adhesion plate and incubated at 37°C with 5-10% CO_2 for 6-7 days in Neurosphere Culture Media—i.e., DMEM/F-12 (Gibco), 10mM HEPES (Gibco), 100 units mL^{-1} Pen-strep (Gibco), 100 µg mL^{-1} Gentamycin (Lonza), 2mM Glutamine (Gibco), 55µM β-mercaptoethanol (Gibco), 1x B-27 Plus supplement (Thermo Scientific), 1x N2 supplement (Thermo Scientific), 20 ng mL^{-1} recombinant FGF-basic (PeproTech), 20 ng mL^{-1} recombinant EGF (PeproTech), 50 ng mL^{-1} recombinant GDNF (R&D Systems). Debris and dead cells were then gently removed with 2-3 media washes, centrifuging at 80 x g for 1-2 min to enrich the precipitate for neurospheres. Neurospheres were dissociated in Accutase (Stem Cell Technologies) for 15-20 min with gentle trituration. After counting cells, dissociated neurospheres were plated in flat-bottom 96-well plates (10,000-20,000 cells per well) or 8-well imaging slide chambers (Ibidi; 20,000-40,000 cells per well) coated with Matrigel (BD; 1:100 dilution in DMEM/F-12 for 2 hrs at 37°C) and incubated at 37°C with 5-10% CO_2 in Neuron Differentiation Media—i.e., Neurobasal A medium (Gibco), 10mM HEPES (Gibco), 100 units mL^{-1} Pen-strep (Gibco), 100 µg mL^{-1} Gentamycin (Lonza), 2mM Glutamine (Gibco), 55µM β-mercaptoethanol (Gibco), 1x B-27 Plus supplement (Thermo Scientific), 1x N2 supplement (Thermo Scientific), 50 ng mL^{-1} recombinant GDNF (R&D Systems). Experiments with cytokine and drug incubations began after day 10 and visible inspection of neuronal networks in culture. After the conclusion of experiment, wells were washed once gently with PBS and RNA from cultures was isolated for bulk RNA sequencing or RT-qPCR by the addition of 5-7 µL of Buffer TCL (Qiagen) supplemented with 1% β-mercaptoethanol (Sigma-Aldrich) or 100µL of Extraction Buffer from the PicoPure™ RNA Isolation Kit (Arcturus), respectively.

Dorsal Root Ganglia (DRG) neuron culture—Adult mice were euthanized by CO_2 narcosis, perfused through the heart with PBS, and vertebral columns were isolated and cleaned of muscle and connective tissue. The vertebral column was then cut in half sagittally at the median, and the spinal cord was removed gently from each half of the spinal column in a rostral to caudal direction, making sure not to disrupt the DRGs. All DRGs were dissected from mice and pooled together

and dissociated and cultured as previously described (*29*). Cultures were administered cytokines after 1-2 days of culture and RNA for qPCR analysis was isolated from each well using the PicoPure™ RNA Isolation Kit (Arcturus) as per manufacturer's instruction.

Bone marrow-derived macrophage (BMDM) culture—Mouse BMDMs were isolated, differentiated, and cultured as previously described (*30*) with several minor modifications. Briefly, bone marrow progenitors were isolated from the femurs and tibia of mice and cultured in DMEM (high glucose) (Gibco) supplemented with 10% FCS, 100 units mL^{-1} Pen-strep (Gibco), 10mM HEPES (Gibco), 1% GlutaMAX (Gibco), 1% NEAA (Gibco), and 20 ng mL^{-1} macrophage-colony stimulating factor (M-CSF) (Peprotech). Cells were plated at 1 x 10^6 cells per 60mm non-TC treated plates and incubated at 37°C with 10% CO_2 for 7 days, adding media on day 2 and exchanging media on day 5. On day 7, cells were dissociated from non-TC plates using Versene (Gibco) with gentle pipetting and replated and cultured overnight in 24-well TC-treated plates at 2 x 10^5 cells per well. On day 8, the media was removed and replaced with serum-free media with 10 ng mL^{-1} M-CSF, serum-starved for 2 hours, and treated with compounds for 8 hours. At the end of the assay, cells were washed with PBS and lysed with Buffer RLT + 1% β-mercaptoethanol and RNA was extracted using the Qiagen RNeasy Mini Kit as per the manufacturer's instructions.

Primary small intestinal macrophage (SI-Mφ) culture—Mouse proximal small intestines were processed according to "Isolation of muscularis propria and lamina propria for single-cell suspension" protocol below with minor modifications. Briefly, mice were euthanized by CO_2 narcosis and perfused with PBS through the left ventricle. The proximal 15 centimeters of small intestines were dissected on ice, cut longitudinally along the mesenteric border, and cleaned of luminal contents. The proximal SI samples were stripped of epithelial cells by sequential washing and occasional vortexing in ice-cold IEC isolation buffer with DTE (HBSS without Ca^{2+} and Mg^{2+}, 5% FCS, 10mM HEPES, 5mM EDTA, 1mM DTE) followed by 2-3 additional washes of the same buffer without DTE, and finally rinsed with PBS without EDTA. Proximal SI samples were then minced with scissors and digested in digestion buffer (HBSS with Ca^{2+} and Mg^{2+}, 5% FCS, 10mM HEPES, 100 µg mL^{-1} DNase I, 200 µg mL^{-1} Liberase TL enzyme) for 20-30 minutes at 37°C in flasks with stir-bars spinning at 550 rpm. After digestion, samples were gently triturated with 3-5 passes through an 18G needle attached to a syringe and single-cell suspensions were strained

through 40μm nylon meshes, washed, stained with appropriate antibodies, and singlet/viability dye negative/CD45+/SiglecF^{neg}/CD11b+/CD64+/F480+ cells were FACS purified, plated in 96-well TC-treated plates, and cultured overnight in RPMI media (Sigma-Aldrich) supplemented with 10% FCS, 100 units mL^{-1} Pen-strep (Gibco), 10mM HEPES (Gibco), 1% GlutaMAX (Gibco), 1% NEAA (Gibco), and 20 ng mL^{-1} M-CSF (Peprotech). The next day, media was removed and replaced with serum-free media with 10 ng mL^{-1} M-CSF, serum-starved for 2 hours, and treated with compounds for 8 hours. At the end of the assay, cells were washed with ice-cold, sterile-filtered PBS supplemented with 5mM EDTA and 5% BSA, and lysed in 100μL Extraction Buffer from the PicoPure^{TM} RNA Isolation Kit (Arcturus). RNA was isolated and processed according to the manufacturer's instructions.

Infections and *in vivo* treatments

Primary* H. Polygyrus *infection and quantification—L3-stage *H. polygyrus* larvae stocks were kindly provided by Hai Ning Shi and Chien Wen Su and propagated in C57Bl/6J mice. Fresh stocks were generated every 5-6 months, as previously described (*31*). For primary infection, mice were administered 200 L3 larvae by oral gavage and euthanized at various timepoints as indicated.

To quantify luminal parasite burden, a modified Baermann's apparatus was used. Briefly, the proximal 20 cm. of the small intestine was dissected longitudinally at the mesenteric border and the luminal contents were gently collected in the center of a rectangular piece of single-layered surgical gauze. The corners of the gauze were then brought together, and a toothpick was inserted through the collected corners, forming a pouch. The gauze pouch was then suspended by the toothpick in a beaker or conical flask filled with PBS and incubated overnight at 37°C. The adult worms that fell to the bottom of the flask or beaker were sexed and quantified using a stereomicroscope. Occasionally, worms were fixed in 4% PFA for later quantification.

For fecal egg quantification, fecal pellets and solid colonic contents were combined and weighed. Fecal contents were then dissociated and mixed with a saturated solution of NaCl and glucose, filtered with gauze to remove large undissolved debris, and loaded into a McMaster chamber slide for egg quantification under a light microscope.

For implanted larvae quantification, the proximal 20 cm. of the small intestine was removed, placed on a metal tray in an ice bucket along the length of a ruler and a series of images

were acquired from a fixed distance using an iPhone 11. FIJI was used to scale distance measurements for each image to a known distance measurement on the ruler, and ellipse-shaped regions of interest were drawn manually around the borders of implanted larvae. The number and area of each region of interest was then quantified using FIJI.

For most experiments, experimenters were blinded to mouse genotype at the time of organ collection, luminal worm quantification, and fecal egg quantification. For all experiments, experimenters were blinded to mouse genotype at the time of implanted larvae quantification and at the time of infection, except for experiments that required specific genotypes to be further divided into treatment groups.

IL-4 and IL-13 complex administration—Recombinant mouse IL-4 cytokine:anti-IL-4 antibody complexes were generated as previously described to significantly extend the *in vivo* half-life and biological activity of IL-4 (*32*). Briefly, recombinant mIL-4 (Miltenyi Biotec, 130-097-757) was mixed with anti-mIL-4 monoclonal antibody clone 11B11 (BioXcell) at a 1:5 (weight/weight) ratio (~2.3:1 molar ratio), incubated at 37°C for 2-3 min, then at RT for 5 min, and diluted in PBS to an appropriate volume. Mice were administered daily intraperitoneal (i.p.) injections of IL-4 complex (1µg IL-4 + 5µg anti-IL-4 per injection) in 200µL for 3-4 days, as indicated. Mice were then euthanized either 24 h after the 3rd dose or 1.5 h after the 4th dose. The IL-13 cytokine:anti-IL-13 antibody complexes were generated similarly to the IL-4 complexes and mice were administered 1µg recombinant mIL-13 (BioLegend, 575904) + 5µg anti-mIL-13 antibody clone eBio13A (Invitrogen, 14-7133-81) per injection. Experimenters were not blinded to treatment group during treatment administration.

Tamoxifen administration—Tamoxifen (Sigma-Aldrich, T5648-5G) was dissolved to a concentration of 20 µg mL^{-1} in corn oil for at least 3 hours at 37°C in the dark with a magnetic stir bar. Once tamoxifen was fully dissolved, mice were administered 8µg daily by oral gavage for either 3 or 5 days. Downstream experiments commenced 6-7 days after initial tamoxifen dose.

EdU incorporation—For the first 3 days, mice were administered a dose of 100 µg of 5-ethynyl-2'-deoxyuridine (EdU) in PBS by intraperitoneal injection ~20 to 25 min. after each IL-4 complex treatment. On the 4th day, mice were given 2 doses of EdU spaced 1.5 hours apart, and mice were

euthanized 30 min. following the final EdU dose. Tissues were fixed and processed as in the "Wholemount muscularis immunostaining" section and EdU was visualized by using the Click-iT™ Plus EdU Cell Proliferation Kit for Imaging, Alexa Fluor™ 647 dye (Thermo Fisher Scientific, C10640).

RNA isolation and RT-qPCR

Adult mice were euthanized by CO_2 narcosis, perfused through the heart with PBS, and small intestines and colons were dissected out and kept in ice-cold holding buffer (HBSS without Ca^{2+} and Mg^{2+}, 5% FCS, 10mM HEPES) until all samples were collected. Segments of duodena (2 cm to 4 cm distal to the pyloric sphincter), ilea (0 cm to 2 cm proximal to the ileo-cecal junction), or proximal colons (0 cm to 2 cm distal to the ileo-cecal junction) were dissected out, cleared of mesenteric fat, cut open longitudinally, and gently cleaned of luminal contents and mucus before submerging in RNA*later* Stabilization Solution (Thermo Fisher Scientific) and stored at +4°C until RNA isolation—in some experiments the muscularis was separated from the lamina propria and processed separately at this point. Tissues were then homogenized in 1mL of Trizol using Lysing Matrix E tubes (MP Biomedicals, 116914050) with the BeadBug™ microtube homogenizer (Benchmark Scientific) in two 3000 rpm rounds (120 sec and 90 sec, respectively), resting tubes on ice in between rounds. Total RNA was extracted from the Trizol homogenate with the addition of chloroform (Sigma-Aldrich) and centrifugation (12,000 x g, 15 min at +4°C) in Phasemaker™ Tubes (Thermo Fisher Scientific, A33248). The aqueous phase containing total RNA was transferred to a new tube and Sodium Acetate (Thermo Fisher Scientific, R1181) and RNA-grade Glycogen (Thermo Fisher Scientific, R0551) was added prior to isopropanol precipitation. After precipitation and centrifugation, RNA was washed 4-5 times in 75% ethanol, resuspended in nuclease-free H_2O, and quantified on a Nanodrop Spectrophotometer (Thermo Fisher). RNA was converted to cDNA using the iScript Reverse Transcription Supermix (BioRad, 1708841) as per manufacturer's instructions. RT-qPCR was performed on an ViiA 7 Real-Time PCR system (Applied Biosystems) using TaqMan Fast Advanced Mastermix (Thermo Fisher Scientific, 4444963). Each RT-qPCR reaction was internally normalized to the Mouse *Actb* Endogenous Control VIC/MGB probe (Thermo Fisher Scientific, 4352341E) and gene expression was assessed using TaqMan FAM/MGB probes (see associated antibody and reagent table). Relative gene expression is represented as either $2^{-\Delta CT}$ values, ΔCT = (gene of interest CT) - (*Actb* CT), or as 2^{-}

$^{\Delta CT}$ values normalized to the mean of the control or experimental group per independent experiment (i.e., fold-change). For muscularis and lamina propria *H. polygyrus* time course samples in **Figure 2B**, gene expression of *Tubb3*, *Nmu*, *Calcb*, and *Vip* were first represented as $2^{-\Delta CT}$ values relative to the respective internal *Actb* control VIC/MGB probe and subsequently normalized to *Tubb3* expression (i.e., relative neuronal content). Values were then expressed as a fold-change relative to naïve, H_2O-administered mice.

Tissue Homogenization and Preparation for ELISA

Mice were euthanized by CO_2 narcosis and perfused with PBS through the left ventricle. The middle 6 cm of the small intestine (jejunum) or 2-4 cm of the distal ileum were excised, cut longitudinally at the midline, and cleaned. The jejunum and ileum were chosen over the duodenum to avoid potential protein degradation by the greater digestive protease concentrations in the proximal SI. For some experiments, the muscularis was then separated from the lamina propria by stereo microdissection (jejunum).

For the CGRP EIA kit (Cayman Chemical Company, Cat. #589001), tissues were homogenized in a hypotonic buffer (10 mM HEPES, 10 mM KCl, 1.5 mM $MgCl_2$, pH 7.4) supplemented with a Protease and Phosphatase Inhibitor Cocktail (Thermo Fisher, 78440). Of note, we found some detergents to be incompatible with the CGRP EIA. For the NMU ELISA kit, tissues were homogenized in 0.5% Triton X-100 in PBS supplemented with a Protease and Phosphatase Inhibitor Cocktail (Sigma-Aldrich, Cat. #11836170001).

The homogenized samples were subjected to two cycles of freeze-thaw to enhance cell lysis. Following this, the lysates were centrifuged at 500 × g for 5 minutes at 4°C. The supernatants were carefully collected and used for subsequent ELISA analysis.

Gastrointestinal transit assays

Naïve and infected mice were fasted overnight or for 4 hours, respectively, and placed individually into cages without food and with minimal bedding to acclimate for at least an hour before proceeding with gastrointestinal transit assays. All transit assays were initiated between 1.5 ZT - 3.5 ZT (8:30am-10:30am).

Carmine red total gastrointestinal transit time (GITT)—Mice were orally gavaged with a 200µL bolus of 6% (w/v) carmine red dye (Sigma-Aldrich, C1022-25G) in 0.5% (w/v) methylcellulose (Fisher Scientific) dissolved in ddH2O and returned to their individual cages. Total GITT was determined as the duration of time between gavage and the defecation of the first red-colored fecal pellet.

Evans blue small intestinal transit assay—Mice were orally gavaged with a 100µL bolus of 25mg mL^{-1} Evans blue (Sigma-Aldrich) in 0.5% (w/v) methylcellulose (Fisher Scientific) dissolved in ddH2O and returned to their individual cages. Exactly 40 min after oral gavage, mice were euthanized and the stomach, small intestine, cecum, and colon were excised. The "% of SI dye traveled" was calculated as the percentage of the total small intestinal length that the most distally observable blue dye travelled in the small intestine. For "geometric center (SI)" readings, the small intestine of each mouse was cut into 10 equally sized segments, and each segment was gently minced with scissors in 10mL of NaOH (0.1N) and incubated at room temperature overnight in the dark. Samples were centrifuged to pellet debris, and supernatant was transferred to a 96 well plate and absorbance at 560nm (A_{560}) measured on a spectrophotometer. Geometric center (SI) was calculated as $\Sigma(A_{560} \times \text{segment number}) \div \Sigma(A_{560})$, with segment number being a number from 1 to 10 representing the most proximal to most distal segments, respectively.

Evans blue gastric emptying assay—The stomach of each mouse was placed into 20mL of NaOH (0.1N), gently minced with scissors, and incubated at room temperature overnight in the dark. Samples were centrifuged to remove debris, and supernatants were further diluted 1:10 in NaOH (0.1N). To calculate the combined A_{560} of all small intestinal segments, 1 mL of liquid from each segment of the small intestine transit assay was combined in a new tube totaling 10mL (equivalent to mincing entire small intestine in 100mL) and further diluted 1:1 in NaOH (0.1N) to have an equivalent dilution factor to the stomach samples. "% gastric emptying" was calculated as $100 \times [A_{560}(\text{SI combined}) \div (A_{560}(\text{Stomach}) + A_{560}(\text{SI combined}))]$.

Immunostaining and confocal microscopy

Mice were euthanized by CO_2 narcosis and perfused with PBS through the left ventricle.

Wholemount muscularis immunostaining—6-centimeter segments of duodena (generally between 4-cm. and 10-cm. distal to the pyloric sphincter) were dissected on ice, cut longitudinally along the mesenteric border, laid flat (luminal side down) on a Sylgard-coated plate, pinned flat using insect pins, and held on ice in holding buffer (HBSS without Ca^{2+} and Mg^{2+}, 5% FCS, 10mM HEPES) until all samples were collected. Samples were then fixed with 4% paraformaldehyde (PFA) at 4°C overnight, washed 3x with PBS, and the muscularis propria was then isolated from lamina propria by stereomicroscope-aided microdissection. Muscularis samples were cut into 1 to 1.5 cm. segments and stored in 24- or 48-well plates with PBS at 4°C until ready to stain.

Samples were permeabilized for 30 min at room temperature (RT) on a shaker with 1% Triton X-100 in PBS. Next, samples were blocked for 2-3 hours at RT on a shaker in PTxwH buffer (PBS, 0.05% Tween-20, 0.5% Triton X-100, 4 µg mL^{-1} heparin sulphate) containing 10% Normal Donkey Serum (NDS) or 10% Normal Horse Serum (NHS), mouse IgG (1:500), and/or rat IgG (1:500), depending on the primary antibody compatibility. After blocking, samples were stained for 1-2 days at 4°C on a shaker with primary antibodies diluted in blocking buffer, and then washed 6-8x in PTxwH buffer at RT on a shaker for 30 min per wash. Samples were next stained overnight at 4°C on a shaker with appropriate concentrations of fluorophore-conjugated secondary antibodies diluted in blocking buffer, washed 6-8x in PTxwH buffer at RT on a shaker, mounted in Prolong Glass Antifade Mounting Solution (Thermo Fisher, P36980) and cured for 48 hours at RT in the dark. Occasionally, samples were stained with DAPI prior to mounting.

Enteric neuron quantification—For quantification, 8-10 fields of view (FOV) centered on contiguous neuronal cell bodes per sample were randomly selected across the tissue and z-stacks were acquired using the 20x objective on a Zeiss LSM880 Confocal Microscope. Alternatively, tile scans capturing FOVs using 40x-oil or 63x-oil objectives were used to image randomly selected ganglia, defined loosely as regions of contiguous neuronal cell bodies. Where applicable, general stains for neurons (β3-tubulin) and neuronal cell bodies (Hu C/D, ANNA-1) were used to select FOVs and to center on ganglia. Maximum and average z-stack projections were used for segmenting cells and quantifying fluorescent intensity, respectively; all image quantifications were performed using custom-made FIJI macros or by blinded manual counting in FIJI. Because fluorescent intensities of neuronal cell body stains (e.g., HuCD, ANNA-1) can differ between experiments and change the average size of segmented neurons, we defined ‘large’ neurons as

neurons that were above the 3rd quartile in area (μm^2) across all segmented neurons of a given experiment.

Tissue clearing and 3D rendering—Duodenal tissues containing implanted *H. polygyrus* larvae (6 d.p.i.) were processed and stained as described in the wholemount muscularis immunostaining method and subsequently cleared for 30 min in the dark at room temperature with gentle rotation using the FOCM clearing reagent, prepared as previously described (*33*). Next, tissues were further cleared for 12 hours or overnight in the dark at room temperature with gentle rotation using the Ce3D clearing reagent, prepared as previously described (*34*). Each sample was mounted flat on the coverslip of a 35mm imaging dish with high tolerance #1.5 glass coverslip bottom (MatTek, P35G-0.170-14-C) and Ce3D was added to cover tissue; an additional #1.5 coverslip was added on top of the tissue to secure it into place. Images were acquired using the 20x objective on a Zeiss LSM880 Confocal Microscope with a voxel density of at least 1024 x 1024. Tile scans and z-stacks of at most 1 µm-thickness per slice were used to capture a large field of view with a depth of up to approximately 150 µm into the cleared tissue. 3D images and videos were rendered and cropped using Aivia software.

Intestinal cross-sections (Swiss rolls)—10-centimeter segments of small intestine (generally between 4 cm. and 20 cm distal to the pyloric sphincter) were dissected, cut longitudinally along the mesenteric border, and laid flat (luminal side up) on C-fold towels dampened with ice-cold holding buffer. Luminal contents were gently removed, and the intestine was loosely rolled from the proximal side to the distal side using a toothpick. Samples were then fixed in 4% PFA at 4°C overnight, rinsed in PBS, and subject to 30% sucrose cryopreservation for at least 24 hours or until tissues sank. Excess sucrose was blotted away and rolled intestines were oriented circle face-down and embedded into blocks using OCT media, snap frozen in a dry ice:2-methylbutane slurry and stored at -20°C before cryosectioning at 20µm-thickness onto pre-cleaned slides.

Slides were permeabilized with 0.25% Triton-X100 in PBS for 10 min, washed 2-3x with PBS-T (PBS + 0.1% Tween-20), and (when using biotin-conjugated primary antibodies) blocked for endogenous biotin using Streptavidin/Biotin Blocking Kit (Vector Labs, SP-2002) as per kit instructions followed by additional PBS-T washes. Slides were next administered Image-iT™ FX Signal Enhancer (Thermo Fisher, I36933) for 30 min at RT, washed 2-3x with PBS-T, blocked for

1-1.5 hours at RT with blocking buffer (PBS-T, 10% NDS or 10% NHS, 1:500 mouse IgG [~6 μg mL^{-1} final], and/or 1:500 rat IgG [~23.2 μg mL^{-1} final]), and stained with primary antibodies in blocking buffer at 4°C overnight. Next, slides were washed 3-4x in PBS-T and stained with fluorophore-conjugated secondary antibodies and/or fluorophore-conjugated streptavidin for 1-1.5 hours at RT in the dark, washed at least 4x in PBS-T, optionally stained with DAPI solution for 10 min, mounted in Prolong Glass Antifade Mounting Solution and cured for 48 hours at RT in the dark.

Villus fiber quantification—For quantification, a large region of continuous villi consisting of 6-9 tiles and 8-11 z-stack slices (1μm-thickness) was imaged per replicate using the 20x objective of a Zeiss LSM880 Confocal Microscope. For CGRP fiber quantification, maximum projections of z-stacks were performed, and 6-15 rectangular regions of interest (ROI) were randomly selected on intact villi (from tip to base, excluding the crypt) per sample. For calretinin fiber quantification maximum projections of z-stacks were performed and 6-15 regions of interest were randomly selected on intact villi (from tip to base of crypt) per sample. Using custom made FIJI macros, binary masks of each stain were generated and the $CGRP^{+}$ or $calretinin^{+}$ fiber densities were respectively calculated as percent mask area per $DAPI^{+}$ mask area within each ROI.

Quantification of epithelial cell subsets—8μm-thick intestinal cross-sections stained with antibodies for epithelial cell subsets and DAPI were imaged on a Zeiss AxioScan7 using the 20x objective. To preserve system memory, the second-level of image resolution was opened in FIJI and the image was cropped into 4-6 smaller files focused on intact villi for quantification. Custom FIJI macros were designed to segment all DAPI+ nuclei and EpCAM+DAPI+ epithelial cell nuclei. Next, EpCAM+DAPI+ nuclei were assessed for cell type-specific marker positivity. Marker-positive epithelial nuclei were represented as % of all EpCAM+DAPI+ epithelial cells.

Imaging of enteric neuro-glial cultures—After neurosphere dissociation with Accutase, enteric neurons and glia were plated in sterile 8-well Matrigel- and Poly-L-Lysine-coated #1.5 coverslip imaging chambers (Ibidi, 80824), differentiated into enteric neurons as per the “Enteric neuro-glial cultures” section, and treated with experimental compounds. After experimental treatment, neuro-glial cultures were fixed in 4% PFA for 15 min at room temperature and either permeabilized in

methanol for 20 minutes at -20°C or in 0.25% Triton X-100 for 10 minutes at room temperature. All washes were carried out in either PBS + 0.3% Triton X-100 or PBS + 0.1% Tween-20 for p-STAT6 and IL-13RA1 staining, respectively. Slide chambers were next incubated in Image-iT^{TM} FX Signal Enhancer (Thermo Fisher, I36933) for 30 min at RT, washed 2-3x, blocked for 1 hour at RT with blocking buffer (wash buffer, 10% NDS or 10% NHS, 1:500 mouse IgG [~6 μg mL^{-1} final], and/or 1:500 rat IgG [~23.2 μg mL^{-1} final]), and stained with primary antibodies in blocking buffer at 4°C overnight. Next, slide chambers were washed 3-4x and stained with fluorophore-conjugated secondary antibodies and/or fluorophore-conjugated streptavidin for 1.5 hours at RT in the dark, washed at least 4x, optionally stained with DAPI solution for 10 min, mounted in Prolong Glass Antifade Mounting Solution and cured for 48 hours at RT in the dark.

For quantification of p-STAT6 staining, 8-20 FOV were taken randomly using either the 20x or 63x oil objectives (same objective per experiment) on a Zeiss LSM880 Confocal Microscope, and the nuclear and non-nuclear fluorescence intensities of anti-phospho-STAT6 (Tyr641) antibody staining (Cell Signaling Technology, Cat. No. 56554) were quantified per individual β3-tubulin^{+} neurons in FIJI using custom made macros.

Isolation of peripheral nervous system ganglia

Mice were euthanized by CO_2 narcosis and perfused with PBS through the left ventricle. For imaging analysis after isolation, all ganglia were fixed in 4% PFA at 4°C overnight, rinsed in PBS, cryopreserved in 30% sucrose for at least 24 hours or until tissues sank, and embedded, sectioned, stained, and imaged as previously described for intestinal cross-sections.

DRG isolation—DRG corresponding to T10-13 vertebrae (i.e., vertebrae of the lesser thoracic splanchnic nerve innervating the midgut) were isolated as previously described (*29*). Briefly, the vertebral columns of PBS-perfused mice were isolated with rib cages trimmed, but intact, and cleaned of muscle and connective tissue. The vertebral column was then cut in half sagittally at the median, and the spinal cord was removed gently from each half of the spinal column in a rostral to caudal direction, making sure not to disrupt the DRGs. Through stereomicroscope-aided microdissection, the corresponding DRGs were identified and isolated using the rib cage as a landmark.

Nodose-Petrosal-Jugular ganglia (N-JG) isolation—the left and right N-JG were isolated as previously described (*35*). Briefly, euthanized mice were decapitated, and the skull was isolated and cleaned of connective tissue. Two cuts were made through the eye sockets to open the top of the skull, and the brain was gently removed in a rostral to caudal direction. Using a stereomicroscope, the brainstem and left and right vagus nerves were identified and traced to their respective jugular foramen. The jugular foramen on each side was gently teased opened with forceps exposing the N-JG, which was gently isolated.

Brainstem isolation—the brainstem and left and right vagus nerves were identified as in the "Nodose-Petrosal-Jugular ganglia (N-JG) isolation" procedure above, and the brainstem was cut 2 millimeters above and below where the vagus nerve originates (4 mm. total). The vagus nerves were trimmed from the brainstem and the brainstem was submerged in RNALater and stored at +4C until RNA isolation.

Celiac Ganglion-Superior Mesenteric Ganglion (CG-SMG) isolation—the CG-SMG, which provides visceral sympathetic innervation to the gut, was identified by using a stereomicroscope to trace the inferior vena cava (IVC) to where it meets the left renal vein—the CG-SMG is the translucent bundle of neurons and fibers running just above the intersection of these two blood vessels. The CG-SMG was gently teased apart from the left renal vein and the IVC and dissected out.

Isolation of mesenteric lymph nodes for single-cell suspension

Mice were euthanized by CO_2 narcosis and perfused with PBS through the left ventricle. Mesenteric lymph nodes were isolated, cleaned of mesentery fat, smashed through a sterile 40 μm mesh strainer to form a single-cell suspension, and washed at least once in PBS supplemented with 5% FBS before proceeding to flow cytometry staining or other downstream assays.

Different gut-draining mesenteric lymph nodes were isolated similarly to Esterhazy et al., (2019)(*36*). Briefly, the hepatic/celiac LN, which co-drains the duodenum, was denoted "D1" and identified by its enlargement after *H. polygyrus* infection and its location bordering the liver and portal vein outside of the main mesenteric lymph node chain. The distal duodenum- and jejunum-draining LNs ("D2-Jej") were analyzed together and identified anatomically as the 3-4 most

proximal LNs on the main mesenteric LN chain that were enlarged after *H. polygyrus* infection and had lymphatic vessels extending to the duodenum and jejunum. The ileum- and cecum/colon-draining lymph nodes ("Ile-Col") were analyzed together and identified anatomically as the 3 most distal LNs on the main mesenteric LN chain with lymphatic vessels extending to the distal small intestine and the cecum. Single-cell suspensions of isolated gut-draining lymph nodes were processed in the same way as the whole MLN.

H. polygyrus antigen recall assay

Mesenteric lymph node single-cell suspensions from *H. polygyrus* infected mice (14 d.p.i.) were counted and plated at 5 x 10^6 cells per well in a 24-well tissue culture plate in culture medium. *H. polygyrus* adult worm homogenate (AWH) was acquired as a gift from Hai Ning Shi (MGH) and was prepared as previously described (*37*). Various concentrations of AWH were added to wells and cells were cultured at 37°C with 10% CO_2 for 60 h before supernatants were removed, centrifuged, and snap-frozen for later analysis. For each biological replicate, separate wells were coated with 10 µg mL^{-1} anti-CD3 antibody (BioXcell, BE0002, clone 17A2) prior to plating cells to measure general/non-specific T-cell responses in the absence of AWH.

Bead-based immunoassays

Cytokine concentrations from recall assay culture supernatants and tissue homogenates were measured using the LegendPlex Mouse T Helper Cytokine Panel (12-plex) (BioLegend, 741044) as per manufacturer's instruction. Prepared samples were acquired on a LSRII flow cytometer (BD Biosciences) and analyzed with LegendPlex Data Analysis Software. To aid visualization on a log-scale without excluding datapoints, analytes below the limit of detection were set to 0.5 pg mL^{-1} and plotted. To determine antigen-specific T-cell responses in recall assay, analyte concentrations from 25 µg mL^{-1} AWH-treated cultures were represented as a percentage of the corresponding analyte concentrations from plate-bound anti-CD3-treated cultures.

Isolation of muscularis propria and lamina propria for single-cell suspension

Mice were euthanized by CO_2 narcosis and perfused with PBS through the left ventricle. The proximal or distal 12-20 centimeters of small intestines were dissected on ice, cut longitudinally along the mesenteric border, and laid flat (luminal side down) on C-fold towels dampened with

ice-cold holding buffer (HBSS without Ca^{2+} and Mg^{2+}, 5% FCS, 10mM HEPES) on a metal tray in an ice bucket. Muscularis propria were isolated from lamina propria by stereomicroscope-aided microdissection performed on an ice-cold metal block lined with buffer-dampened C-fold towels, rinsed 2-3x in holding buffer to limit epithelial cell contaminants, and processed separately from lamina propria samples. The lamina propria samples were stripped of epithelial cells by sequential washing and occasional vortexing in ice-cold IEC isolation buffer with DTE (HBSS without Ca^{2+} and Mg^{2+}, 5% FCS, 10mM HEPES, 5mM EDTA, 1mM DTE) followed by 2-3 additional washes of the same buffer without DTE, and finally rinsed with PBS without EDTA. Both the muscularis and lamina propria samples were separately minced with scissors and digested in digestion buffer (HBSS with Ca^{2+} and Mg^{2+}, 5% FCS, 10mM HEPES, 100 μg mL^{-1} DNase I, Liberase enzyme) for 15-30 minutes at 37°C in flasks with stir-bars spinning at 550 rpm. Liberase concentrations of 100 μg mL^{-1} and 200 μg mL^{-1} were used for muscularis and lamina propria samples, respectively. Liberase TL and Liberase TH were used to digest tissues for flow cytometric analysis or scRNA-seq, respectively. After digestion, samples were gently triturated with 3-5 passes through an 18G needle attached to a syringe and single-cell suspensions were strained through 40μm nylon meshes, washed, and further processed for FACS analysis or purification.

Isolation of duodenal crypt epithelial cells for single-cell suspension

Mice were euthanized by CO_2 narcosis and perfused with PBS through the left ventricle. The proximal 10 centimeters of small intestines were dissected on ice, cut longitudinally along the mesenteric border, and luminal contents were gently removed. Intestines were cut into ~1 cm segments and placed in epithelial dissociation buffer (PBS with 5mM EDTA) for 30 min on ice with mild shaking. The first fraction of buffer was discarded, and fresh epithelial dissociation buffer was added to the samples and tubes were kept on ice for 30 min with occasional vigorous shaking. The second fraction of buffer was saved, and the previous step was repeated with a third fraction of epithelial dissociation buffer. The second and third fractions containing epithelial cells enriched for crypts were combined and strained through a sterile 70 μm mesh filter and pelleted by centrifugation. Epithelial crypts were further dissociated with the addition of 10 mL of pre-warmed (37°C) TrypLE solution (Gibco) for 3 min. Ice-cold DMEM/F-12 media supplemented with 5% FBS was added to quench the reaction, and cells were filtered through a sterile 40 μm

mesh strainer. Cells were counted and resuspended in PBS supplemented with 0.4% BSA for 10x Genomics scRNA-seq encapsulation.

Flow cytometry and fluorescence-activated cell sorting (FACS)

Single-cell suspensions were first stained with either eFluor506 fixable viability dye (1:800 dilution; eBioscience™, 65-0866-14), Ghost Dye™ Violet 510 (1:800 dilution; Tonbo, 13-0870-T500), or Ghost Dye™ Red 780 (1:800 dilution; Tonbo, 13-0865-T500) diluted in PBS for 12-15 min at room temperature, as per manufacturer's instructions. Then, cells were washed in FACS buffer (PBS supplemented with 2% FBS and 2mM EDTA) and blocked for 5-10 min on ice in blocking buffer—i.e., FACS buffer supplemented with 1:200 dilution of TruStain FcX™ PLUS (anti-mouse CD16/32) Antibody (clone S17011E) (BioLegend, 156604), 1:500 dilution of Rat IgG (Invitrogen, 31933, ~23.2 μg mL^{-1} final), and 1:500 dilution of Mouse IgG (Invitrogen, 10400C, ~6 μg mL^{-1} final). Without washing, a 2x concentrated cocktail of fluorophore-conjugated primary antibodies specific for various cell surface antigens was diluted in blocking buffer and added to the cells (1:1 dilution), which were stained on ice for 20-30 min in the dark.

The following antibody clones were purchased in various fluorophore-conjugated formats from BD Biosciences: anti-CD11b (clone M1/70), anti-CD4 (clone GK1.5), anti-CD45 (clone 30-F11), anti-CD8α (clone 53-6.7), anti-Ly6G (clone 1A8), anti-Siglec F (clone E50-2440), anti-TCRβ (clone H57-597), anti-CD301b (clone URA-1), anti-CD206 (clone Y17-505), anti-TCRγδ (clone GL3), anti-CD19 (clone 1D3). The following antibody clones were purchased in various fluorophore-conjugated formats from Thermo Fisher Scientific: anti-CD29 (Integrin beta 1) (clone eBioHMb1-1), anti-TER-119 (clone TER-119), anti-CD117 (clone 2B8). The following antibody clones were purchased in various fluorophore-conjugated formats from BioLegend: anti-CD11b (clone M1/70), anti-CD19 (clone 6D5), anti-CD31 (clone 390), anti-CD3ε (clone 145-2C11), anti-CD45R/B220 (clone RA3-6B2), anti-CD64 (clone X54-5/7.1), anti-CD90.2 (Thy1.2) (clone 30-H12), anti-I-A/I-E (clone M5/114.15.2), anti-Integrin β7 (clone FIB504), anti-KLRG1 (clone 2F1/KLRG1), anti-Ly6C (clone HK1.4), anti-NK1.1 (clone PK136), anti-PDGFRα (clone APA5), anti-CD196 (clone 29-2L17), TruStain FcX™ PLUS (anti-mouse CD16/32) Antibody (clone S17011E), Rat IgG2a, κ Isotype Control Antibody (clone RTK2758).

The following muscularis and lamina propria cell populations were identified using the indicated marker expression after gating on live (viability dye negative) single cells: KLRG1$^+$ ILC2 (*for FACS purification*)(CD45$^+$ CD90.2$^+$ Ter-119^{neg} NK1.1^{neg} B220^{neg} CD19^{neg} CD3e^{neg} KLRG1$^+$), GATA3$^+$ ILC2 (CD45$^+$ CD90.2$^+$ CD3e^{neg} NK1.1^{neg} RORγt^{neg} GATA3$^+$), NK cells (CD45$^+$ CD90.2$^+$ CD3e^{neg} GATA3^{neg} NK1.1$^+$), CD4+ αβT cells (CD45$^+$ CD90.2$^+$ CD3ε$^+$ TCRβ$^+$ TCRγδ^{neg} CD4$^+$ CD8α^{neg}), CD8+ αβT cells (CD45$^+$ CD90.2$^+$ CD3ε$^+$ TCRβ$^+$ TCRγδ^{neg} CD4^{neg} CD8α$^+$), T_H2 cells (CD45$^+$ CD90.2$^+$ CD3ε$^+$ TCRβ$^+$ TCRγδ^{neg} CD4$^+$ CD8α^{neg} GATA3$^+$ FoxP3^{neg}), T_{reg} cells (CD45$^+$ CD90.2$^+$ CD3ε$^+$ TCRβ$^+$ TCRγδ^{neg} CD4$^+$ CD8α^{neg} FoxP3$^+$), γδT cells (CD45$^+$ CD90.2$^+$ CD3ε$^+$ TCRβ^{neg} TCRγδ$^+$), Macrophages (CD45$^+$ CD3e^{neg} Ly6G^{neg} Siglec-F^{neg} CD11b$^+$ CD64$^+$), Eosinophils (CD45$^+$ CD3e^{neg} Ly6G^{neg} CD11b$^+$ CD64^{neg} Siglec-F$^+$ SSC-A^{high}), Neutrophils (CD45$^+$ CD3e^{neg} Siglec-F^{neg} Ly6G$^+$ CD11b$^+$).

The following mesenteric lymph node cell populations were identified using the indicated marker expression after gating on live (viability dye negative) single cells: CD4+ αβT cells (CD45$^+$ CD90.2$^+$ CD3ε$^+$ TCRβ$^+$ TCRγδ^{neg} CD4$^+$ CD8α^{neg}), CD8+ αβT cells (CD45$^+$ CD90.2$^+$ CD3ε$^+$ TCRβ$^+$ TCRγδ^{neg} CD4^{neg} CD8α$^+$), T_H2 cells (CD45$^+$ CD90.2$^+$ CD3ε$^+$ TCRβ$^+$ TCRγδ^{neg} CD4$^+$ CD8α^{neg} GATA3$^+$ FoxP3^{neg}), T_{reg} cells (CD45$^+$ CD90.2$^+$ CD3ε$^+$ TCRβ$^+$ TCRγδ^{neg} CD4$^+$ CD8α^{neg} FoxP3$^+$).

For intracellular expression analysis, cells were stained for cell surface antigens as previously described. Cells were then fixed and permeabilized using the Fixation/Permeabilization Solution Kit (BD Biosciences, 554722) as per the manufacturer's instructions. Permeabilized cells were again blocked with blocking reagents—1:200 dilution of TruStain FcX™ PLUS (anti-mouse CD16/32) Antibody (clone S17011E) (BioLegend, 156604), 1:500 dilution of Rat IgG (Invitrogen, 31933, ~23.2 µg mL^{-1} final), and 1:500 dilution of Mouse IgG (Invitrogen, 10400C, ~6 µg mL^{-1} final)—diluted in 1x BD permeabilization buffer for 5-10 min at room temperature in the dark, and then stained in the dark for 45 minutes at room temperature with gentle shaking using a fluorophore-conjugated antibody cocktail diluted in 1x BD permeabilization buffer with supplemented blocking reagents. For intracellular antibodies: The following antibodies were purchased from eBioscience™: anti-Arginase 1 PE-Cyanine7 (clone A1exF5), anti-RELMα PE (clone DS8RELM).

For intranuclear/transcription factor staining, cells were stained for surface antigens as previously described and subsequently fixed using the eBioscience™ Foxp3 / Transcription Factor

Staining Buffer Set (Invitrogen, 00-5523-00) as per the manufacturer's instructions. Cells were again blocked with blocking reagents diluted in 1x eBioscience^{TM} permeabilization buffer and stained in the dark for 45 minutes at room temperature with gentle shaking using a fluorophore-conjugated antibody cocktail diluted in 1x eBioscience^{TM} permeabilization buffer with blocking reagents. For intranuclear antibodies: The following antibody clones were purchased in various fluorophore-conjugated formats from eBioscience^{TM}: anti-Ki67 (clone SolA15), anti-ROR gamma (t) (clone AFKJS-9), anti-GATA3 (TWAJ), anti-FoxP3 (clone FJK-16s).

Flow cytometric analysis was performed using a FACSymphony A5 analyzer (BD Biosciences) or FACSAria II sorter (BD Biosciences). Cell populations were sorted for bulk RNA-seq and scRNA-seq using a FACSAria II sorter (BD Biosciences) and an MA900 Multi-Application Cell Sorter (Sony), respectively. Analysis of flow cytometric data was performed using FlowJo (version 10).

Bulk RNA sequencing

Library preparation and sequencing—A modified version of the SMART-Seq2 protocol (*38*) was applied to generate full-length transcriptomic data from low-input samples—processed by the Klarman Cell Observatory and the Broad Institute Genomics Platform (GP). Briefly, ~2,000 FAC-sorted cells or the contents of a single neuro-glial culture well in a 96-well plate were lysed in 5 µL of Buffer TCL (Qiagen) supplemented with 1% β-mercaptoethanol (Sigma-Aldrich) and snap-frozen until ready for processing. RNA from lysate was cleaned-up using Agencourt RNAClean XP SPRI magnetic beads (Beckman Coulter, A63987) and first strand cDNA synthesis was performed using Maxima H-minus Reverse Transcriptase (Thermo Fisher Scientific, EP0753) and amplified using KAPA HiFi HotStart PCR ReadyMix (KAPA Biosystems, KK2602). cDNA concentration was assessed using Quant-iT PicoGreen dsDNA Assay Kit (Thermo Fisher Scientific) and subsequently normalized to 0.25 ng μL^{-1}. Library preparation was performed using the Nextera XT DNA Library Preparation Kit (Illumina, FC-131-1096), confirmed to have a size of 500 bp using a BioAnalyzer (Agilent), and pooled libraries were normalized to 2nM and denatured using 0.1 N NaOH prior to sequencing. Flow cell cluster amplification and sequencing were performed according to the manufacturer's protocols using the paired-end Illumina sequencing (2 x 38 bp) with 75 cycle Nextseq 500 high output V2 kit (Illumina).

Generation of count matrix and differential gene expression analysis—After sequencing, the Terra platform was used to run Cumulus (*39*) and broadinstitute_gtex workflows: cumulus/bcl2fastq (V.5) was used for demultiplexing BCL files into individual libraries, broadinstitute_gtex/fastqc_v1-0_BETA (V.2) was used for quality control of fastq files, and broadinstitute_gtex/rnaseq_fastq_star_rsem_rnaseqc_v1-2_BETA was used for STAR alignment (*40*) against the mouse reference genome (GRCm38 / mm10) and transcript quantification with RSEM (*41*). RSEM expected count data was used to generate count matrices and expected counts were rounded to the nearest whole number. Samples with fewer than 1 million reads were excluded from analysis. DESeq2 (*42*) was used to perform differential gene expression analysis based on the negative binomial distribution. DESeq2 Log_{10}(adjusted p-values) and Log_2(fold-change) were used to generate volcano plots, and normalized counts of biological replicates from the DESeq2 object were used to plot individual gene expression. Prism v9 was used to generate volcano plots and gene expression scatter and box plots. In **fig. S2B**, normalized counts from DESeq2 object were used to calculate the fold-change of IL-4- and IL-13-treated samples to the BSA control group for each indicated gene.

Single-cell RNA sequencing

Single-cell encapsulation, sequencing, and demultiplexing—Muscularis propria single-cell suspensions were FAC-sorted on a MA900 Multi-Application Cell Sorter (Sony), enriching for 50% total live cells and 50% stromal/glial cells (live $CD45^{neg}$ $ITG\beta1^{+}$) per sample. Duodenal epithelial single-cell suspensions were counted with trypan blue. Sorted muscularis cells and duodenal epithelial cells were each pelleted and resuspended in PBS with 0.4% BSA. A maximum of 10,000 cells from each group was loaded on a Chromium controller (10X Genomics) following the protocol for 3' v3.1 dual index chemistry single cell RNA sequencing (CG000315_RevE and attached products, 10x Genomics). For the muscularis, cells sorted from the control *Il13ra1*^{flox} group (~10,000 sort-enriched cells pooled from 1 male and 1 female mouse) and the *Calb2*$^{\Delta Il13ra1}$ group (~10,000 sort-enriched cells pooled from 1 male and 1 female mouse) were loaded into two separate channels of the Chromium controller (10X Genomics). For epithelial samples, cells from the control *Il13ra1*^{flox} group (~5,000 manually counted cells from 1 female mouse; male sample compromised) and the *Calb2*$^{\Delta Il13ra1}$ group (~10,000 manually counted cells pooled from 1 male and 1 female mouse) were loaded into two separate channels, respectively, of the Chromium

controller (10X Genomics). Libraries were prepared according to manufacturer's instructions, pooled in equimolar ratios and sequenced on a NovaSeq sequencer (Illumina) in the following read configuration: R1:28, I1:10, I2:10, R2:90bp. After sequencing, BCL files were demultiplexed to individual libraries and aligned against the mouse reference genome (GRCm38 / mm10) with Cell Ranger v7.0.1 (10x Genomics) ran through the Cumulus pipeline (*39*) to generate count matrices.

Pre-processing and quality control—The data analysis was conducted using Scanpy (version 1.10.3) (*43*). The count matrices from the four samples (Control muscularis; *Calb2*$^{\Delta Il13ra1}$ cKO muscularis; Control epithelium; *Calb2*$^{\Delta Il13ra1}$ cKO epithelium) were concatenated, and doublets were detected using Scrublet (version 0.2.3) (*44*) and scDblFinder (version 1.18.0)(*45*) with an expected doublet rate of ~12%. The cells were excluded if they were classified as doublets, expressed ≤ 200 genes, or had ≥ 10 % of mitochondrial gene expression. Normalization was performed using median count depth, followed by log1p transformation. Highly variable genes (HVGs) were annotated for each sample.

Dimensionality Reduction and Clustering—Principal component analysis (PCA) was performed on the HVGs, and 30 principal components were used to compute the neighborhood graph with 15 neighboring data points. Two-dimensional Uniform Manifold Approximation and Projection (UMAP) (*46*) was calculated using the neighborhood graph for visualizing the data. Clustering was performed using the Leiden algorithm (*47*), at resolutions ranging from 0.1 to 2.0 (steps of 0.1). A cluster comprising < 0.1% of total cells at resolution 1.4 and consisting of cells solely from the control genotype group was removed.

Cluster annotation—Marker genes for 37 clusters at resolution of 1.4 were identified using the Wilcoxon rank-sum test implemented in the rank_genes_groups function. Clusters were annotated using the following sets of cell-type marker genes and assisted using the CellTypist (*48*, *49*) "Adult_Mouse_Gut" model and "Intestine" model:
Immune cells (*Ptprc*); Macrophages (*Ptprc, Itgax, Lyz2, Arg1, Chil3, Ccl24, H2-Ab1, H2-Aa, Mgl2, Retlna, C1qb, C1qa, Adgre1, Pf4, Cx3cr1, Apoe, Itgam*); Monocytes (*Ptprc, Ly6c2, Itgam*); Dendritic cells (DC) (*Ptprc, Zbtb46, Itgax, Itgae, Cd209a, H2-Ab1, H2-Aa*); Neutrophils (*Ptprc, S100a9, S100a8, Csf3r, Il1b, Itgam*); CD4+ αβT cells (*Ptprc, Cd4, Trac, Cd3g, Cd3d, Cd3e, Thy1*);

CD8+ αβT cells (*Ptprc, Cd8a, Trac, Cd3g, Cd3d, Cd3e, Thy1*); γδT cells (muscularis) (*Ptprc, Cd3g, Cd3d, Cd3e, Thy1, Tcrg-C1, Trdc, Il23r*); γδT cell intraepithelial lymphocytes (IEL) (from epithelium samples, *Ptprc, Cd3g, Cd3d, Cd3e, Itgae, Cd8a, Tcrg-C1, Trdc, Itgax*); Natural Killer (NK) cells (*Ptprc, Thy1, Nkg7, Gzmb, Klre1, Gzma, Eomes,* negative or low for: *Trac, Cd3g, Cd3d, Cd3e*); ILC2s (*Ptprc, Thy1, Il17rb, Gata3, Il1rl1, Il13, Il5, Arg1,* negative or low for: *Trac, Cd3g, Cd3d, Cd3e*); Glia (*S100b, Gfap, Plp1, Sox10, Prnp,* negative or low for: *Ptprc*); Fibroblasts (*Lum, Spon2, Dpt, Col1a1, Col1a2, Dcn, Pdgfra,* negative or low for: *Ptprc*); Myofibroblasts (*Kcnq5, Bmpr1b, Myl9, Mustn1, Acta2,* negative or low for: *Ptprc*); Interstitial cells of Cajal (ICC) (*Ano1, Kit, Adgrd1, Pcdh17,* negative or low for: *Ptprc*); Pericytes (*Pdgfrb, Vtn, Higd1b, Rgs5, Des, Cspg4,* negative or low for: *Ptprc*); Endothelial cells (*Pecam1, Ly6c1, Ptprb, Robo4, Aqp1, Tek, Cd34, Tie1, Dll4, F11r,* negative or low for: *Ptprc*); Epithelial cells (from epithelium samples, generally *Epcam*-positive and *Ptprc*-negative); Stem cells (epithelial) (*Olfm4, Mki67, Pcna*); Enterocytes and Transit Amplifying (TA) cells (*Pigr, Muc3, Mttp, Sprr2a3, Spink1, Fabp1, Fabp2, Vil1, Gsdmc4, Gsdmc2*); Goblet cells (*Rnase1, Muc2, Clca1, Retnlb, Tff3*); Paneth cells (*Lyz1, Defa3, Defa23, Defa22, Defa21, Defa36, Ang4*); Enteroendocrine cells (EEC) (*Cck, Chga, Chgb, Tac1, Nrg1*); Tuft cells (*Dclk1, Pou2f3, Trpm5, Alox5, Hck, Lrmp*)

For detailed annotation of the lymphoid cell clusters, the counts from four lymphoid clusters at resolution 1.4 were re-processed and re-clustered using the Leiden algorithm at resolutions ranging from 0.1 to 1.0 (steps of 0.1). Lymphoid clusters at resolution 0.9 were annotated using cluster level marker genes, identified using Wilcoxon rank-sum test method. These lymphoid cluster annotations were integrated into the fine level annotation of the clusters. Clusters with strong positive correlation in their gene expression profiles were grouped together, resulting in 29 clusters at fine level with total 8270 cells.

To lower the unnecessary complexity in our analyses, we used two levels of cluster annotation, the "fine" level cluster annotation with 29 clusters, described above, and the "general" level cluster annotation, where we grouped together clusters of the same general cell type, including the two clusters of macrophages, glia, fibroblasts, and TA cells. The general level cluster annotations yielded 23 general level clusters, including the 6 fine level lymphoid cell clusters. The level of clustering used for all scRNA-seq analyses is specified in the figure legends.

The fine level MMφ 2 cluster differed from the MMφ 1 in the expression of several glial cell marker genes (*S100b, Gfap, Plp1, Sox10, Prnp*) and the MMφ 1 cluster included cells from the

duodenal epithelium samples. To compare differences in only the macrophages of the muscularis, 24 cells from epithelium samples were removed from the general and fine MMφ clusters in all DEG analyses, clustering dot plots, and MMφ violin plots (i.e., **Fig. 4, G** and **H; Fig. 5B; Supplemental fig. S13B; Supplemental fig. S14, G-K;** and **Supplemental fig. S15, F** and **G**). However, the MMφ cells from the epithelial samples were included in all UMAPs and proportion charts (i.e., **Fig. 4F** and **Supplemental fig. S13, D-F**).

Sex prediction of cells—To predict the sex of individual cells, a trained model for the mouse genome from the R package cellXY (version 0.99.0) [Phipson Lab, https://github.com/phipsonlab/cellXY] was used. The classifySex function classified 6108 cells as female, 2037 cells as male and could not classify 125 cells as either, due to zero counts for Xist and sum of Y chromosome genes.

Differential gene expression analysis—Differentially expressed genes (DEGs) were identified for each genotype (Control and $Calb2^{\Delta Il13ra1}$ cKO) within each celltype using the Wilcoxon rank-sum test method. The cells were grouped by celltype (fine level), predicted sex and genotype and the counts for these groups were aggregated to perform pseudobulk analysis. The predicted sex was treated as replicate and DESeq2 (version 1.44.0)(*42*) was used to detect the DEGs, comparing Ctrl and KO groups. The DEGs were considered significant if |log2 fold change| > 0.58 and adjusted p-value < 0.05.

Gene signature scores

Custom gene sets were curated based on DEGs displayed in violin or volcano plots from specific experiments in Jarick *et al.* (*25*) and Pinho-Ribeiro *et al.* (*8*). For custom gene modules from Jarick *et al.*: "Up in Control Mφ" (*Arg1*, *Chil3*, *Ccl24*, *Rnase2a*, *Retnla*, *Chil1*, *Chia1*, *Lcn2*, *Slc26a4*) and "Up in $Nmur1^{Cre}$; $Id2^{fl/fl}$ Mφ" (*F13a1*, *Ms4a4a*, *Fcgr1*, *Socs3*, *C5ar1*, *Cd81*) represent genes with higher or lower expression, respectively, in Control lung macrophages and type 2 alveolar cells compared to those from $Nmur1^{iCre\text{-}eGFP}$; $Id2^{fl/fl}$ mice (i.e., ILC2-deficient mice). For custom gene modules from Pinho-Ribeiro *et al.*: "Up in CGRP Mφ" (*Jdp2*, *Crem*, *Tgfb3*, *Arg1*, *Nfkbia*, *Hif1a*, *Vegfb*) and "Down in CGRP Mφ" (*Tnf*, *Ccl5*, *Ccl3*, *Ccl4*, *Cxcl10*, *Ccl2*, *Ccl12*, *Ccl7*) represent genes with higher or lower expression, respectively, in bone

marrow-derived macrophages (BMDM) treated with both *S. pneumoniae* and CGRP compared to BMDM treated with *S. pneumoniae*, alone. Signature scores for gene sets on the general-level MMφ (muscularis only) were computed using AddModuleScore (*50*) function from R package Seurat (version 5.1.0)(*51*).

Gene set enrichment analysis (GSEA)

GSEA was performed using fgsea (version 1.30.0) (*52*) in R. Ranked genes were obtained using Wilcoxon rank sum test in rank_genes_groups function in scanpy for the genotype comparison (i.e., Control vs. *Calb2*$^{\Delta Il13ra1}$ cKO) in MMφ (muscularis cells only), as well as stromal fibroblast and glia clusters. Significantly expressed genes (|log2 fold change| > 0.58 and adjusted p-value < 0.05) ranked according to their test statistics and Gene Ontology, KEGG, and Reactome gene sets from msigdbr (version 7.5.1) in R [Dolgalev I (2024). *msigdbr: MSigDB Gene Sets for Multiple Organisms in a Tidy Data Format*. R package version 2023.1.1, https://igordot.github.io/msigdbr/.] were used to find the gene sets enriched in these cell populations comparing between Control and *Calb2*$^{\Delta Il13ra1}$ cKO cells.

Integration and reanalysis of published datasets

Pre-processing and integration of mouse enteric neuron datasets—The level 2 "l2_neurons_enteric.loom" loom file from Zeisel, *et al.* (*12*) was downloaded on approximately 02/25/2020 from mousebrain.org. Matrix, barcodes, and features files from P21 replicates (n = 2) of Morarach, *et al.* (*11*) were downloaded on approximately 04/22/2021 from the Gene Expression Omnibus (GEO) database with the identifier GSE149524 and accession number SRP258962. The metadata file and gene-sorted matrix, barcode, and gene files associated with "Mouse ileum neurons (10x)" (i.e., with prefix "msi.") from Drokhlyansky, Smillie, and Van Wittenberghe, *et al*. (*10*) were downloaded on 11/25/2022 from the Broad Institute Single Cell Portal. Data was loaded from Zeisel, *et al.* using the read_loom_dgCMatrix() and read_loom_anno() functions. Data was loaded from Drokhlyansky, Smillie, and Van Wittenberghe, *et al*. and each biological replicate from Morarach, *et al.* using the Read10x() function. Cells were filtered from each individual dataset as previously described (*11*). Briefly, cells with fewer than 1,500 genes or greater than 8,000 genes were removed and cells with greater than 60,000 UMI counts were

excluded to mitigate potential doublets. Next, we performed a graded filtering of mitochondrial gene percentage, removing cells with mitochondrial gene count percentages greater than 10% (for cells with fewer than 4,000 genes) and greater than 25% (for cells with greater than 8,000 genes). The expression data from each individual dataset was then normalized using the Gamma-Poisson generalized linear model implemented in the SCTransform() function (*53*, *54*), regressing out the contribution of mitochondrial gene percentage and retaining 3,000 variable genes. Next, the Zeisel, *et al.* dataset, the Drokhlyansky, Smillie, and Van Wittenberghe, *et al*. dataset, and the two Morarach, *et al.* replicate datasets were prepped and integrated on SCTransform-normalized data using the SelectIntegrationFeatures() (3,000 features), PrepSCTIntegration(), FindIntegrationAnchors(), and IntegrateData() functions (*55*). To aid subtype-specific clustering, prominent sex-specific genes (*Xist*, *Gm13305*, *Tsix*, *Eif253y*, *Ddx3y, Uty*) and several immediate early genes (*Fos*, *Jun*, *Junb*, *Egr1*) were excluded from variable genes prior to principal component analysis (PCA). We then constructed a first-level *k*-nearest neighbor graph (50 PCs) and performed clustering (resolution = 0.5) using the FindNeighbors() and FindClusters() functions, respectively. We excluded two glial cell clusters, which showed clear separation from enteric neurons populations on the basis of *Plp1*, *Sox10*, and *Gfap* expression, and retained the remaining clusters enriched in enteric neuron markers *Elavl4, Tubb3,* and *Snap25* (comprised of 6,377 neurons), which were subjected to second-level PCA, *k*-nearest neighbors analysis (50 PCs), and clustering (resolution = 0.5)—yielding 13 distinct clusters comparable to the 12-13 neuronal clusters annotated from each individual study (*10–12*). The RunUMAP() function was used for dimensionality reduction and visualization (50 PCs, default settings). For visualization of gene expression data, the RNA slot was log-normalized and scaled using the NormalizeData() and ScaleData() functions.

Supervised annotation of mouse enteric neuron clusters—Mouse enteric neuron clusters were named in a similar fashion to those in Drokhlyansky, Smillie, and Van Wittenberghe, *et al*. and using similar neuron subtype distinguishing markers as in Morarach, *et al*. These annotations infer putative neuronal subtypes based on reported marker gene expression only, which cannot, alone, be used to imply function. Briefly, enteric neurons were first broadly segregated into *Gfra2*$^{+}$ neurons (8 clusters)—which were largely cholinergic and generally expressed *Chat* and *Casz1*—

and *Etv1*$^{+}$ neurons (5 clusters)—which were largely nitrergic and generally expressed *Nos1* and *Gfra1*.

Five putative excitatory motor neuron (PEMN) clusters were *Gfra2*$^{+}$ cholinergic neurons and distinguished from non-motor neurons by *Chat* and *Tac1* expression and the absence of *Nos1*, *Calcb*, and *Nmu*. The only enteric neuron cluster not represented by all 3 datasets was PEMN2, which only included neurons from Zeisel, *et al*. and Morarach, *et al*. Both PEMN1 and PEMN2 expressed *Calb2* and *Ndufa4l2* with low/negative expression of *Penk* and *Gda* (corresponding to ENC1 of Morarach, *et al*.), while PEMN3 expressed all these marker genes (corresponding to ENC2 of Morarach, *et al*.). On the other hand, PEMN4 and PEMN5 expressed *Penk* and *Gda* with low/negative expression of *Calb2* and *Ndufa4l2*, with PEMN5 displaying exclusive expression of *Fut9*—corresponding to ENC3 and ENC4 of Morarach, *et al*., respectively. Two putative inhibitory motor neuron (PIMN) clusters were *Etv1*$^{+}$*Nos1*$^{+}$ nitrergic neurons—one cluster with high expression of *Npy* and the other with lower or no expression of *Npy* and high expression of *Adm*, corresponding to ENC8 and ENC9 of Morarach, *et al*., respectively.

Putative sensory neurons (PSN)/IPANs corresponded to representative clusters with probabilistic neuronal subtype assignment of "IPAN/INs" from Morarach, *et al*.: PSN (*Nmu*$^{+}$) displayed generally exclusive expression of *Nmu, Nog, Atoh8,* and *Dlx3*, and showed high expression of *Calcb*, *Calb2*, *Ngfr*, *Cux2*, and *Ano2*—corresponding to ENC6 of Morarach, *et al*. PSN (Vglut2^{+}*Calb1*$^{+}$) expressed *Calb1, Slc17a6, Vip, Npy1r,* and *Nefm*—corresponding to ENC12 of Morarach, *et al*. The single cluster annotated as putative intestinofugal neurons (PIFN) was enriched in *Cck*, *Nefm*, *Vip*, and *Slc17a6*—corresponding to ENC7 of Morarach, *et al*. and PSN3 in Drokhlyansky, Smillie, and Van Wittenberghe, *et al*.

The three putative interneuron (PIN) clusters were annotated by their exclusive or high expression of several distinguishing markers: PIN (*Gal*$^{+}$*Gad2*$^{+}$) neurons expressed *Gal*, *Gad2* and *Neurod6*, PIN (*Sst*$^{+}$) neurons expressed *Sst* and *Vipr2*, and PIN (*Th*$^{+}$*Vip*$^{+}$*Npy*$^{+}$) neurons expressed *Th, Dbh,* and *Npy*—loosely representing ENC10, ENC5, and ENC11 from Morarach, *et al*., respectively, or PSVN1 , PSN4, and PSVN2 from Drokhlyansky, Smillie, and Van Wittenberghe, *et al*., respectively. Despite the possibility of PIN populations being misannotated as interneurons and their disagreement with neuron subtype annotations between different datasets, we chose to call them PINs based on their probabilistic neuronal subtype and proposed functional assignment as interneuron ("INs") or sensory neuron/interneuron ("IPANS/INs") from Morarach, *et al*. (*11*).

Pre-processing of human enteric neuron dataset—The gene-sorted matrix, barcode, and gene files associated with human colon enteric neuron single nuclei RNA-sequencing (snRNA-seq) (i.e., with prefix “hli.neur”) from Drokhlyansky, Smillie, and Van Wittenberghe, *et al*. (*10*) were downloaded on 06/26/2023 from the Broad Institute Single Cell Portal. Cells with fewer than 200 genes or greater than 8,000 genes were removed and cells with greater than 60,000 UMI counts were excluded to mitigate potential doublets. Expression data was next log-normalized and scaled in Seurat (version 4.3.0), and then PCA, *k*-nearest neighbors analysis (50 PCs) and clustering (resolution = 0.5) was performed. Cells and clusters were annotated as in Drokhlyansky, Smillie, and Van Wittenberghe, *et al*. and the log-normalized, scaled RNA slot was used to visualize gene expression.

Statistical analyses

Statistical analysis of data was performed on GraphPad Prism software (versions 8 to 9.5.1). Unless otherwise specified in the figure legends or main text, bar plots and scatter dot plots are presented as the mean ± SEM and box and whisker plots are presented as the minimum, 1st quartile, median, 3rd quartile, and maximum. For some box and whisker plots, a ‘+’ symbol designates the mean. Statistical significance in plots comparing only two groups was determined using unpaired two-tailed t-tests or non-parametric two-tailed Mann-Whitney tests. However, paired t-tests were performed where appropriate based on experimental design. Normality for two group comparisons was calculated using the D'Agostino & Pearson, Anderson-Darling, and/or Shapiro-Wilk tests to determine whether data should be analyzed with non-parametric tests (i.e., Mann-Whitney).

Analyses comparing three or more distinct groups with one independent variable were determined using one-way Analysis of Variance (ANOVA) with multiple comparisons. One-way ANOVA comparing between all column factors or comparing all columns against a control column were corrected using Tukey’s or Dunnett’s test, respectively. Analyses comparing multiple groups with two independent variables were determined using two-way ANOVA with multiple comparisons, and repeated measures/matching of subjects was performed where appropriate based on experimental design. 2way ANOVAs with multiple comparisons between column factors or between both column and row factors were corrected using Sidak’s and Tukey’s test, respectively.

P-values from multiple comparisons are indicated on plots. P values < 0.05 are considered significant (‘ns’, not significant; ‘*’, $p < 0.05$; ‘**’, $p < 0.01$; ‘***’, $p < 0.001$; ‘****’, $p < 0.0001$).

Supplemental fig. S1

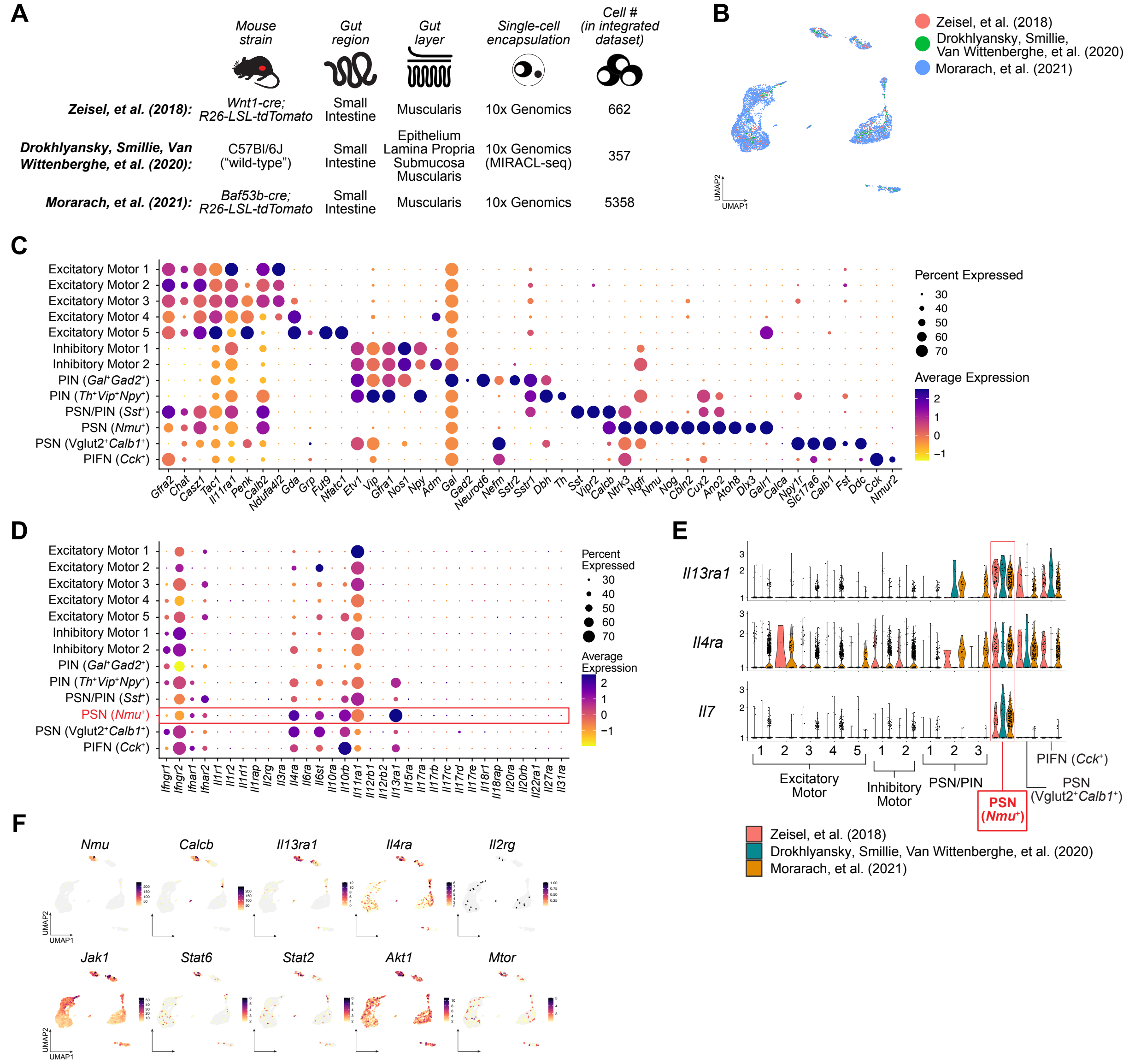

Supplemental fig. S1: Integration of enteric neuron scRNA-seq datasets.

(A and **B)** Single cell RNA-seq analysis of enteric neurons from the mouse small intestine. Data was integrated from three independent studies—Zeisel et al. (2018); Drokhlyansky, Smillie, Van Wittenberghe, et al. (2020); and Morarach et al. (2021). (A) Schematic table of the mouse strain, gut region, gut layer, single-cell methodology, and cell number of each individual dataset. (B) UMAP depicting integration of cells from each dataset (represented by different colors). **(C)** Dot plot of integrated dataset depicting marker genes used to distinguish annotated clusters; percent expressed dot size cutoff, min = 25% and max = 75%. **(D)** Dot plot of integrated dataset depicting various cytokine receptor genes in annotated enteric neuron clusters; percent expressed dot size cutoff, min = 25% and max = 75%. **(E)** Violin plots of gene expression in enteric neuron clusters, split and colored by original dataset. **(F)** UMAP feature plots showing expression of indicated genes in the integrated dataset.

Supplemental fig. S2

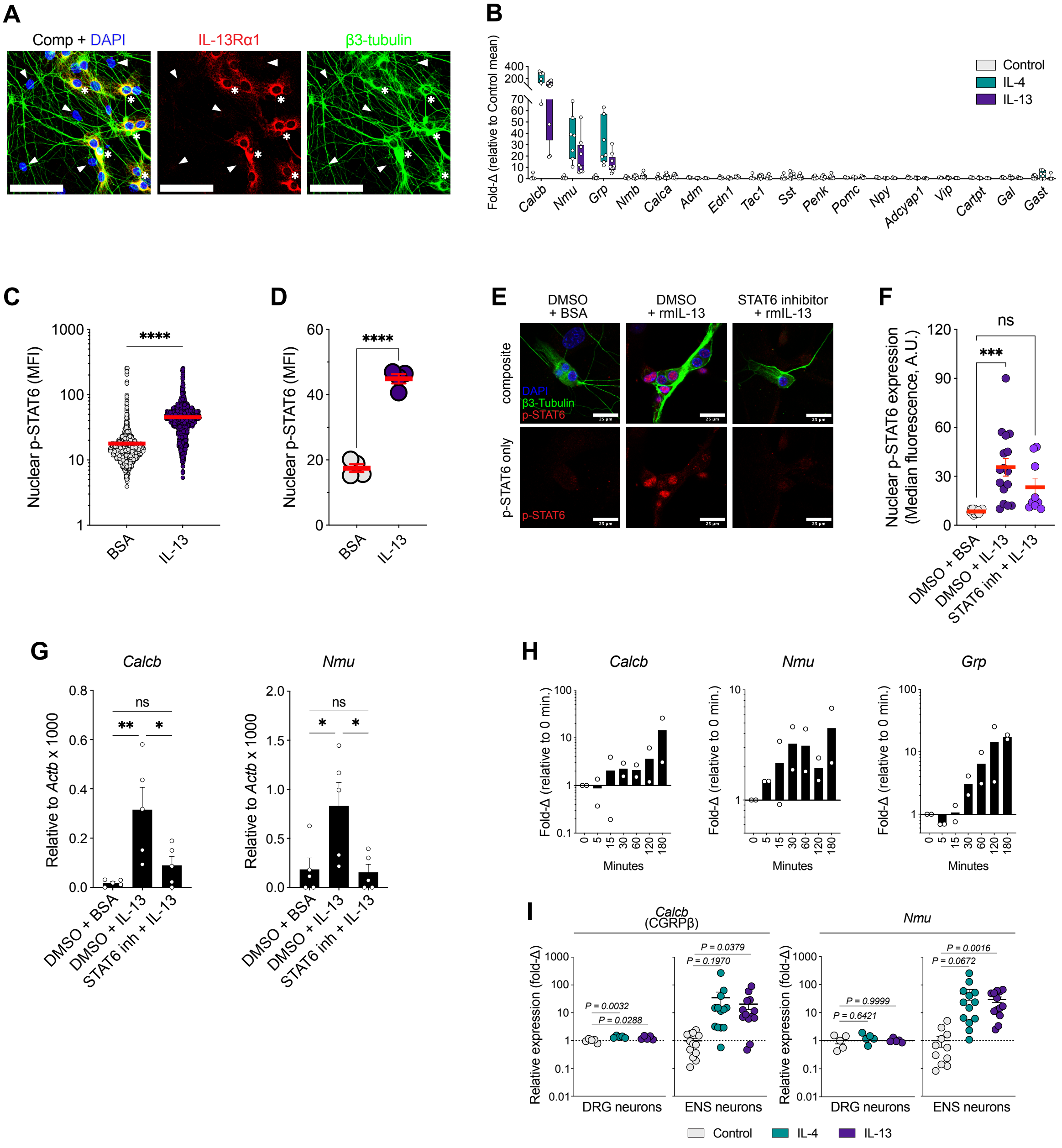

Supplemental fig. S2: Enteric neuro-glial cultures and neuropeptide expression.
In vitro enteric neuro-glial cultures differentiated from neonatal enteric neuro-glial progenitors. **(A)** Representative confocal microscopy images of cultures immunostained for IL-13Rα1 (red), β3-tubulin (green), and stained with DAPI (blue). Asterisks indicate enteric neurons (β3-tubulin^{+}) with IL-13Rα1^{+} cell bodies. Arrowheads indicate DAPI^{+}β3-tubulin^{neg} non-neuronal cells. Scale bar = 50µm. **(B)** Cultures were treated with either IL-4 (50 ng mL^{-1}), IL-13 (50 ng mL^{-1}), or BSA vehicle (Control) for 24 hours and processed for bulk RNA-seq analysis. DESeq2-normalized count data for each indicated gene is represented as the fold-change relative to the Control group mean. Each datapoint represents an *in vitro* culture well derived from an individual mouse (*n* = 7 to 9 mice). **(C and D)** Cultures were treated with IL-13 (50 ng mL^{-1}) or BSA (0.0025%) for 24-hours and nuclear phospho-STAT6 was assessed by confocal imaging. Quantification of nuclear phospho-STAT6 median fluorescence (C) per neuron (*n* = 1,363-1,481 neurons) and (D) per *in vitro* culture well derived from individual mice (*n* = 4 mice). **(E-G)** Cultures were pre-treated for 30 min. with either DMSO or STAT6 inhibitor (AS1517499, 250nM) prior to 24-hour incubation with IL-13 (50 ng mL^{-1}) or BSA (0.0025%). (E) Representative confocal microscopy images; DAPI, β3-tubulin, and phospho-STAT6 (Tyr641) is pseudocolored blue, green, and red, respectively; scale bars = 25µm. (F) Quantification of nuclear phospho-STAT6 median fluorescence per neuron; each datapoint represents a single neuron (*n* = 9-16 neurons from cultures pooled from 8-10 mice). (G) qPCR gene expression ($2^{-\Delta Ct}$ normalized to *Actb* multiplied by 1,000) of cultures treated with indicated conditions. Each datapoint represents an *in vitro* culture well derived from an individual mouse (*n* = 5 mice). Data (C-G) represents 1 of ≥2 independent experiments. **(H)** Time-course of qPCR gene expression in enteric neuro-glial cultures after administration of IL-4 (50 ng mL^{-1}). Gene expression is relative to *Actb* ($2^{-\Delta Ct}$) and represented as fold-change to the 0 min. time-point (*n* = 2 cultures from individual mice). **(I)** qPCR gene expression from DRG neurons and enteric neuro-glial cultures. Data is represented as fold-change to BSA control group. For DRG cultures, each datapoint represents different wells from cultures of DRGs pooled from 2 mice (*n* = 5 wells); for enteric neuro-glial cultures, each datapoint represents an *in vitro* culture well derived from an individual mouse per condition (*n* = 11-13 mice). Each bar/scatter plot indicates the mean ± SEM of replicates. ns = not significant, **P* < 0.05, ***P* < 0.01, ****P* < 0.001, *****P* < 0.0001 using two-tailed t test (C, D) or one-way ANOVA with Dunnet's (F, I) or Tukey's (G) correction.

Supplemental fig. S3

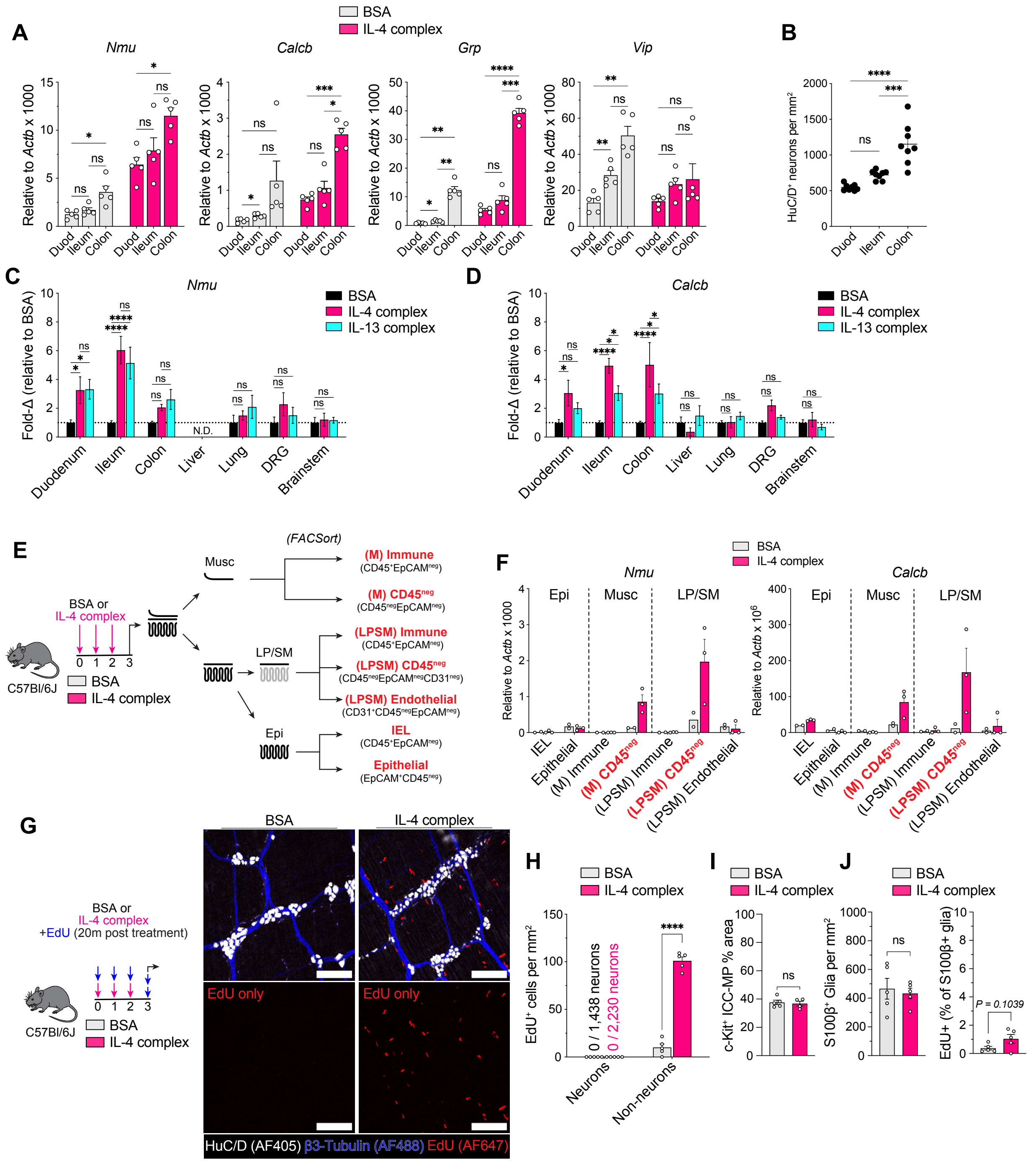

Supplemental fig. S3: Regional effects of type 2 cytokine-antibody complexes
(A) qPCR gene expression of indicated genes in the duodenum (Duo), ileum, and colon from mice of **Fig. 1G**. C57Bl/6J administered (i.p) IL-4 complex (IL-4:anti-IL4, 1μg:5μg per dose) or BSA for 3 days and euthanized 24h following the final dose; gene expression is relative to *Actb* ($2^{-\Delta Ct}$) multiplied by 1,000. Data represent 1 of >2 independent experiments and each datapoint represents a single mouse ($n = 5$ mice). **(B)** Wholemount confocal imaging of myenteric plexus neurons of mice from **Supplemental fig. S4, C-M**. Quantification of $HuCD^+$ enteric neuron cell bodies in different regions of the small intestine. Data pooled from 3 independent experiments and each datapoint represents a single mouse ($n = 8$ mice). **(C** and **D)** Female C57Bl/6J mice were administered (i.p) IL-4 complex (IL-4:anti-IL-4, 1μg:5μg per dose), IL-13 complex (IL-13:anti-IL-13, 1μg:5μg per dose), or BSA for 3 days and euthanized 24h following the final dose. qPCR gene expression for (C) *Nmu* and (D) *Calcb* detected in the duodenum, ileum, colon, liver, lung, DRG, and brainstem. Data is normalized to *Actb* ($2^{-\Delta Ct}$) and represented as fold-change to the BSA group for each organ. Each datapoint represents a single mouse ($n = 5$ mice). **(E** and **F)** Co-housed, female C57Bl/6J mice were administered (i.p) IL-4 complex (IL-4:anti-IL-4, 1μg:5μg per dose) for 3 days and euthanized 24h following the final dose. Duodenal muscularis and lamina propria/submucosa (LPSM) were separated and epithelium was denuded from the LPSM, and the epithelium, LPSM, and muscularis were separately processed into single-cell suspensions. (E) Schematic of sorting strategy. Indicated cell populations were FACS-purified and lysed for RNA analysis. (F) qPCR gene expression of *Nmu* (left) and *Calcb* (right) in indicated FACS-purified populations. Data is normalized to *Actb* ($2^{-\Delta Ct}$) and multiplied by 1,000 ($n = 2$-3 mice). **(G-J)** Co-housed, female C57Bl/6J mice were administered (i.p) IL-4 complex (IL-4:anti-IL-4, 1μg:5μg per dose) for 3 days and euthanized 24h following the final dose. Mice were also administered (i.p.) EdU (100μg per mouse per injection) 20 min. following each IL-4 complex dose, as well as 2 hours and 30 min. prior to euthanasia. (G) Schematic (left) and representative wholemount confocal image of duodenal myenteric plexus (right) immunostained for HuCD and β3-tubulin (pseudocolored white and blue, respectively). EdU was detected with AF647 (pseudocolored red) using the Click-iT Plus Cell Proliferation Assay (scale bars = 100μm). (H) Quantification of EdU+ neurons ($HuCD^+$) and non-neurons ($HuCD^{neg}$) per mm^2, (I) percent c-kit^+ area, and (J) number of glial cells ($S100\beta^+$) per mm^2 (left) and percent of EdU^+ glial cells ($S100\beta^+$) (right). Data represent 1 of 2 independent experiments and each datapoint represents a single mouse ($n = 5$ mice). Each graph indicates the mean ± SEM of replicates. N.D. = not detected, ns = not significant, $*P < 0.05$, $**P < 0.01$, $***P < 0.001$, $****P < 0.0001$ using 2way ANOVA with Sidak's correction (A, C, D, H), one-way ANOVA with Tukey's correction (B), and two-tailed Welch's t-test (I, J).

Supplemental fig. S4

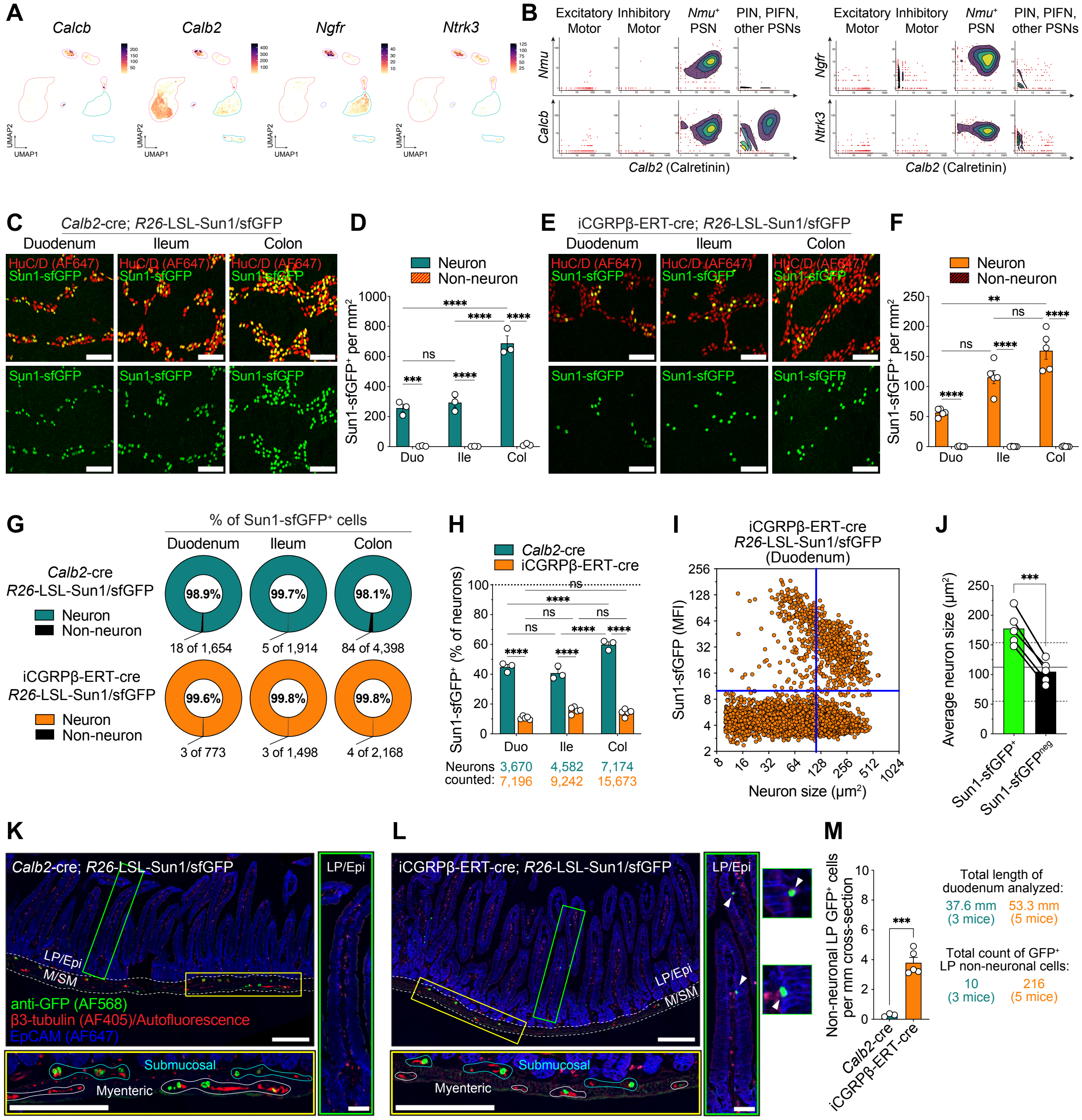

Supplemental fig. S4: Validation of *Calb2*-cre and iCGRPβ-ERT-cre mouse models.
(A and **B)** Integrated enteric neuron single cell RNA-seq data from Zeisel et al. (2018); Drokhlyansky, Smillie, Van Wittenberghe, et al. (2020); and Morarach et al. (2021). (A) UMAPs of enteric neuron clusters depicting gene expression of Nmu^{+} PSN markers (*Calcb, Calb2, Ngfr, Ntrk3*). (B) Scatter plots depicting indicated gene co-expression in combined excitatory motor neuron clusters, combined inhibitory motor neurons, Nmu^{+} PSNs, and combined PINs, PIFNs, and non-Nmu^{+} PSNs. **(C-J)** Wholemount confocal imaging of the duodenal, ileal, and colonic myenteric plexi from *R26-LSL-Sun1-sfGFP* nuclear reporter mice crossed to the *Calb2*-cre (C and D) and iCGRPβ-ERT-cre (E and F) mouse models. Sun1-sfGFP and HuCD (neuron nuclei/cell body marker) are pseudocolored green and red, respectively. Scale bar = 100 μm. **(C)** Representative images from *Calb2*-cre; *R26-LSL-Sun1-sfGFP* nuclear reporter mice and **(D)** quantification of Sun1-sfGFP+ neurons ($HuCD^{+}$) and non-neurons ($HuCD^{neg}$) per mm^2. Datapoints represent individual mice (*n* = 3 mice) pooled from 2 independent experiments. **(E)** Representative images from iCGRPβ-ERT-cre; *R26-LSL-Sun1-sfGFP* nuclear reporter mice and **(F)** quantification of Sun1-sfGFP+ neurons ($HuCD^{+}$) and non-neurons ($HuCD^{neg}$) per mm^2. Datapoints represent individual mice (*n* = 5 mice). **(G)** Pie-charts indicating the percentage of Sun1-sfGFP+ cells that are neurons and non-neurons in different regions of the gut. Numbers under pie-charts indicate the total non-neuronal Sun1-sfGFP+ cells counted out of all Sun1-sfGFP+ cells counted per gut-region per experiment. **(H)** Quantification of the percentage of all $HuCD^{+}$ myenteric plexi neurons that are Sun1-sfGFP+ from the two cre models across different regions of the gut. Numbers under bar graph represent total number of enteric neurons counted across all mice per experimental group per gut-region. Data pooled from 3 independent experiments (*n* = 3-5 mice). **(I)** Scatter-plot of individual $HuCD^{+}$ myenteric plexus neurons from the duodena of iCGRPβ-ERT-cre; *R26-LSL-Sun1-sfGFP* nuclear reporter mice (*n* = 7,196 neurons) comparing cell body size (i.e., $HuCD^{+}$ area in μm^2) to average intensity of Sun1-sfGFP fluorescence. Blue lines indicate average size of all neurons plotted (112.8 μm^2) and threshold for Sun1-sfGFP+ cells in experiment (MFI = 10 A.U.). **(J)** Quantification of average neuron size for GFP+ and GFP-negative cells. Dotted lines represent the first and third quartiles and the solid line represents the mean (*n* = 5 mice). **(K-M)** Representative confocal images of duodenal cross-sections from *Calb2*-cre; *R26-LSL-Sun1-sfGFP* (K) and iCGRPβ-ERT-cre; *R26-LSL-Sun1-sfGFP* (L) mice. EpCAM (epithelial cells), β3-tubulin (pan-neuron marker) + autofluorescence, and Sun1-sfGFP stained with anti-GFP (reporter) are pseudocolored blue, red, and green, respectively. Scale bar = 200 μm. Yellow insets focus on submucosal plexus and myenteric plexus (scale bar = 200 μm) and green insets focus on representative villi (scale bar = 50 μm). (M) Quantification of non-neuronal GFP+ cells in the lamina propria of indicated nuclear reporter mice. Datapoints represent individual mice (*n* = 3-5) pooled from 3 independent experiments. Each graph indicates the mean ± SEM of replicates. $*P < 0.05$, $**P < 0.01$, $***P < 0.001$, $****P < 0.0001$ using 2way ANOVA with Tukey's correction (D, F, H), Paired t test (J), and unpaired t test (M).

Supplemental fig. S5

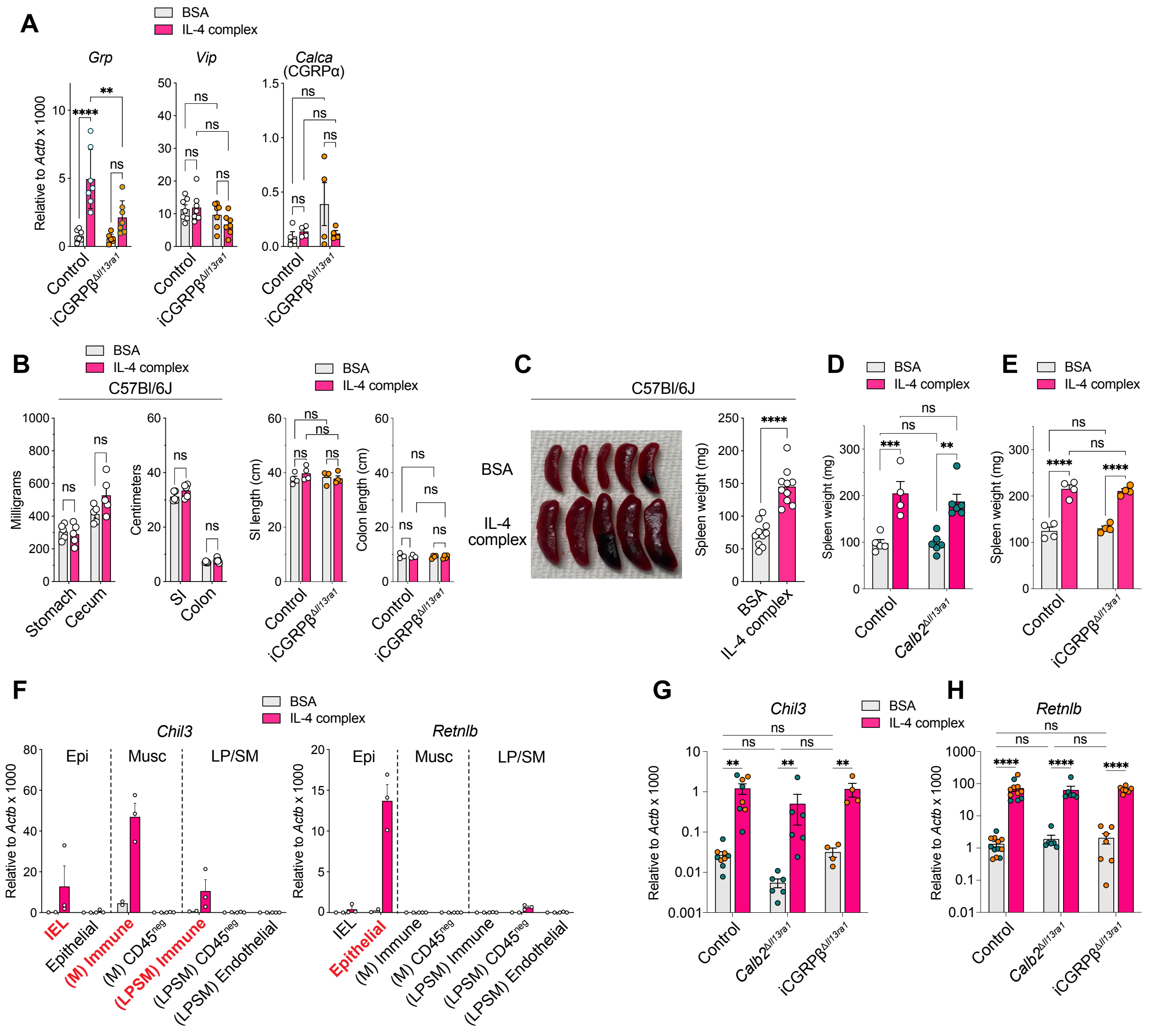

Supplemental fig. S5: Non-neuronal effects of type 2 cytokine-antibody complexes
(A) Co-housed, mixed sex iCGRPβ$^{\Delta Il13ra1}$ cKO and littermate control mice were orally gavaged 3 or 5 consecutive doses of tamoxifen and administered IL-4 complex for 4 days, starting a week after the first tamoxifen gavage, as in **Fig. 1J**; mice were euthanized 1h after final IL-4 complex injection. Duodenal qPCR gene expression for indicated genes relative to *Actb* ($2^{-\Delta Ct}$) multiplied by 1,000. Data either represents 1 of 2 independent experiments (*Calca*, n = 4) or pooled from 2 independent experiments (*Grp*, *Vip*, n = 7 mice). **(B)** Female C57Bl/6J (left) and mixed sex iCGRPβ$^{\Delta Il13ra1}$ cKO mice (right) treated with IL-4 complex or BSA as in **Fig. 1F** and **Fig. 1J**, respectively. Quantification of stomach and cecum weights in milligrams, or small intestine and colon length in centimeters. Data represents 1 of >3 (left, n = 5 mice) or 2 (right, n = 4 mice) independent experiments. **(C-E)** Representative image (C, left) and quantification of spleen weights in (C) C57Bl/6J mice, (D) *Calb2*$^{\Delta Il13ra1}$ cKO and littermate control mice, and (E) iCGRPβ$^{\Delta Il13ra1}$ cKO and littermate control mice receiving IL-4 complex or BSA treatment as in **Fig 1 F**, **H**, and **J**, respectively. Data is pooled from 2 independent experiments (C, n = 10 mice; D, n = 4-6 mice) or represents 1 of 2 independent experiments (E, n = 4). **(F)** qPCR gene expression for *Chil3* (left) and *Retnlb* (right) in indicated FACS-purified populations described in **Supplementary fig. S3E**. Data is normalized to *Actb* ($2^{-\Delta Ct}$) and multiplied by 1,000 (n = 2-3 mice). **(G and H)** Duodenal qPCR gene expression for (G) *Chil3* and (H) *Retnlb* in *Calb2*$^{\Delta Il13ra1}$ cKO (teal), iCGRPβ$^{\Delta Il13ra1}$ cKO (orange), and respective littermate control mice administered IL-4 complex or BSA. Data is normalized to *Actb* ($2^{-\Delta Ct}$) and multiplied by 1,000. Data is pooled from 3 (G, n = 4-9 mice) or 4 (H, n = 6-12) independent experiments. All datapoints represent individual mice. Each graph indicates the mean ± SEM of replicates. ns = not significant, *P < 0.05, **P < 0.01, ***P < 0.001, ****P < 0.0001 using 2way ANOVA with Tukey's correction (A, B, D, E, G, H) and unpaired t test (C).

Supplemental fig. S6

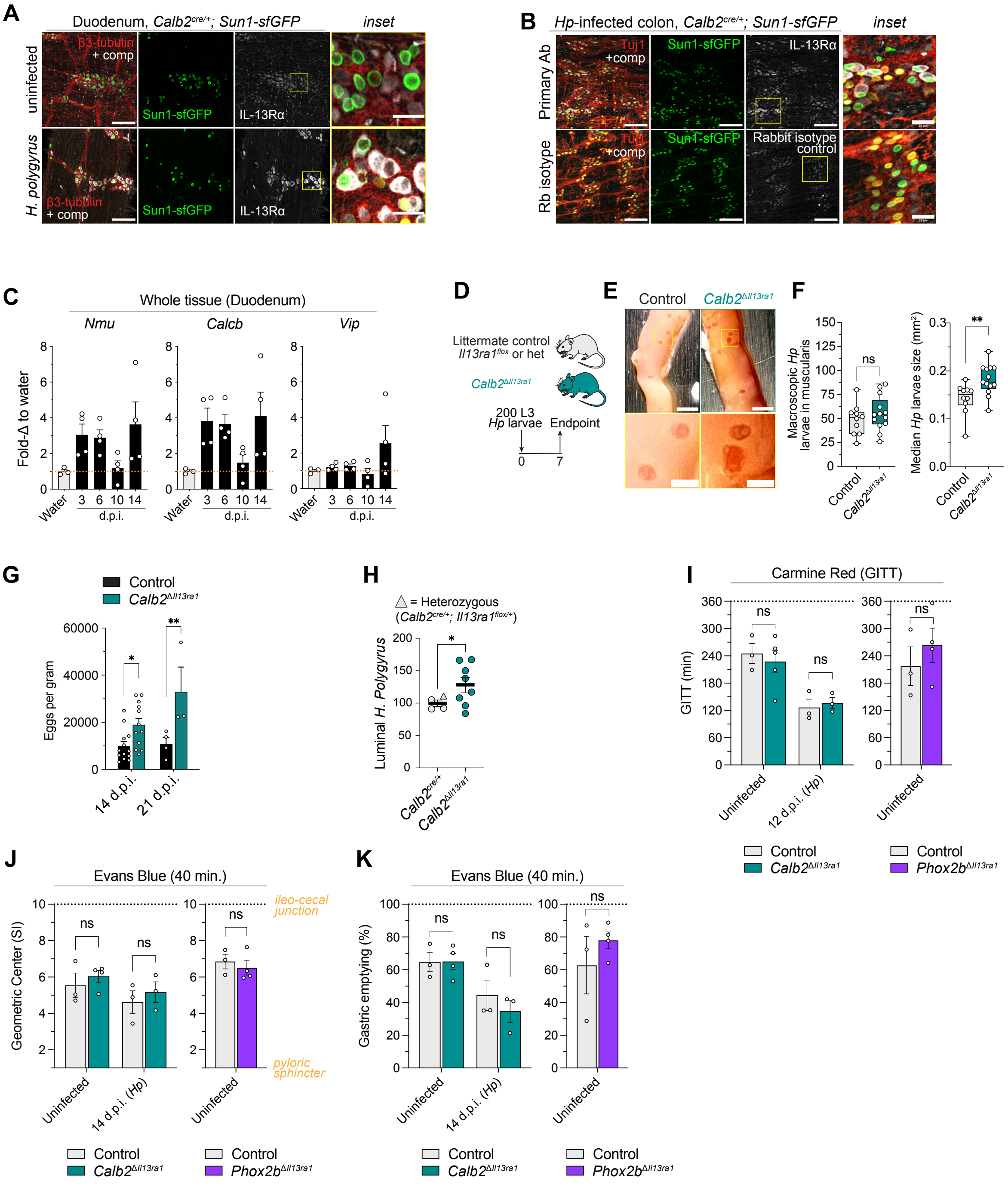

Supplemental fig. S6: *H. polygyrus* clearance deficits in *Calb2*$^{\Delta Il13ra1}$ cKO mice

(A and **B)** Whole-mount imaging of colon myenteric plexus neurons from naïve and *H. polygyrus*-infected (21 d.p.i.) *Calb2-cre; R26-LSL-Sun1/sfGFP* nuclear reporter mice. Representative images of (A) duodenal myenteric plexi from naïve (top) and 21 d.p.i. (bottom) mice stained with anti-IL-13Rα antibody, and (B) colonic myenteric plexi at 21 d.p.i. stained with anti-IL-13Rα antibody (top) or rabbit isotype control (bottom). β3-tubulin (pan-neuron marker), Sun1-sfGFP, IL-13Rα, and rabbit isotype control are pseudocolored red, green, white, and white respectively. Scale bars for field of view and inset (yellow box) are 100μm and 25μm, respectively. **(C)** qPCR of duodenal *Nmu, Calcb,* and *Vip* expression in naïve (Water) and *H. polygyrus* infected mice at different timepoints (d.p.i.: 3, 6, 10, 14); gene expression is relative to *Actb* ($2^{-\Delta Ct}$) multiplied by 1,000. Data is pooled from 5 independent experimental timepoints (*n* = 3-4 mice per timepoint). **(D-F)** Co-housed, mixed sex *Calb2*$^{\Delta Il13ra1}$ cKO and littermate control (*Il13ra1*^{flox}) mice were infected with 200 L3 larvae and euthanized at 7 d.p.i. (C) Schematic. (D) Representative photo of implanted worms (scale bars of field of view and inset are 250mm and 75mm, respectively) and (E) quantification of total number of implanted larvae (left) and median area of implanted larvae (right) per mouse; data pooled from 2 blinded independent experiments (*n* = 10-12 mice). **(G)** Eggs per gram feces at 14 d.p.i. (data from **Fig. 2G**) and 21 d.p.i. Data is pooled from 3 independent experiments (*n* = 13, 12, 4, 3 mice). **(H)** Co-housed, mixed sex *Calb2*$^{\Delta Il13ra1}$ cKO and littermate *Calb2*$^{cre/+}$ or heterozygous (∆) control mice were infected with 200 L3 larvae and euthanized at 14 d.p.i. Quantification of adult *H. polygyrus* in lumen. Data from a single experiment comparing only littermate *Calb2*$^{cre/+}$ and heterozygous (∆) controls with *Calb2*$^{\Delta Il13ra1}$ cKO mice (*n* = 4-8 mice). **(I-K)** Gastrointestinal motility and transit assays in naïve *Calb2*$^{\Delta Il13ra1}$ cKO, *Phox2b*$^{\Delta Il13ra1}$ cKO, and littermate control mice and in *H. polygyrus*-infected (12 d.p.i. or 14 d.p.i.) *Calb2*$^{\Delta Il13ra1}$ cKO and littermate control mice. (I) Total body gastrointestinal transit time (GITT) was measured by latency between first observed red fecal pellet after Carmine Red oral gavage. (J) Small intestinal transit of Evans Blue dye 40 min. after oral gavage. Data represents the geometric center of Evans Blue dye distribution across 10 equal segments of the small intestine, from 0 (closest to pyloric sphincter) to 10 (closest to the ileo-cecal junction). (K) Gastric emptying was measured 40 min. after Evans Blue oral gavage, represented as the amount of dye found in the small intestine as a percentage of the total dye in the stomach and small intestine. Data are composed of 3 independent experiments (*n* = 3-5 mice per group). Each datapoint represents a distinct mouse and each graph indicates the mean ± SEM of replicates. ns = not significant, $*P < 0.05$, $**P < 0.01$ using 2way ANOVA with Sidak's correction (G) and two-tailed Welch's t test (F, H-K).

Supplemental fig. S7

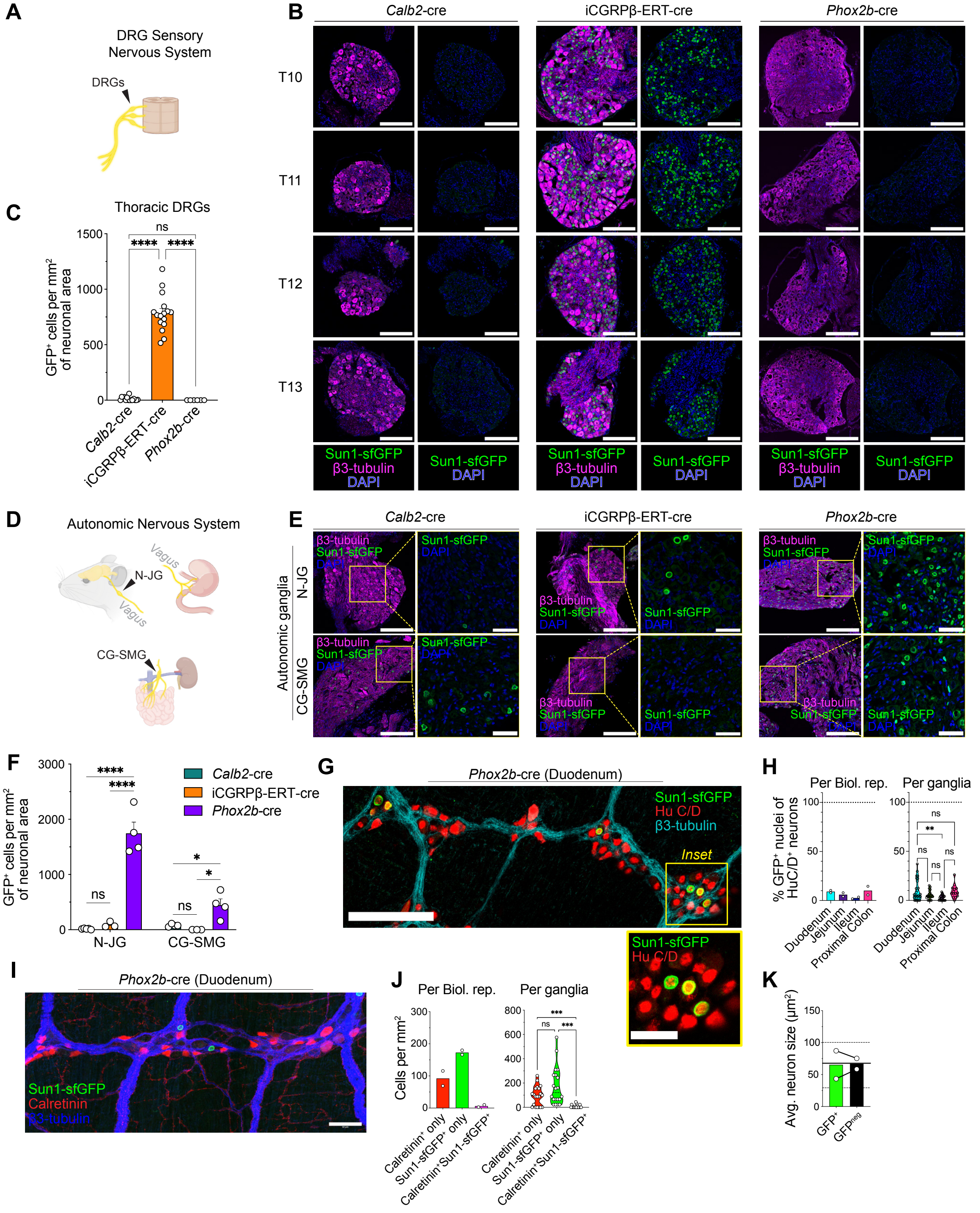

Supplemental fig. S7: Characterization of gut-extrinsic ganglia from *Calb2*-cre, iCGRPβ-ERT-cre, and *Phox2b*-cre mice.

(A-C) Confocal microscopy of the dorsal root ganglia (DRG) that comprise the thoracic splanchnic nerve innervating the proximal small intestine. (A) Schematic of DRG sensory system. (B) Representative images of DRGs from *R26-LSL-Sun1-sfGFP* nuclear GFP reporter mice crossed with *Calb2-cre* (left), iCGRPβ-ERT-cre (middle, 1 week after beginning 3 doses of tamoxifen), and *Phox2b-cre* (right) mice. β3-tubulin (pan-neuron marker), Sun1-sfGFP (nuclear reporter), and DAPI are pseudocolored magenta, green, and blue, respectively. Scale bars are 200 µm. (C) Quantification of GFP+ nuclei per mm^2 of β3-tubulin area in individual thoracic DRGs (pooled T10-T13) from *R26-LSL-Sun1-sfGFP* mice crossed into *Calb2-cre* (n = 15 DRG from 3 mice), iCGRPβ-ERT-cre (n = 16 DRG from 4 mice, 1 week after tamoxifen induction), and *Phox2b-cre* (n = 6 DRG from 2 mice) mice. Each datapoint represents an individual DRG pooled from multiple mice across 4 independent experiments. **(D-F)** Confocal microscopy of the Nodose-Petrosal-Jugular Ganglia (N-JG) of the sensory vagus system and the Celiac Ganglia-Superior Mesenteric Ganglia (CG-SMG) of the sympathetic nervous system. (D) Schematic of gut-innervating autonomic ganglia. (E) Representative images of N-JG (top) and CG-SMG (bottom) from *R26-LSL-Sun1-sfGFP* reporter mice crossed into indicated cre mouse models. β3-tubulin (pan-neuron marker), Sun1-sfGFP (nuclear reporter), and DAPI are pseudocolored magenta, green, and blue, respectively. Scale bars for field of view and inset (yellow box) are 200µm and 50µm, respectively. (F) Quantification of average GFP+ nuclei per mm^2 of β3-tubulin area from N-JG and CG-SMG of indicated reporter mouse strains. Each datapoint represents an individual mouse and pooled from 4 independent experiments. **(G-K)** Wholemount confocal imaging of the duodenal myenteric plexus from *Phox2b-cre; R26-LSL-Sun1-sfGFP* nuclear reporter mice. (G) Representative image of Sun1-sfGFP expression in myenteric ganglia enteric neurons. Sun1-sfGFP, Hu C/D (neuron nuclei/cell body marker), and β3-tubulin (pan-neuron marker) are pseudocolored green, red, and cyan, respectively. Scale bars for field of view and inset (yellow box) are 100µm and 30µm, respectively. (H) Quantification of the number of GFP^+ neuronal nuclei as a percentage of all sampled Hu C/D neurons across different regions of the gut per biological replicate (left, $n = 2$ mice) and per contiguous ganglia/field of view (right, n = 14-27 ganglia from 2 mice). (I) Representative image of Sun1-sfGFP expression in myenteric ganglia enteric neurons. Sun1-sfGFP, Calretinin, and β3-tubulin (pan-neuron marker) are pseudocolored green, red, and blue, respectively; scale bars are 50µm. (J) Quantification of $Calretinin^+$, $Sun1\text{-}sfGFP^+$, and double-positive cells per $β3\text{-}tubulin^+$ neuronal area per biological replicate (left, $n = 2$ mice) and per contiguous ganglia/field of view (right, $n = 20$ ganglia from 2 mice). (K) Quantification of average neuron size for GFP+ and GFP-negative cells. Dotted lines represent the first and third quartiles and the solid line represents the mean ($n = 2$ mice). Each graph indicates the mean ± SEM of replicates. $^*P < 0.05$, $^{**}P < 0.01$, $^{***}P < 0.001$, $^{****}P < 0.0001$ using one-way ANOVA with Tukey's correction (C, H, J) or 2way ANOVA with Tukey's correction (F).

Supplemental fig. S8

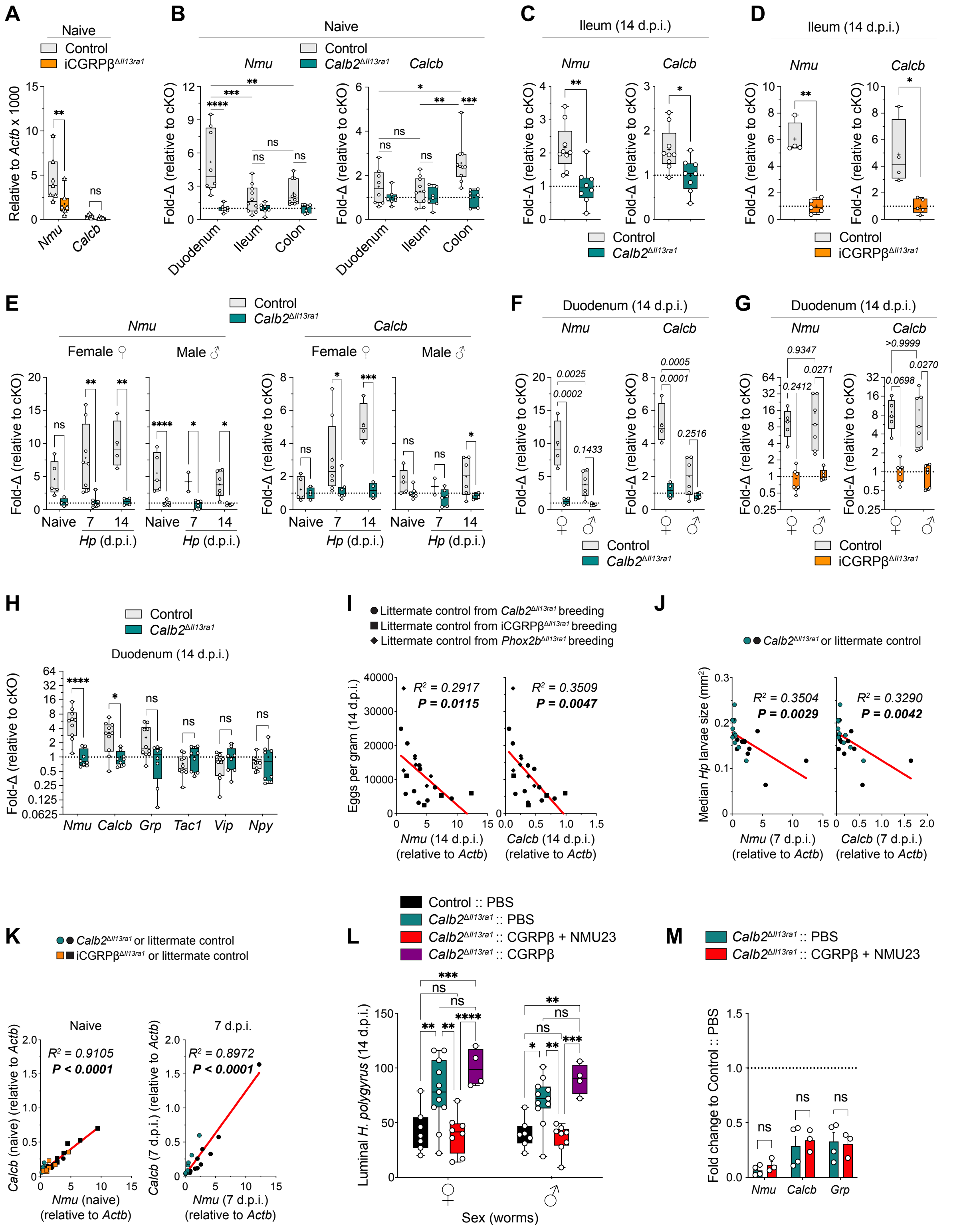

Supplemental fig. S8: PSN *Il13ra1* ablation and gut neuropeptide expression
(A) Duodenal qPCR gene expression of *Nmu* and *Calcb* in naive iCGRPβ$^{\Delta Il13ra1}$ cKO and littermate control mice after 3 or 5 consecutive doses of tamoxifen and euthanized 10 days after the first dose of tamoxifen. Data pooled from 2 independent experiments (*n* = 7 mice); △ represents BSA-administered control mice from Fig. 1K; gene expression is relative to *Actb* ($2^{-\Delta Ct}$) multiplied by 1,000. **(B)** qPCR gene expression of *Nmu* and *Calcb* in *Calb2*$^{\Delta Il13ra1}$ cKO and littermate control mice across different regions of the gut at steady-state. Data is pooled from 2 independent experiments (*n* = 7-8 mice). **(C** and **D)** Ileal qPCR gene expression of *Nmu* and *Calcb* at 14 d.p.i. in *H. polygyrus*-infected (C) *Calb2*$^{\Delta Il13ra1}$ cKO and littermate control mice (*n* = 8-9 mice) and (D) iCGRPβ$^{\Delta Il13ra1}$ cKO and littermate control mice after 3 or 5 consecutive doses of tamoxifen (*n* = 4-5 mice). Data represents 1 of ≥2 independent experiments. **(E** and **F)** Duodenal qPCR gene expression of *Nmu* and *Calcb* from data in Fig. 3A, divided by sex of mice. (E) Separately comparing female and male *Calb2*$^{\Delta Il13ra1}$ cKO and littermate control mice at steady-state, 7 d.p.i., and 14 d.p.i. (F) Comparing between female and male *Calb2*$^{\Delta Il13ra1}$ cKO and littermate control mice at 14 d.p.i. Data either pooled from 6 independent experiments (E) or represents 1 of ≥2 independent experiments (F). **(G)** Duodenal qPCR gene expression of *Nmu* and *Calcb* from data in Fig. 3B, comparing between female and male iCGRPβ$^{\Delta Il13ra1}$ cKO and littermate control mice after 3 or 5 consecutive doses of tamoxifen. Data pooled from 2 independent experiments. **(H)** Duodenal qPCR gene expression of various gut neuropeptides in *Calb2*$^{\Delta Il13ra1}$ cKO and littermate control mice at 14 d.p.i. Data represents 1 of ≥2 independent experiments (*n* = 8-10 mice). Gene expression is normalized to *Actb* ($2^{-\Delta Ct}$) and represented as fold-change relative to cKO group mean per experiment (B-H). **(I)** Scatter plots of littermate controls from Fig. 3D, correlating eggs per gram of feces at 14 d.p.i. (y-axes) with duodenal qPCR gene expression data normalized to *Actb* ($2^{-\Delta Ct}$ x 1,000) (x-axes) for *Nmu* (left) and *Calcb* (right) at 14 d.p.i. from experiments with matched data across different mouse models (represented by different shapes). Data pooled from 3 independent experiments (*n* = 21 mice). **(J)** Scatter plots correlating median Hp larvae size at 7 d.p.i. (y-axes) and duodenal qPCR gene expression data normalized to *Actb* ($2^{-\Delta Ct}$ x 1,000) (x-axes) for *Nmu* (left) and *Calcb* (right) at 7 d.p.i. Data pooled from 2 independent experiments (*n* = 10-12 mice). **(K)** Scatter plots correlating duodenal qPCR gene expression data normalized to *Actb* ($2^{-\Delta Ct}$ x 1,000) for *Nmu* (x-axes) and *Calcb* (y-axes) at steady-state (left) and 7 d.p.i. (right). **(L-M)** *H. polygyrus*-infected *Calb2*$^{\Delta Il13ra1}$ cKO mice were administered (i.p.) indicated neuropeptides as in Fig 3, F and G. (L) Quantification adult *H. polygyrus* in lumen at 14 d.p.i. by sex of worm (*n* = 4-11 mice). (M) Duodenal qPCR gene expression normalized to *Actb* ($2^{-\Delta Ct}$) and represented as fold-change relative to PBS-treated littermate control mouse per experiment. Data represents 1 of 2 independent experiments (*n* = 3-4 mice). Each bar graph (M) indicates the mean ± SEM of replicates. **P* < 0.05, ***P* < 0.01, ****P* < 0.001, *****P* < 0.0001 using 2way ANOVA with Sidak's (A, E, H, M) or Tukey's (B, F, G, L) correction, two-tailed Welch's t test (C, D), or simple linear regression (I, J, K).

Supplemental fig. S9

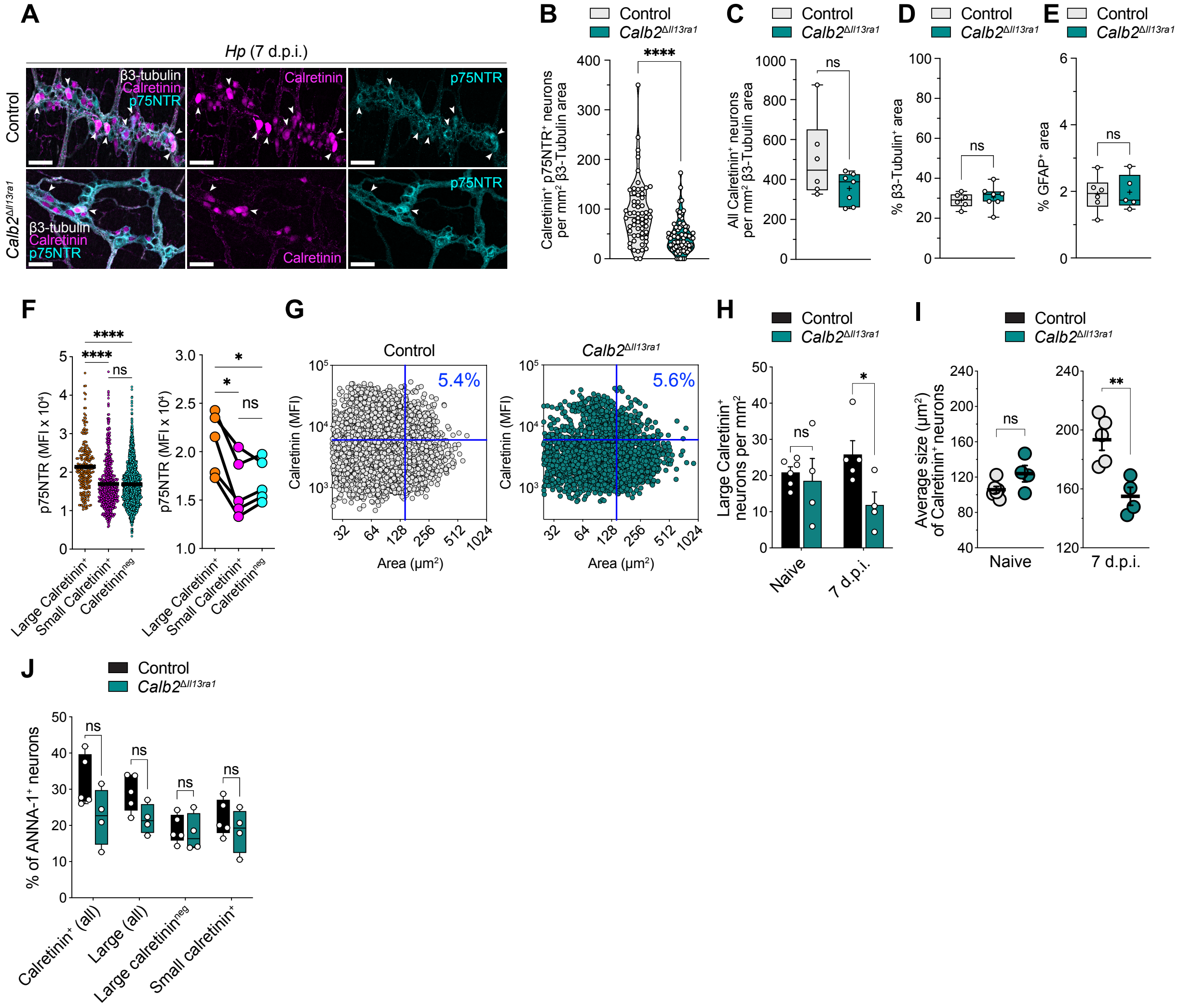

Supplemental fig. S9: PSN populations in *Calb2*$^{\Delta Il13ra1}$ cKO mice
(A-E) Wholemount confocal imaging of *Calb2*$^{\Delta Il13ra1}$ cKO and littermate control myenteric plexi at 7 d.p.i. (A) Representative image. β3-tubulin, calretinin, and p75NTR are pseudocolored white, magenta, and cyan, respectively. Arrows indicate PSNs (calretinin+p75NTR+ neurons); scale bars = 50µm. (B) Quantification of PSNs per mm^2 neuronal (β3-tubulin$^+$) area per field of view (each datapoint represents a field of view from 6-7 mice). (C) Quantification of all calretinin$^+$ neurons per mm^2 neuronal area. (D) Quantification of β3-tubulin$^+$ neuronal area as a percentage of total area. (E) Quantification of GFAP$^+$ glial cell area as a percentage of total area. Each datapoint represents a distinct mouse (C-E, n = 5-7 mice). **(F)** Quantification of mean fluorescent intensity (MFI) of p75NTR immunostaining in different enteric neuron subsets of control mice at 7 d.p.i. Large and small neurons are defined as neurons above or below, respectively, the 3rd quartile of neuron cell body area (HuCD$^+$ or ANNA-1$^+$) per experiment. (Left) p75NTR MFI of individually segmented neurons categorized by size and calretinin positivity (datapoints represent single neurons pooled across 5 mice). (Right) Average p75NTR MFI per mouse of indicated neuron subsets. Each datapoint represents an individual mouse (n = 5). **(G)** Scatter plot of individually segmented neurons pooled from naïve littermate controls (left, n = 6) and *Calb2*$^{\Delta Il13ra1}$ cKO (right, n = 4) mice. Each datapoint represents a single segmented neuron pooled from 4-6 mice. **(H)** Quantification of PSNs (large calretinin$^+$) per mm^2 of total area at steady-state and at 7 d.p.i. Data pooled from 2 independent experiments; each datapoint represents a single mouse (n = 4-6 mice). **(I)** Quantification of average size of segmented calretinin$^+$ neurons in (left) naïve and (right) 7 d.p.i. mice (n = 4-6 mice). **(J)** Quantification of different enteric neuron subsets as a percentage of all neurons in *Calb2*$^{\Delta Il13ra1}$ cKO and littermate control myenteric plexi at 7 d.p.i. Each datapoint represents a single mouse (n = 4-6 mice). Each bar and scatter plot (F, H) indicates the mean ± SEM of replicates. ns = not significant, *P < 0.05, **P < 0.01, ***P < 0.001, ****P < 0.0001 using 2way ANOVA with Sidak's correction (H, J), one-way ANOVA with Tukey's correction (F), or two-tailed Welch's t test (B-E, I).

Supplemental fig. S10

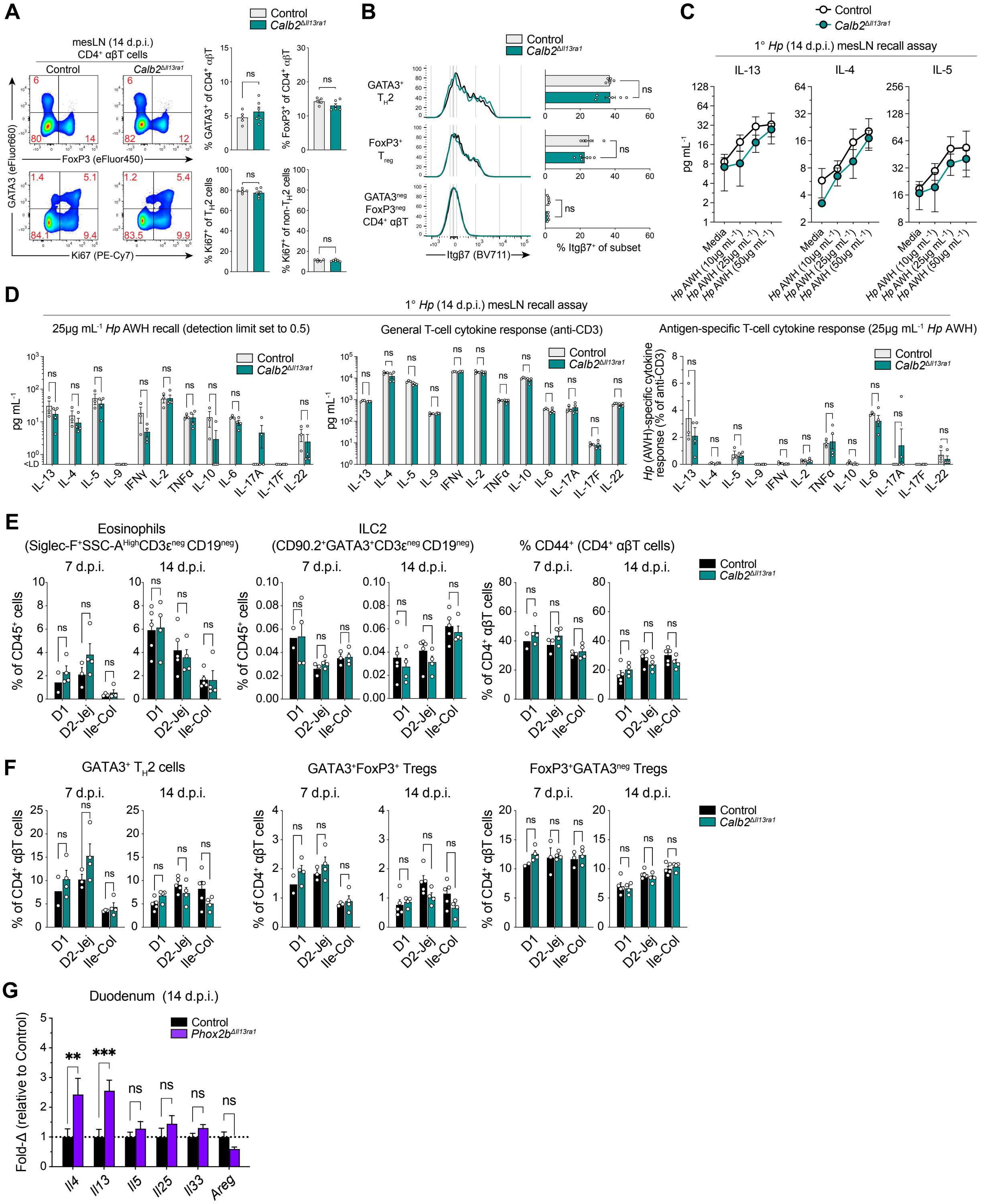

Supplemental fig. S10: *H. polygyrus*-specific adaptive immune and memory responses. **(A** and **B)** Flow cytometry of mesenteric lymph node (mesLN) αβT-cell populations from *H. polygyrus*-infected *Calb2*$^{\Delta Il13ra1}$ cKO and littermate control (*Il13ra1*^{flox}) mice at 14 d.p.i. (A) Representative flow plots gated on live CD45^{+}CD3ε^{+}TCRαβ^{+}CD4^{+}CD8a^{neg} cells (Left). Quantification of CD4+ αβT-cell subpopulations expressing indicated transcription factors (Right), TH2 denotes GATA3+ CD4+ αβT cells. (B) Histograms (left) and quantification (right) of Itgβ7 expression in indicated CD4+ αβT cell populations; each datapoint represents a distinct mouse (*n* = 5-6). **(C** and **D)** Antigen-recall assay using *H. polygyrus* adult worm homogenate (*Hp* AWH) on mesLN immune cells. (C) MesLN cells were plated and administered different concentrations of *Hp* AWH; supernatant IL-13, IL-4, and IL5 concentration was measured by LegendPlex. Datapoints represents mean ± SEM (*n* = 3-4 mice). (D) LegendPlex supernatant measurements from plated mesLN cells cultured with either *Hp* AWH (25 µg mL^{-1}) (Left) or plated anti-CD3 antibody (10 µg mL^{-1}) (Middle). *Hp* AWH response as a percentage of total plated anti-CD3 T-cell response (Right). Points below the limit of detection plotted as 0.5 pg mL^{-1}. Datapoints represent distinct mice (*n* = 3-4). (**E** and **F**) Flow cytometry of different gut-draining mesLN immune cell populations at 7 and 14 d.p.i. D1 = duodenal-draining (off of the main mesLN chain, near liver); D2-Jej = duodenal/jejunal-draining (on the main mesLN chain); Ile-Col = Ileum/colon/cecum-draining. Data includes 2 independent timepoints (*n* = 2-5 mice). **(G)** Duodenal qPCR gene expression in *H. polygyrus*-infected *Phox2b*$^{\Delta Il13ra1}$ cKO and littermate control mice at 14 d.p.i. Gene expression is normalized to *Actb* ($2^{-\Delta Ct}$) and represented as fold-change relative to Control group mean. Data represents 1 of ≥2 independent experiments (*n* = 7 mice). Each bar graph indicates the mean ± SEM of replicates. ns = not significant, **P* < 0.05, ***P* < 0.01, ****P* < 0.001, *****P* < 0.0001 using two-tailed Welch's t-test (A, B) or 2way ANOVA with Sidak's correction (D, E, F, G).

Supplemental fig. S11

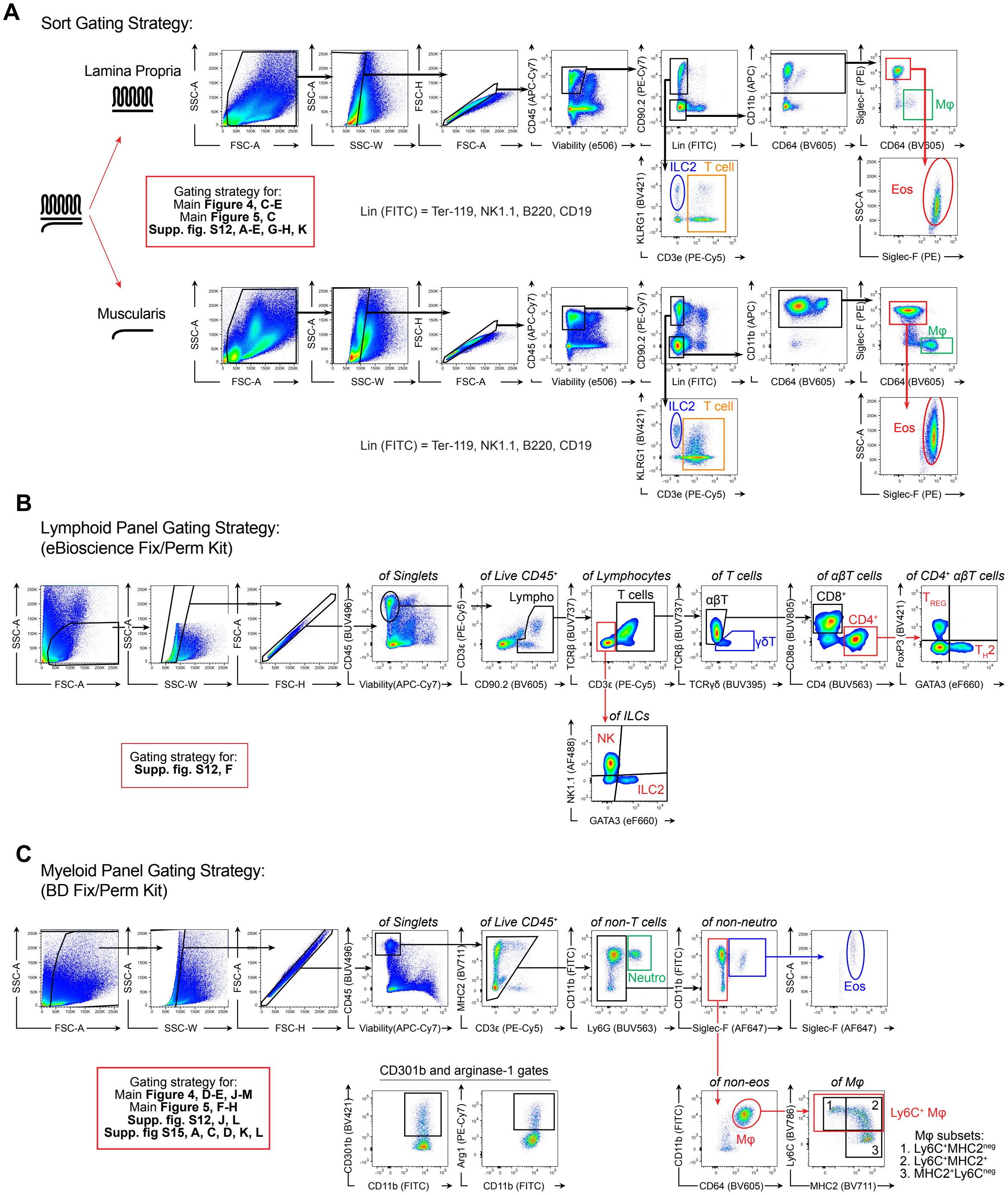

Supplemental fig. S11: Gating strategies for flow cytometry

(A) Representative flow cytometry gating strategy for indicated muscularis and lamina propria cell populations purified by FACS, including KLRG1+ ILC2, Eosinophils, macrophages (Mφ), and T cells. Example from ileal lamina propria (top) and muscularis (bottom) cells at 7 d.p.i. **(B)** Representative flow cytometry gating strategies for lymphoid cell populations, including GATA3+ ILC2, NK cells, CD4+ and CD8a+ αβT cells, GATA3+ T_H2 cells, FoxP3+ T_{REG}, and γδT cells. Example from duodenal muscularis cells at 5 d.p.i. **(C)** Representative flow cytometry gating strategies for myeloid cell populations, including neutrophils (Neutro), eosinophils (Eos), and different Mφ subsets. Example from duodenal muscularis cells at 3 d.p.i.

Supplemental fig. S12

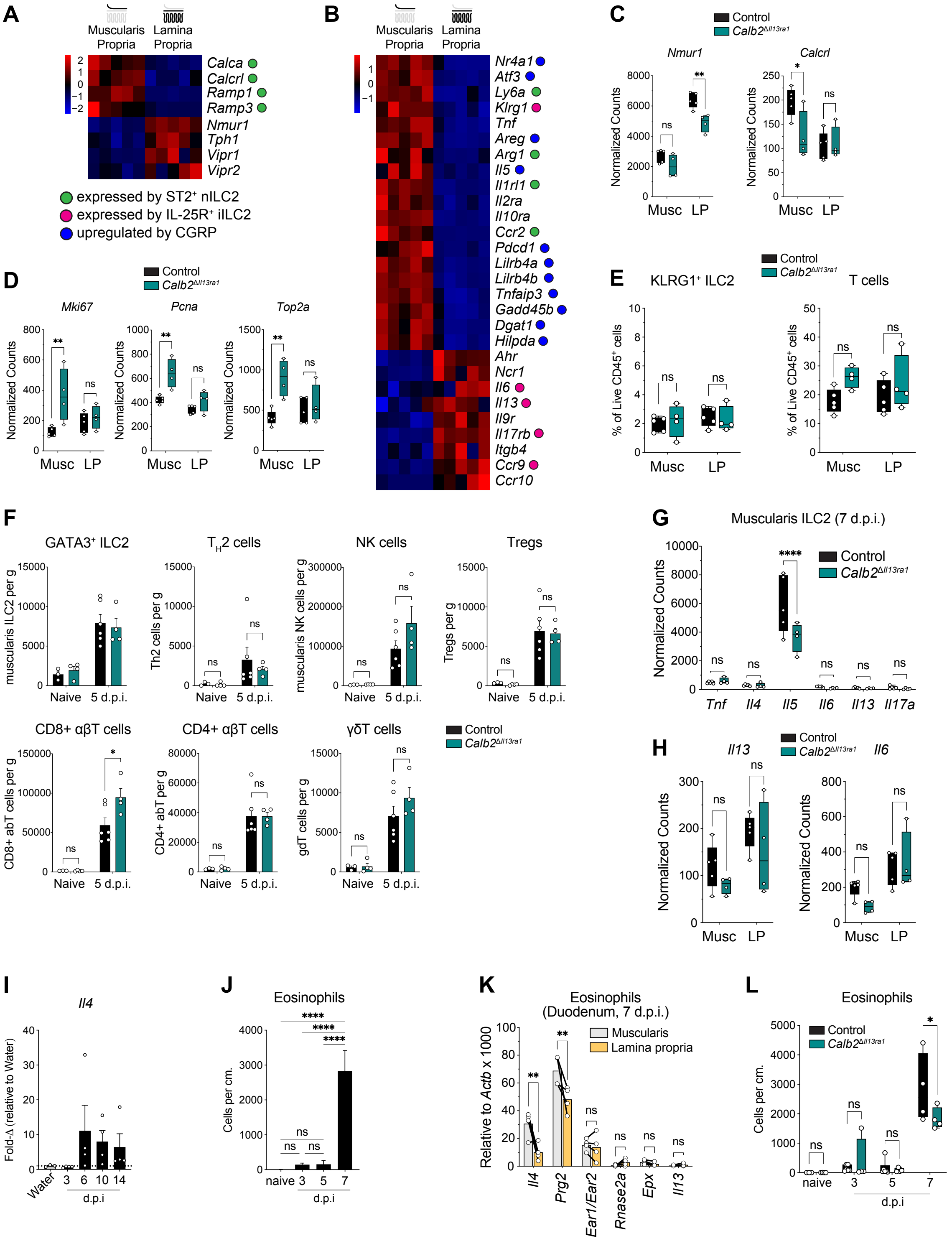

Supplemental fig. S12: Type 2 immune responses in the *Calb2*$^{\Delta Il13ra1}$ cKO muscularis and lamina propria

Bulk RNA-seq analysis of FACS-purified KLRG1^{+} ILC2s from the ileal muscularis and lamina propria of *Calb2*$^{\Delta Il13ra1}$ cKO and littermate control mice at 7 d.p.i.

(A and **B)** Heatmaps comparing DEGs between control ILC2s of the muscularis vs. lamina propria, highlighting select (A) neuropeptide-, neuropeptide receptor-, or neurotransmitter-related DEGs and (B) nILC2-, iILC2-, and CGRP signaling-related DEGs. Each heatmap column represents ILC2s from a single mouse (*n* = 5 mice). **(C** and **D)** DESeq2 normalized counts comparing indicated (C) neuropeptide receptor gene expression or (D) proliferation marker gene expression in muscularis (Musc) and lamina propria (LP) ILC2s from *Calb2*$^{\Delta Il13ra1}$ cKO and littermate control mice. Each datapoint represents a single mouse (*n* = 4-5 mice). **(E)** Flow cytometry of the ileal muscularis and LP. Quantification of KLRG1^{+} ILC2s (left) and CD3ε^{+} T cells (right) as a percentage of all CD45^{+} immune cells (*n* = 4-5 mice). **(F)** Flow cytometry of the duodenal muscularis at steady-state and at 5 d.p.i. Quantification of lymphoid cell subset abundance in the muscularis. Data is pooled from 2 independent experiments (*n* = 3-6 mice). **(G** and **H)** DESeq2 normalized counts from experiment described in A-D. (G) Expression of ILC2 cytokine genes in muscularis ILC2s. (H) Expression of *Il13* (left) and *Il6* (right) in muscularis and LP ILC2s. Each datapoint represents a single mouse (*n* = 4-5 mice). **(I)** Duodenal qPCR gene expression at steady-state (Water) and at various timepoints during *H. polygyrus* infection. . Gene expression is normalized to *Actb* ($2^{-\Delta Ct}$) and represented as fold-change relative to Water group mean. Data pooled from 5 independent experiments (n = 3-4 mice). **(J)** Flow cytometry of the duodenal muscularis in naïve and *H. polygyrus*-infected mice. Quantification of the eosinophil abundance in the duodenal muscularis. Data is pooled from 4 independent experiments (*n* = 3-6 mice). **(K)** qPCR gene expression of FACS-purified eosinophils (CD11b^{+} / CD64^{neg} / Siglec-F^{+} / SSC-A^{high}) from the duodenal muscularis and lamina propria at 7 d.p.i. (*n* = 4 mice). Gene expression is normalized to *Actb* ($2^{-\Delta Ct}$) and multiplied by 1,000. **(L)** Flow cytometry of the duodenal muscularis in naïve and *H. polygyrus*-infected *Calb2*$^{\Delta Il13ra1}$ cKO and littermate control mice. Quantification of the eosinophil abundance in the duodenal muscularis. Data is pooled from 4 independent experiments (*n* = 3-6 mice). Each bar graph indicates the mean ± SEM of replicates. ns = not significant, *$P < 0.05$, **$P < 0.01$, ***$P < 0.001$, ****$P < 0.0001$ using 2way ANOVA with Sidak's (C-H, K, L) or Tukey's (J) correction.

Supplemental fig. S13

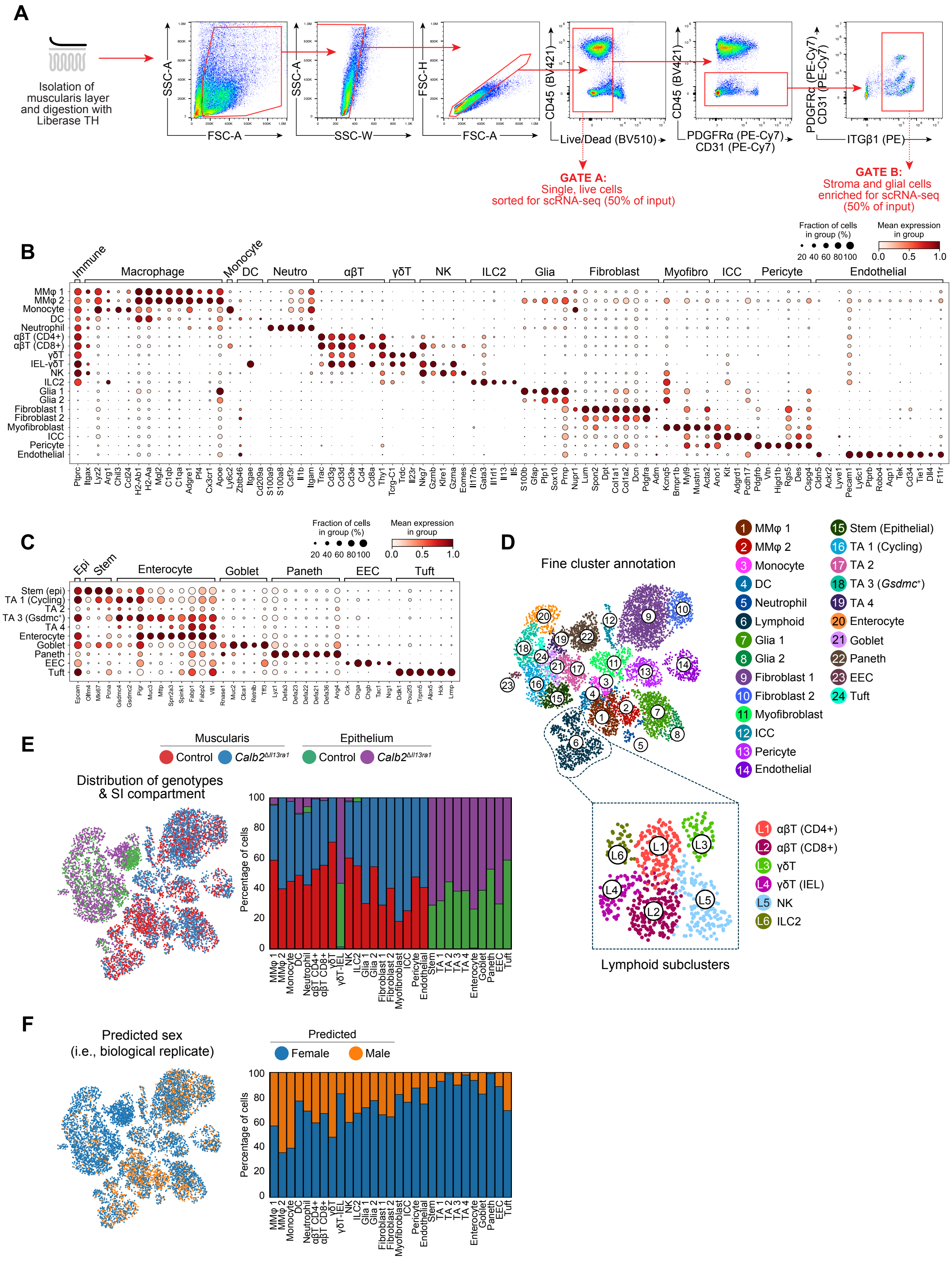

Supplemental fig. S13: scRNA-seq of the *H. polygyrus*-infected muscularis and epithelium
FACS-purification and scRNA-seq of ileal muscularis immune and non-immune cells and duodenal epithelial cells from *H. polygyrus*-infected *Calb2*$^{\Delta Il13ra1}$ cKO and littermate control (*Il13ra1*^{flox}) mice at 7 d.p.i. **(A)** Example FACS gating strategy for ileal muscularis cells. The scRNA-seq input for each sample consists of 50% live single-cells from Gate A and 50% CD45^{neg}Itgβ1^{+} cells from Gate B to enrich for glial cells and stromal cells. Each 10x Genomics encapsulation channel consists of the input from 1 female mouse and 1 male mouse of the same genotype pooled together in the same channel (*n* = 2 mice). **(B** and **C)** Dot plot of scRNA-seq data depicting marker genes used to distinguish fine-level annotation of (B) non-epithelial cell clusters and (C) epithelial cell clusters. The fine-level annotation was used to distinguish different clusters within the same cell type and highlight the differences in marker gene expression between these clusters. **(D)** UMAP depicting fine-level cluster annotation in different colors and numbers (1-24). Cluster 6 (Lymphoid) was subclustered to resolve distinct cell types (L1-L6), yielding 29 total fine-level clusters. **(E)** UMAP (left) and proportion plot (right) depicting the distribution of ileal muscularis and duodenal epithelial cells from *Calb2*$^{\Delta Il13ra1}$ cKO and littermate control mice. The MMφ 1 cluster contained a total of 24 cells from the duodenal epithelium samples and were removed from the general- and fine-level MMφ clusters for all differentially expressed gene (DEG) analyses, gene set enrichment analyses (GSEA), and violin plots. **(F)** UMAP (left) and proportion plot (right) depicting the distribution of the predicted sex of cells in each fine-level cluster and predicted using sex-specific gene modules.

Supplemental fig. S14

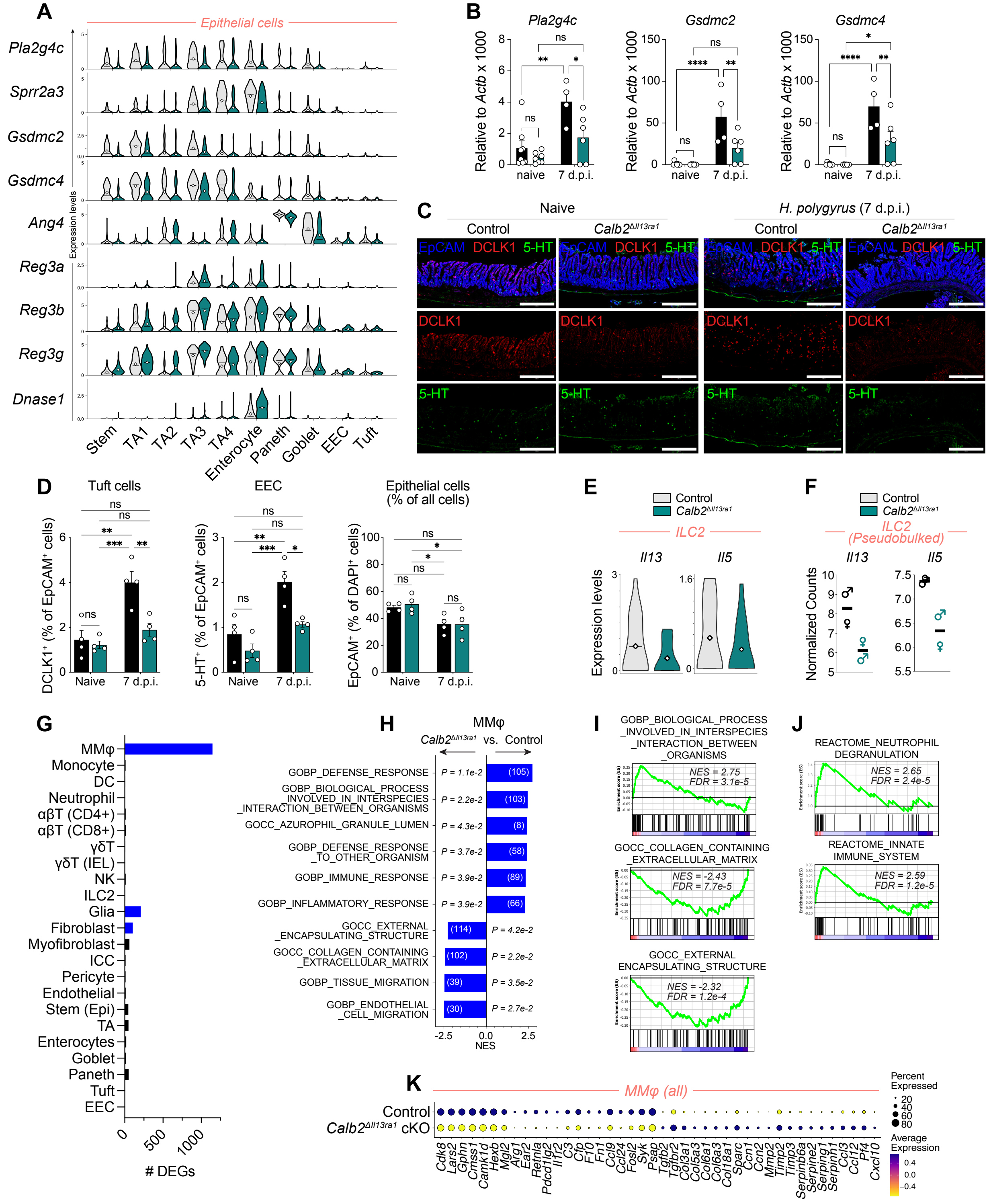

Supplemental fig. S14: Epithelial cell and muscularis immune cell defense responses to *H. polygyrus*

(A) Violin plots depicting the expression of genes associated with barrier defense and epithelial type 2 immune responses in fine-level scRNA-seq clusters from duodenal epithelial cells. **(B)** Duodenal qPCR gene expression of select epithelial scRNA-seq DEGs at steady-state and at 7 d.p.i. with *H. polygyrus*. Data is pooled from 3 independent experiments (*n* = 4-8 mice). **(C and D)** Axioscan immunofluorescence microscopy of cross-section duodena from naïve and *H. polygyrus*-infected (7 d.p.i.) *Calb2*$^{\Delta Il13ra1}$ cKO and littermate control (*Il13ra1*^{flox}) mice. (C) Representative images. Antibodies staining DCLK1, EpCAM, and 5-HT are depicted in red, blue, and green, respectively. (D) Quantification of DCLK1+ tuft cells and 5-HT+ enteroendocrine cells (EEC) as a percentage of total EpCAM+ epithelial cells and the fraction of epithelial cells imaged as a percentage of all DAPI+ cells imaged. **(E and F)** Type 2 cytokine gene expression in ileal muscularis ILC2 cluster. (E) Violin plots depicting scRNA-seq expression. (F) DESeq2 normalized counts of ILC2s categorized by predicted sex (i.e., biological replicate/mouse) and analyzed as pseudo-bulk RNA-seq samples. Gene expression of predicted females (♀) and males (♂) are indicated in plot (*n* = 2 mice). **(G)** Bar graph representing the number of significant differentially expressed genes (DEGs) between *Calb2*$^{\Delta Il13ra1}$ cKO and littermate control (*Il13ra1*^{flox}) samples in each general-level cluster. **(H)** Bar graph depicting the adjusted p values and normalized enrichment scores (NES) using fgsea analyses of curated Gene Ontology gene sets from MSigDB on the general-level MMφ cluster, comparing between *Calb2*$^{\Delta Il13ra1}$ cKO and littermate control cells. Number in parentheses indicates the number of genes in the corresponding gene set. **(I and J)** Gene Set Enrichment Analysis (GSEA) normalized enrichment score plots for select (I) Gene Ontology and (J) Reactome gene sets from MSigDB. NES and FDR q value (using 100,000 permutations) are indicated. **(K)** Dotplot on general-level MMφ cluster depicting various DEGs between *Calb2*$^{\Delta Il13ra1}$ cKO and littermate control cells. Each bar graph indicates the mean ± SEM of replicates. ns = not significant, $*P < 0.05$, $**P < 0.01$, $***P < 0.001$, $****P < 0.0001$ using 2way ANOVA with Tukey's correction (B, D).

Supplemental fig. S15

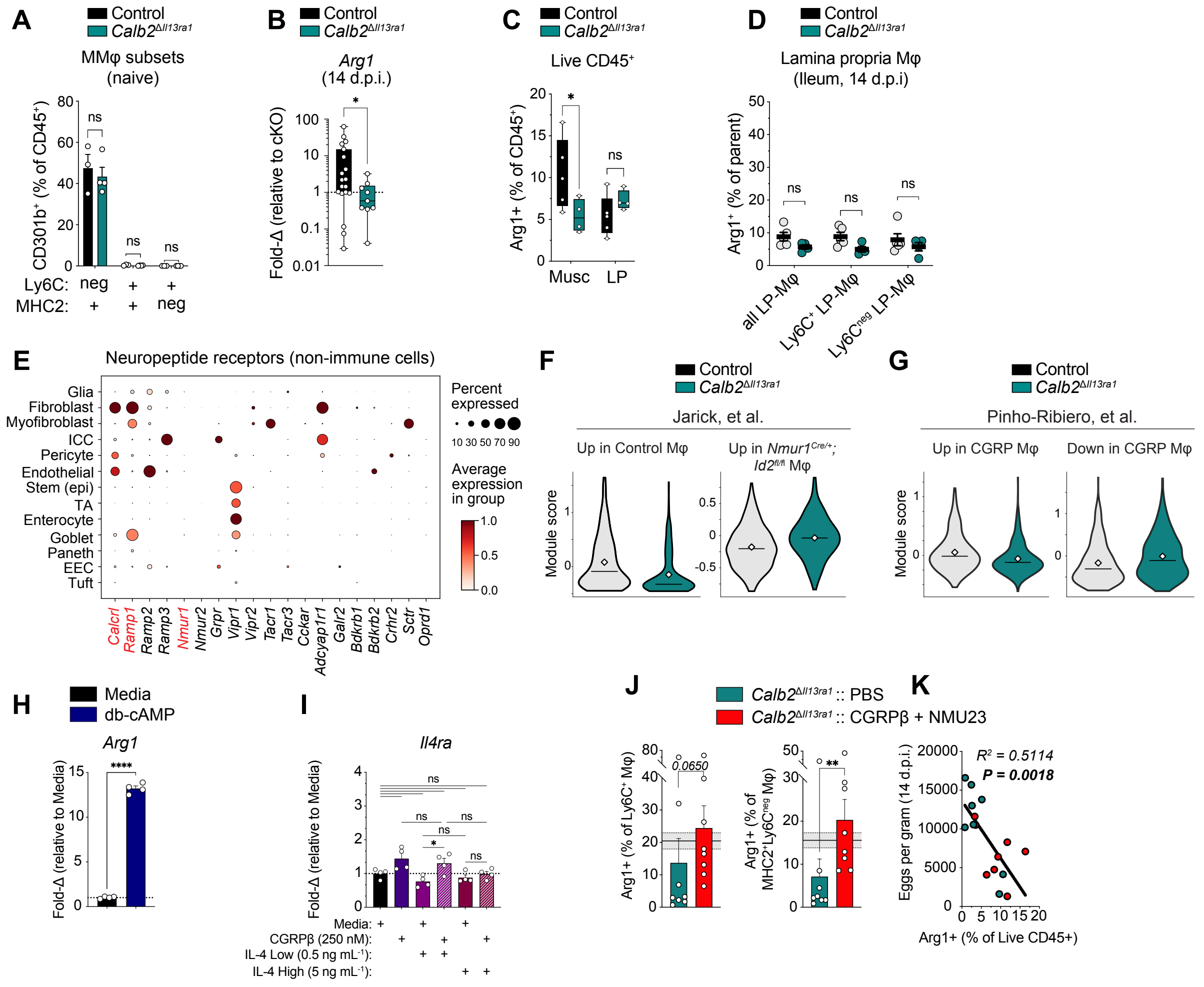

Supplemental fig. S15: CGRPβ promotes AAMφ marker expression in MMφ
(A) Flow cytometric analysis quantifying the frequency of CD301b$^+$ cells across different MMφ subsets (gated as in **fig. S11C**) in duodena of naïve *Calb2*$^{\Delta Il13ra1}$ cKO and littermate control mice. Data represents 1 of ≥2 independent experiments (*n* = 3-4 mice). **(B)** Duodenal qPCR gene expression in *H. polygyrus*-infected *Calb2*$^{\Delta Il13ra1}$ cKO and littermate control mice at 14 d.p.i. Gene expression is normalized to *Actb* ($2^{-\Delta Ct}$) and represented as fold-change relative to cKO group mean per experiment. Data is pooled from 2 independent experiments (*n* = 9-19 mice). **(C** and **D)** Flow cytometric analysis of ileal muscularis and lamina propria immune cells (as gated in **fig. S11C**) from *H. polygyrus*-infected *Calb2*$^{\Delta Il13ra1}$ cKO and littermate control mice at 14 d.p.i. (C) Frequencies of arginase-1$^+$ as a percentage of all live muscularis and lamina propria CD45$^+$ immune cells and (D) lamina propria Mφ subsets. Data represents 1 of ≥2 independent experiments (*n* = 4-5 mice). **(E)** Dot plot depicting select neuropeptide receptor gene expression in general non-immune cell clusters from scRNA-seq dataset presented in **Fig. 4F**. **(F and G)** Gene module scores of general MMφ cluster from scRNA-seq dataset presented in **Fig. 4F**. Gene modules curated from highlighted DEGs in (F) gut macrophages of control vs. ILC2-deficient mice from Jarick *et al.* (2022) and (G) BMDM/meningeal Mφ after treatment with *S. pneumoniae* ± CGRP of Pihno-Ribeiro *et al.* (2023). **(H** and **I)** qPCR gene expression of BMDM culture wells treated for 8 hours with (H) a membrane-permeable cyclic-AMP analogue (db-cAMP, 10µM) and (I) combinations of CGRPβ (250nM) and different concentrations IL-4. Expression data is normalized to *Actb* ($2^{-\Delta Ct}$) and represented as fold-change relative to media control group per experiment. Each datapoint represents an individual well of a culture pooled from 2-5 mice per experiment and differentiated into BMDMs (*n* = 4 wells). Data represents 1 of ≥2 independent experiments. **(J** and **K)** Flow cytometric analysis of ileal muscularis propria MMφ subsets from *H. polygyrus*-infected *Calb2*$^{\Delta Il13ra1}$ cKO mice treated with either PBS or a combination of CGRPβ and NMU23 peptides (2 nmoles, each, per injection) once a day and euthanized at 14 d.p.i. (J) Frequency of arginase-1$^+$ cells as a percentage of Ly6C$^+$ MMφ (left) and MHC2$^+$Ly6C^{neg} MMφ (right). Each datapoint represents an individual mouse (*n* = 8 mice). Data is pooled from 2 independent experiments. Solid and dotted lines with grayed area represent the mean and range of mean±SEM, respectively, of littermate control (*Il13ra1*$^{flox/flox}$) mice at 14 d.p.i. pooled from 4 independent experiments (*n* = 16 mice). (K) Scatter plot with linear regression correlating % arginase-1$^+$ cells of total immune cells (x-axis) with eggs per gram of feces (y-axis) at 14 d.p.i. from *Calb2*$^{\Delta Il13ra1}$ cKO mice treated daily with either PBS or a combination of CGRPβ and NMU23. Data is pooled from 2 independent experiments (*n* = 16 mice). Each bar and scatter plot indicates the mean ± SEM of replicates; plots with boxes and whiskers indicate median, quartiles, and range. ns = not significant, **P* < 0.05, ***P* < 0.01, ****P* < 0.001, *****P* < 0.0001 using 2way ANOVA with Sidak's correction (A, C, D), one-way ANOVA with Tukey's correction (I), two-tailed unpaired t-test (B, H), two-tailed Mann-Whitney test (J), and simple linear regression (K).

Supplemental fig. S16

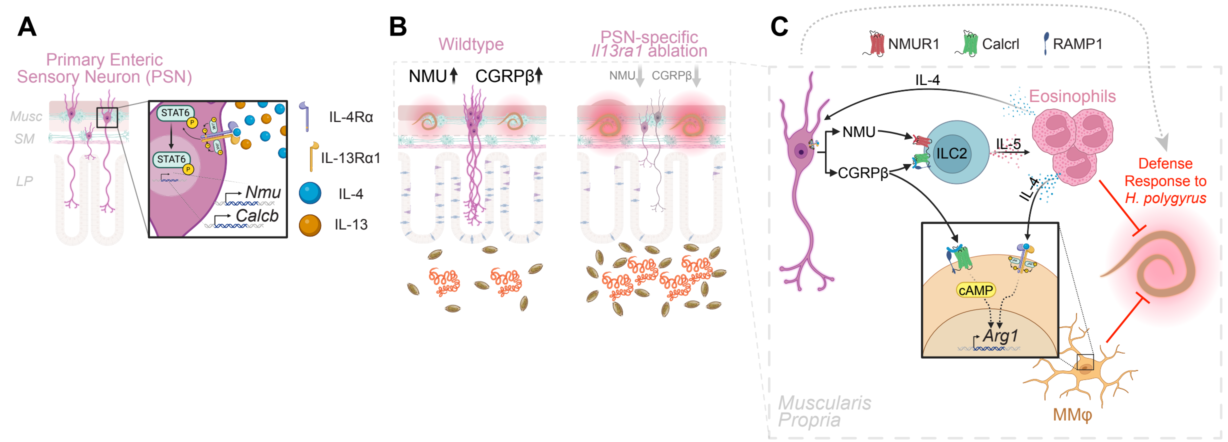

Supplemental fig. S16: Graphical summary

Schematics illustrating proposed models and mechanisms. (**A**) The type 2 cytokines IL-4 and IL-13 are directly sensed by enteric sensory neurons through the IL-4Rα/IL-13Rα1 heterodimeric receptor. Receptor engagement on enteric sensory neurons promotes the phosphorylation and nuclear-translocation of STAT6, thereby inducing *Nmu* and *Calcb* neuropeptide expression in a STAT6-dependent manner. **(B)** During the tissue-dwelling phase of *H. polygyrus* infection, larvae burrow through the submucosa and muscularis propria, developing near enteric neurons of the myenteric plexus and promoting muscularis-specific immune responses. Enteric neuron ablation of IL-13Rα1 reduces small intestinal gene expression of NMU and CGRPβ and diminishes the abundance and neurite density of enteric sensory neurons in the myenteric plexus and villi, respectively. This reduction of enteric sensory neurons and neuropeptides impairs host control over helminth fitness and infectious burden and during the luminal phase of *H. polygyrus*. **(C)** Mechanistically, NMU23 and CGRPβ released by enteric sensory neurons act on muscularis ILC2s, which respond by upregulating IL-5, increasing the recruitment of IL-4-producing eosinophils to the muscularis during the late tissue-dwelling phase of *H. polygyrus* infection. Additionally, increased muscularis IL-4 drives alternatively-activated MMφ differentiation which is essential for *H. polygyrus* larval entrapment. CGRPβ activates the co-receptors Calcrl and RAMP1 expressed by MMφ, promoting intracellular cAMP signaling and synergizing with IL-4 to drive the expression of the anti-helminthic mediator arginase-1. Black lines indicate induction; red lines indicate inhibition. Dotted gray arrow from the enteric sensory neuron to defense response indicates the action of NMU23, CGRPβ, or other enteric sensory neuron-derived mediators on non-immune cells in the muscularis and epithelium, such as fibroblasts and goblet cells. Schematic created with Biorender.com.

Movie S1: Proximity of tissue-dwelling H. polygyrus larvae to enteric neurons.
Associated media for **Fig. 2B**. Confocal z-stack imaging and 3-dimensional rendering (Aivia) of tissue-cleared duodenal muscularis propria at 6 d.p.i. Autofluorescent *H. polygyrus* larvae are pseudocolored green, white, and pink; enteric neurons (β3-tubulin) and CGRP are pseudocolored red and cyan, respectively.

References and notes

1. C. S. N. Klose, T. Mahlakõiv, J. B. Moeller, L. C. Rankin, A.-L. Flamar, H. Kabata, L. A. Monticelli, S. Moriyama, G. G. Putzel, N. Rakhilin, X. Shen, E. Kostenis, G. M. König, T. Senda, D. Carpenter, D. L. Farber, D. Artis, The neuropeptide neuromedin U stimulates innate lymphoid cells and type 2 inflammation. *Nature* **549**, 282–286 (2017). doi:10.1038/nature23676 Medline

2. V. Cardoso, J. Chesné, H. Ribeiro, B. García-Cassani, T. Carvalho, T. Bouchery, K. Shah, N. L. Barbosa-Morais, N. Harris, H. Veiga-Fernandes, Neuronal regulation of type 2 innate lymphoid cells via neuromedin U. *Nature* **549**, 277–281 (2017). doi:10.1038/nature23469 Medline

3. A. Wallrapp, S. J. Riesenfeld, P. R. Burkett, R.-E. E. Abdulnour, J. Nyman, D. Dionne, M. Hofree, M. S. Cuoco, C. Rodman, D. Farouq, B. J. Haas, T. L. Tickle, J. J. Trombetta, P. Baral, C. S. N. Klose, T. Mahlakõiv, D. Artis, O. Rozenblatt-Rosen, I. M. Chiu, B. D. Levy, M. S. Kowalczyk, A. Regev, V. K. Kuchroo, The neuropeptide NMU amplifies ILC2-driven allergic lung inflammation. *Nature* **549**, 351–356 (2017). doi:10.1038/nature24029 Medline

4. J. Talbot, P. Hahn, L. Kroehling, H. Nguyen, D. Li, D. R. Littman, Feeding-dependent VIP neuron-ILC3 circuit regulates the intestinal barrier. *Nature* **579**, 575–580 (2020). doi:10.1038/s41586-020-2039-9 Medline

5. A. Wallrapp, P. R. Burkett, S. J. Riesenfeld, S.-J. Kim, E. Christian, R.-E. E. Abdulnour, P. I. Thakore, A. Schnell, C. Lambden, R. H. Herbst, P. Khan, K. Tsujikawa, R. J. Xavier, I. M. Chiu, B. D. Levy, A. Regev, V. K. Kuchroo, Calcitonin Gene-Related Peptide Negatively Regulates Alarmin-Driven Type 2 Innate Lymphoid Cell Responses. *Immunity* **51**, 709–723.e6 (2019). doi:10.1016/j.immuni.2019.09.005 Medline

6. P. Hanč, R. J. Gonzalez, I. B. Mazo, Y. Wang, T. Lambert, G. Ortiz, E. W. Miller, U. H. von Andrian, Multimodal control of dendritic cell functions by nociceptors. *Science* **379**, eabm5658 (2023). Medline

7. M. Balood, M. Ahmadi, T. Eichwald, A. Ahmadi, A. Majdoubi, K. Roversi, K. Roversi, C. T. Lucido, A. C. Restaino, S. Huang, L. Ji, K.-C. Huang, E. Semerena, S. C. Thomas, A. E. Trevino, H. Merrison, A. Parrin, B. Doyle, D. W. Vermeer, W. C. Spanos, C. S. Williamson, C. R. Seehus, S. L. Foster, H. Dai, C. J. Shu, M. Rangachari, J. Thibodeau, S. V Del Rincon, R. Drapkin, M. Rafei, N. Ghasemlou, P. D. Vermeer, C. J. Woolf, S. Talbot, Nociceptor neurons affect cancer immunosurveillance. *Nature* **611**, 405–412 (2022). doi:10.1038/s41586-022-05374-w Medline

8. F. A. Pinho-Ribeiro, L. Deng, D. V. Neel, O. Erdogan, H. Basu, D. Yang, S. Choi, A. J. Walker, S. Carneiro-Nascimento, K. He, G. Wu, B. Stevens, K. S. Doran, D. Levy, I. M. Chiu, Bacteria hijack a meningeal neuroimmune axis to facilitate brain invasion. *Nature* **615**, 472–481 (2023). doi:10.1038/s41586-023-05753-x Medline

9. R. M. Maizels, J. P. Hewitson, J. Murray, Y. M. Harcus, B. Dayer, K. J. Filbey, J. R. Grainger, H. J. McSorley, L. A. Reynolds, K. A. Smith, Immune modulation and modulators in Heligmosomoides polygyrus infection. *Exp. Parasitol.* **132**, 76–89 (2012). doi:10.1016/j.exppara.2011.08.011 Medline

10. E. Drokhlyansky, C. S. Smillie, N. Van Wittenberghe, M. Ericsson, G. K. Griffin, G. Eraslan, D. Dionne, M. S. Cuoco, M. N. Goder-Reiser, T. Sharova, O. Kuksenko, A. J. Aguirre, G. M. Boland, D. Graham, O. Rozenblatt-Rosen, R. J. Xavier, A. Regev, The Human and Mouse Enteric Nervous System at Single-Cell Resolution. *Cell* **182**, 1606–1622.e23 (2020). doi:10.1016/j.cell.2020.08.003 Medline

11. K. Morarach, A. Mikhailova, V. Knoflach, F. Memic, R. Kumar, W. Li, P. Ernfors, U. Marklund, Diversification of molecularly defined myenteric neuron classes revealed by single-cell RNA sequencing. *Nat. Neurosci.* **24**, 34–46 (2021). doi:10.1038/s41593-020-00736-x Medline

12. A. Zeisel, H. Hochgerner, P. Lönnerberg, A. Johnsson, F. Memic, J. van der Zwan, M. Häring, E. Braun, L. E. Borm, G. La Manno, S. Codeluppi, A. Furlan, K. Lee, N. Skene, K. D. Harris, J. Hjerling-Leffler, E. Arenas, P. Ernfors, U. Marklund, S. Linnarsson, Molecular Architecture of the Mouse Nervous System. *Cell* **174**, 999–1014.e22 (2018). doi:10.1016/j.cell.2018.06.021 Medline

13. T. J. Hibberd, W. P. Yew, K. N. Dodds, Z. Xie, L. Travis, S. J. Brookes, M. Costa, H. Hu, N. J. Spencer, Quantification of CGRP-immunoreactive myenteric neurons in mouse colon. *J. Comp. Neurol.* **530**, 3209–3225 (2022). doi:10.1002/cne.25403 Medline

14. M. M. Scott, K. W. Williams, J. Rossi, C. E. Lee, J. K. Elmquist, Leptin receptor expression in hindbrain Glp-1 neurons regulates food intake and energy balance in mice. *J. Clin. Invest.* **121**, 2413–2421 (2011). doi:10.1172/JCI43703 Medline

15. J. C. Nussbaum, S. J. Van Dyken, J. von Moltke, L. E. Cheng, A. Mohapatra, A. B. Molofsky, E. E. Thornton, M. F. Krummel, A. Chawla, H.-E. Liang, R. M. Locksley, Type 2 innate lymphoid cells control eosinophil homeostasis. *Nature* **502**, 245–248 (2013). doi:10.1038/nature12526 Medline

16. H. Xu, J. Ding, C. B. M. Porter, A. Wallrapp, M. Tabaka, S. Ma, S. Fu, X. Guo, S. J. Riesenfeld, C. Su, D. Dionne, L. T. Nguyen, A. Lefkovith, O. Ashenberg, P. R. Burkett, H. N. Shi, O. Rozenblatt-Rosen, D. B. Graham, V. K. Kuchroo, A. Regev, R. J. Xavier, Transcriptional Atlas of Intestinal Immune Cells Reveals that Neuropeptide α-CGRP Modulates Group 2 Innate Lymphoid Cell Responses. *Immunity* **51**, 696–708.e9 (2019). doi:10.1016/j.immuni.2019.09.004 Medline

17. H. Nagashima, T. Mahlakõiv, H.-Y. Shih, F. P. Davis, F. Meylan, Y. Huang, O. J. Harrison, C. Yao, Y. Mikami, J. F. Urban Jr., K. M. Caron, Y. Belkaid, Y. Kanno, D. Artis, J. J. O'Shea, Neuropeptide CGRP Limits Group 2 Innate Lymphoid Cell Responses and Constrains Type 2 Inflammation. *Immunity* **51**, 682–695.e6 (2019). doi:10.1016/j.immuni.2019.06.009 Medline

18. Y. Huang, K. Mao, X. Chen, M.-A. Sun, T. Kawabe, W. Li, N. Usher, J. Zhu, J. F. Urban Jr., W. E. Paul, R. N. Germain, S1P-dependent interorgan trafficking of group 2 innate lymphoid cells supports host defense. *Science* **359**, 114–119 (2018). doi:10.1126/science.aam5809 Medline

19. T. Ahrends, B. Aydin, F. Matheis, C. H. Classon, F. Marchildon, G. C. Furtado, S. A. Lira, D. Mucida, Enteric pathogens induce tissue tolerance and prevent neuronal loss from subsequent infections. *Cell* **184**, 5715–5727.e12 (2021). doi:10.1016/j.cell.2021.10.004 Medline

20. J. P. Hewitson, K. J. Filbey, J. Esser-von Bieren, M. Camberis, C. Schwartz, J. Murray, L. A. Reynolds, N. Blair, E. Robertson, Y. Harcus, L. Boon, S. C.-C. Huang, L. Yang, Y. Tu, M. J. Miller, D. Voehringer, G. Le Gros, N. Harris, R. M. Maizels, Concerted activity of IgG1 antibodies and IL-4/IL-25-dependent effector cells trap helminth larvae in the tissues following vaccination with defined secreted antigens, providing sterile immunity to challenge infection. *PLOS Pathog.* **11**, e1004676 (2015). doi:10.1371/journal.ppat.1004676 Medline

21. J. Esser-von Bieren, I. Mosconi, R. Guiet, A. Piersgilli, B. Volpe, F. Chen, W. C. Gause, A. Seitz, J. S. Verbeek, N. L. Harris, Antibodies trap tissue migrating helminth larvae and prevent tissue damage by driving IL-4Rα-independent alternative differentiation of macrophages. *PLOS Pathog.* **9**, e1003771 (2013). doi:10.1371/journal.ppat.1003771 Medline

22. F. Chen, D. W. El-Naccache, J. J. Ponessa, A. Lemenze, V. Espinosa, W. Wu, K. Lothstein, L. Jin, O. Antao, J. S. Weinstein, P. Damani-Yokota, K. Khanna, P. J. Murray, A. Rivera, M. C. Siracusa, W. C. Gause, Helminth resistance is mediated by differential activation of recruited monocyte-derived alveolar macrophages and arginine depletion. *Cell Rep.* **38**, 110215 (2022). doi:10.1016/j.celrep.2021.110215 Medline

23. J. Esser-von Bieren, B. Volpe, M. Kulagin, D. B. Sutherland, R. Guiet, A. Seitz, B. J. Marsland, J. S. Verbeek, N. L. Harris, Antibody-mediated trapping of helminth larvae requires CD11b and Fcγ receptor I. *J. Immunol.* **194**, 1154–1163 (2015). doi:10.4049/jimmunol.1401645 Medline

24. G. Coakley, N. L. Harris, Interactions between macrophages and helminths. *Parasite Immunol.* **42**, e12717 (2020). doi:10.1111/pim.12717 Medline

25. K. J. Jarick, P. M. Topczewska, M. O. Jakob, H. Yano, M. Arifuzzaman, X. Gao, S. Boulekou, V. Stokic-Trtica, P. S. Leclère, A. Preußer, Z. A. Rompe, A. Stamm, A. M. Tsou, C. Chu, F. R. Heinrich, G. M. Guerra, P. Durek, A. Ivanov, D. Beule, S. Helfrich, C. U. Duerr, A. A. Kühl, C. Stehle, C. Romagnani, M.-F. Mashreghi, A. Diefenbach, D. Artis, C. S. N. Klose, Non-redundant functions of group 2 innate lymphoid cells. *Nature* **611**, 794–800 (2022). doi:10.1038/s41586-022-05395-5 Medline

26. R. M. Anthony, J. F. Urban Jr., F. Alem, H. A. Hamed, C. T. Rozo, J.-L. Boucher, N. Van Rooijen, W. C. Gause, T. Memory, Memory T(H)2 cells induce alternatively activated macrophages to mediate protection against nematode parasites. *Nat. Med.* **12**, 955–960 (2006). doi:10.1038/nm1451 Medline

27. N. G. Copeland, N. A. Jenkins, D. L. Court, Recombineering: A powerful new tool for mouse functional genomics. *Nat. Rev. Genet.* **2**, 769–779 (2001). doi:10.1038/35093556 Medline

28. S. Warming, N. Costantino, D. L. Court, N. A. Jenkins, N. G. Copeland, Simple and highly efficient BAC recombineering using galK selection. *Nucleic Acids Res.* **33**, e36 (2005). doi:10.1093/nar/gni035 Medline

29. C. Perner, C. L. Sokol, Protocol for dissection and culture of murine dorsal root ganglia neurons to study neuropeptide release. *STAR Protoc.* **2**, 100333 (2021). doi:10.1016/j.xpro.2021.100333 Medline

30. G. Toda, T. Yamauchi, T. Kadowaki, K. Ueki, Preparation and culture of bone marrow-derived macrophages from mice for functional analysis. *STAR Protoc.* **2**, 100246 (2020). doi:10.1016/j.xpro.2020.100246 Medline

31. C. J. C. Johnston, E. Robertson, Y. Harcus, J. R. Grainger, G. Coakley, D. J. Smyth, H. J. McSorley, R. Maizels, Cultivation of Heligmosomoides polygyrus: An immunomodulatory nematode parasite and its secreted products. *J. Vis. Exp.* **98**, e52412 (2015). Medline

32. F. D. Finkelman, K. B. Madden, S. C. Morris, J. M. Holmes, N. Boiani, I. M. Katona, C. R. Maliszewski, Anti-cytokine antibodies as carrier proteins. Prolongation of in vivo effects of exogenous cytokines by injection of cytokine-anti-cytokine antibody complexes. *J. Immunol.* **151**, 1235–1244 (1993). doi:10.4049/jimmunol.151.3.1235 Medline

33. X. Zhu, L. Huang, Y. Zheng, Y. Song, Q. Xu, J. Wang, K. Si, S. Duan, W. Gong, Ultrafast optical clearing method for three-dimensional imaging with cellular resolution. *Proc. Natl. Acad. Sci. U.S.A.* **116**, 11480–11489 (2019). doi:10.1073/pnas.1819583116 Medline

34. W. Li, R. N. Germain, M. Y. Gerner, High-dimensional cell-level analysis of tissues with Ce3D multiplex volume imaging. *Nat. Protoc.* **14**, 1708–1733 (2019). doi:10.1038/s41596-019-0156-4 Medline

35. M. Häring, M. Fatt, J. Kupari, Protocol to Prepare Single-Cell Suspensions from Mouse Vagal Sensory Ganglia for Transcriptomic Studies. *STAR Protoc.* **1**, 100030 (2020). doi:10.1016/j.xpro.2020.100030 Medline

36. D. Esterházy, M. C. C. Canesso, L. Mesin, P. A. Muller, T. B. R. de Castro, A. Lockhart, M. ElJalby, A. M. C. Faria, D. Mucida, Compartmentalized gut lymph node drainage dictates adaptive immune responses. *Nature* **569**, 126–130 (2019). doi:10.1038/s41586-019-1125-3 Medline

37. C.-C. Chen, S. Louie, B. McCormick, W. A. Walker, H. N. Shi, Concurrent infection with an intestinal helminth parasite impairs host resistance to enteric Citrobacter rodentium and enhances Citrobacter-induced colitis in mice. *Infect. Immun.* **73**, 5468–5481 (2005). doi:10.1128/IAI.73.9.5468-5481.2005 Medline

38. S. Picelli, O. R. Faridani, Å. K. Björklund, G. Winberg, S. Sagasser, R. Sandberg, Full-length RNA-seq from single cells using Smart-seq2. *Nat. Protoc.* **9**, 171–181 (2014). doi:10.1038/nprot.2014.006 Medline

39. B. Li, J. Gould, Y. Yang, S. Sarkizova, M. Tabaka, O. Ashenberg, Y. Rosen, M. Slyper, M. S. Kowalczyk, A.-C. Villani, T. Tickle, N. Hacohen, O. Rozenblatt-Rosen, A. Regev, Cumulus provides cloud-based data analysis for large-scale single-cell and single-nucleus RNA-seq. *Nat. Methods* **17**, 793–798 (2020). doi:10.1038/s41592-020-0905-x Medline

40. A. Dobin, C. A. Davis, F. Schlesinger, J. Drenkow, C. Zaleski, S. Jha, P. Batut, M. Chaisson, T. R. Gingeras, STAR: Ultrafast universal RNA-seq aligner. *Bioinformatics* **29**, 15–21 (2013). doi:10.1093/bioinformatics/bts635 Medline

41. B. Li, C. N. Dewey, RSEM: Accurate transcript quantification from RNA-Seq data with or without a reference genome. *BMC Bioinformatics* **12**, 323 (2011). doi:10.1186/1471-2105-12-323 Medline

42. M. I. Love, W. Huber, S. Anders, Moderated estimation of fold change and dispersion for RNA-seq data with DESeq2. *Genome Biol.* **15**, 550 (2014). doi:10.1186/s13059-014-0550-8 Medline

43. F. A. Wolf, P. Angerer, F. J. Theis, SCANPY: Large-scale single-cell gene expression data analysis. *Genome Biol.* **19**, 15 (2018). doi:10.1186/s13059-017-1382-0 Medline

44. S. L. Wolock, R. Lopez, A. M. Klein, Scrublet: Computational Identification of Cell Doublets in Single-Cell Transcriptomic Data. *Cell Syst.* **8**, 281–291.e9 (2019). doi:10.1016/j.cels.2018.11.005 Medline

45. P.-L. Germain, A. Lun, C. Garcia Meixide, W. Macnair, M. D. Robinson, Doublet identification in single-cell sequencing data using *scDblFinder*. *F1000 Res.* **10**, 979 (2021). doi:10.12688/f1000research.73600.2 Medline

46. L. McInnes, J. Healy, J. Melville, UMAP: Uniform Manifold Approximation and Projection for Dimension Reduction. arXiv:1802.03426 [stat.ML] (2018).

47. V. A. Traag, L. Waltman, N. J. van Eck, From Louvain to Leiden: Guaranteeing well-connected communities. *Sci. Rep.* **9**, 5233 (2019). doi:10.1038/s41598-019-41695-z Medline

48. C. Domínguez Conde, C. Xu, L. B. Jarvis, D. B. Rainbow, S. B. Wells, T. Gomes, S. K. Howlett, O. Suchanek, K. Polanski, H. W. King, L. Mamanova, N. Huang, P. A. Szabo, L. Richardson, L. Bolt, E. S. Fasouli, K. T. Mahbubani, M. Prete, L. Tuck, N. Richoz, Z. K. Tuong, L. Campos, H. S. Mousa, E. J. Needham, S. Pritchard, T. Li, R. Elmentaite, J. Park, E. Rahmani, D. Chen, D. K. Menon, O. A. Bayraktar, L. K. James, K. B. Meyer, N. Yosef, M. R. Clatworthy, P. A. Sims, D. L. Farber, K. Saeb-Parsy, J. L. Jones, S. A. Teichmann, Cross-tissue immune cell analysis reveals tissue-specific features in humans. *Science* **376**, eabl5197 (2022). doi:10.1126/science.abl5197 Medline

49. C. Xu, M. Prete, S. Webb, L. Jardine, B. J. Stewart, R. Hoo, P. He, K. B. Meyer, S. A. Teichmann, Automatic cell-type harmonization and integration across Human Cell Atlas datasets. *Cell* **186**, 5876–5891.e20 (2023). doi:10.1016/j.cell.2023.11.026 Medline

50. I. Tirosh, B. Izar, S. M. Prakadan, M. H. Wadsworth 2nd, D. Treacy, J. J. Trombetta, A. Rotem, C. Rodman, C. Lian, G. Murphy, M. Fallahi-Sichani, K. Dutton-Regester, J.-R. Lin, O. Cohen, P. Shah, D. Lu, A. S. Genshaft, T. K. Hughes, C. G. K. Ziegler, S. W. Kazer, A. Gaillard, K. E. Kolb, A.-C. Villani, C. M. Johannessen, A. Y. Andreev, E. M. Van Allen, M. Bertagnolli, P. K. Sorger, R. J. Sullivan, K. T. Flaherty, D. T. Frederick, J. Jané-Valbuena, C. H. Yoon, O. Rozenblatt-Rosen, A. K. Shalek, A. Regev, L. A. Garraway, Dissecting the multicellular ecosystem of metastatic melanoma by single-cell RNA-seq. *Science* **352**, 189–196 (2016). doi:10.1126/science.aad0501 Medline

51. Y. Hao, T. Stuart, M. H. Kowalski, S. Choudhary, P. Hoffman, A. Hartman, A. Srivastava, G. Molla, S. Madad, C. Fernandez-Granda, R. Satija, Dictionary learning for integrative, multimodal and scalable single-cell analysis. *Nat. Biotechnol.* **42**, 293–304 (2024). doi:10.1038/s41587-023-01767-y Medline

52. G. Korotkevich, V. Sukhov, N. Budin, B. Shpak, M. N. Artyomov, A. Sergushichev, Fast gene set enrichment analysis, bioRxiv 060012 [Preprint] (2016); https://doi.org/10.1101/060012.

53. C. Hafemeister, R. Satija, Normalization and variance stabilization of single-cell RNA-seq data using regularized negative binomial regression. *Genome Biol.* **20**, 296 (2019). doi:10.1186/s13059-019-1874-1 Medline

54. C. Ahlmann-Eltze, W. Huber, glmGamPoi: Fitting Gamma-Poisson generalized linear models on single cell count data. *Bioinformatics* **36**, 5701–5702 (2021). doi:10.1093/bioinformatics/btaa1009 Medline

55. T. Stuart, A. Butler, P. Hoffman, C. Hafemeister, E. Papalexi, W. M. Mauck 3rd, Y. Hao, M. Stoeckius, P. Smibert, R. Satija, Comprehensive Integration of Single-Cell Data. *Cell* **177**, 1888–1902.e21 (2019). doi:10.1016/j.cell.2019.05.031 Medline

56. Data for: R. Barilla, C. Berard, L. Sun, S. Sandhu, S. Zaghouani, K. S. Iyer, G. Altun, C. Su, J. Deguine, V. Singh, Y. Hou, K. Kusumakar, M. L. Rutlin, M. Rao, H. Zaghouani, H. N. Shi, R. J. Xavier, V. K. Kuchroo, Type 2 cytokines act on enteric sensory neurons to regulate neuropeptide-driven host defense, Figshare (2025); https://doi.org/10.6084/m9.figshare.c.7716548.
