# Supplementary material for: Type 2 cytokines act on enteric sensory neurons to regulate neuropeptide-driven host defense
Source: Science. Author manuscript; Available in PMC 2025 Nov 20. (PMC12632183; doi:10.1126/science.adn9850)

Supplemental fig. S7

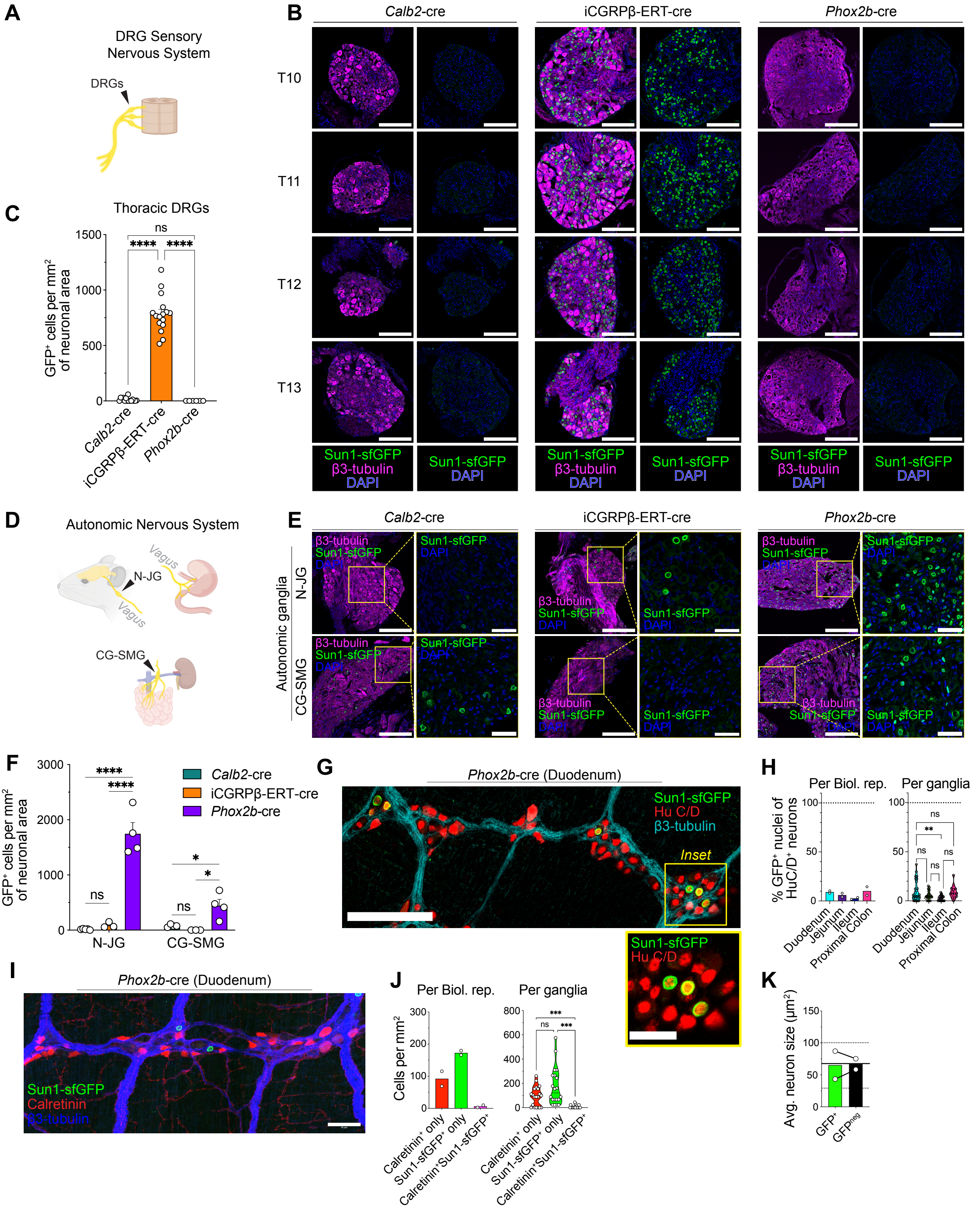

Supplement: S7_n2-no-error-bar_highres [file NIHMS2112150-supplement-S7_n2-no-error-bar_highres.pdf]
